# Halogenated Diterpenes with In Vitro Antitumor Activity from the Red Alga *Sphaerococcus coronopifolius*

**DOI:** 10.3390/md18010029

**Published:** 2019-12-29

**Authors:** Vangelis Smyrniotopoulos, Anna Cláudia de Andrade Tomaz, Maria de Fátima Vanderlei de Souza, Emídio Vasconcelos Leitão da Cunha, Robert Kiss, Véronique Mathieu, Efstathia Ioannou, Vassilios Roussis

**Affiliations:** 1 Section of Pharmacognosy and Chemistry of Natural Products, Department of Pharmacy, National and Kapodistrian University of Athens, Panepistimiopolis Zografou, 15771 Athens, Greece; esmiriniot@pharm.uoa.gr (V.S.); annacatomaz@gmail.com (A.C.d.A.T.); eioannou@pharm.uoa.gr (E.I.); 2 Postgraduate Program in Bioactive Natural and Synthetic Products, Health Sciences Center, Federal University of Paraíba, João Pessoa 58051-970, PB, Brazil; mfvanderlei@ltf.ufpb.br (M.d.F.V.d.S.); emidiovlcunha@gmail.com (E.V.L.d.C.); 3 Fonds National de la Recherche Scientifique, 1050 Bruxelles, Belgium; rkiss2012@gmail.com; 4 Department of Pharmacotherapy and Pharmaceutics, Université Libre de Bruxelles, Boulevard du Triomphe, 1050 Brussels, Belgium; vemathie@ulb.ac.be; 5 ULB Cancer Research Center, Université Libre de Bruxelles, Boulevard du Triomphe, 1050 Brussels, Belgium

**Keywords:** *Sphaerococcus coronopifolius*, red algae, halogenated diterpenes, structure elucidation, antitumor activity

## Abstract

Eight new (**1**–**8**) structurally diverse diterpenes featuring five different carbocycles were isolated from the organic extracts of the red alga *Sphaerococcus coronopifolius* collected from the coastline of the Ionian Sea in Greece. The structures of the new natural products, seven of which were halogenated, and the relative configuration of their stereocenters were determined on the basis of comprehensive spectroscopic analyses, including NMR and HRMS data. Compounds **5** and **8** were found to possess in vitro antitumor activity against one murine and five human cancer cell lines with mean IC_50_ values 15 and 16 μM, respectively.

## 1. Introduction

Nature has generously offered a plethora of chemical structures that have as such been used as therapeutics or have inspired the development of new pharmaceuticals. Marine macroalgae, one of the most primitive forms of life, biosynthesize secondary metabolites with broad structural diversity. A considerable number of these natural products are halogenated, as a consequence of the abundance of halogen ions in the seawater. Bromine predominantly, followed by chlorine substitution (even if the Cl/Br ratio is approx. 300 in the seawater) seems to enhance the bioactivity of compounds, while on the other hand, iodinated chemical structures are rare among secondary metabolites [1].

Cancer is one of the most serious, complicated to cure and frequently deadly diseases, and carries huge economic impact in modern societies. The estimated number of cancer cases in the world accounts for approximately 15% of worldwide mortality. Ominous predictions of a 68% increase in cancer incidents globally by 2030 necessitate the discovery of novel chemotherapeutic agents [2]. Over 60% of antineoplastic drugs, including approved pharmaceuticals and molecules under clinical evaluation, can be traced back to natural sources [3].

*Sphaerococcus coronopifolius* Stackhouse 1797, a bright, scarlet red seaweed, growing on rock subtidal habitats, with a wide distribution throughout the Mediterranean Sea and the East Atlantic Ocean, has shown to be a prolific source of structurally interesting diterpenes, most of which possess at least one bromine atom [4,5,6,7,8,9,10,11,12,13,14,15,16,17,18,19,20,21,22,23,24,25,26,27,28]. These secondary metabolites have exhibited a wide range of bioactivities, such as antibacterial [20,22,25,26,28,29], cytotoxic/antitumor [18,21,24,28], antimalarial [20], anti-inflammatory [30], and antifouling [27].

**Figure 1:**
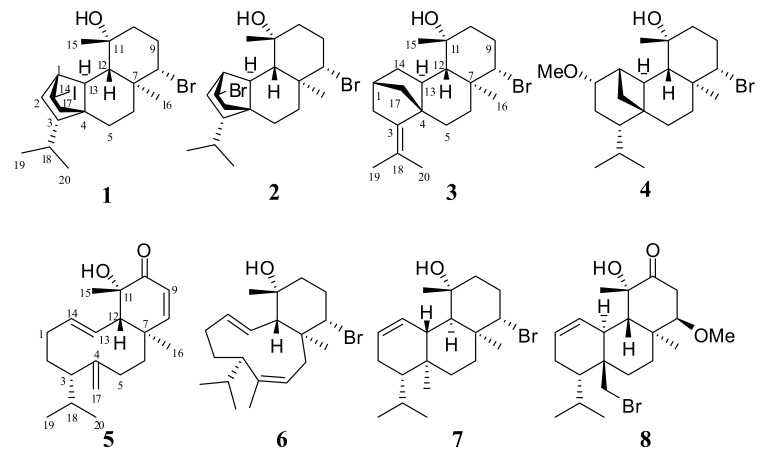
Chemical structures of compounds **1**–**8**.

As part of our ongoing interest in the discovery of novel bioactive metabolites of natural origin from marine organisms, we have focused on the chemical constituents of the red alga *S. coronopifolius*, which is sporadically encountered along the rocky Greek coastlines. A population of this alga previously collected in Liapades Bay at Corfu island afforded three structurally unique diterpenes, including spirosphaerol, anthrasphaerol and corfusphaeroxide [31]. Herein, we report the isolation and structure elucidation of eight new diterpene alcohols (**1**–**8**) (Figure 1) from another population collected later from another site in the Ionian Sea, including (i) four tetracyclic halogenated diterpenes, namely iodocoronol (**1**), the 14-iodo-substituted derivative of coronopifoliol [13], bromocoronol (**2**), the corresponding 14-bromo-substituted analogue, bromotetrasphaereniol (**3**), the Δ^3,18^ unsaturated 14-deoxy derivative of bromotetrasphaerol [14], and the methoxy derivative **4** of ioniol I [26], (ii) two bicyclic diterpenes, corotrienone (**5**) featuring the sphaerococcenol-like [5] α,β-unsaturated ketone analogue of bromocorodienol [12], and the double-bond positional isomer iso-bromocorodienol (**6**), and (iii) two brominated 6/6/6 tricyclic diterpenes, debromosphaerol (**7**), the unusual B/C *cis*-fused 17-debromo analogue of bromosphaerol [4], and the methoxy derivative **8** of 8-hydroxy-dihydro-sphaerococcenol [21]. The structures and relative stereochemistry of the isolated metabolites were established on the basis of 1D and 2D NMR, IR, UV, and HR-ESIMS data.

The isolated metabolites were evaluated for their in vitro inhibitory effect on the growth of one murine (B16F10 melanoma) and five human cancer cell lines (A549 lung cancer, Hs683 and U373 glioma, MCF7 breast cancer, SKMEL28 melanoma) and were found to display inhibitory concentrations by 50% (as compared to the control growth; IC_50_) in the range of 15–78 μM.

## 2. Results and Discussion

*S. coronopifolius* specimens were collected by scuba diving from Ai Giannis Bay (Parga, Greece) and the freeze-dried algal fronds were extracted with CH_2_Cl_2_/MeOH to afford an oily residue that was subjected to a series of chromatographic separations, including a combination of vacuum and flash column chromatography over silica gel and repeated C_18_ reversed and normal phase HPLC purifications, to afford compounds **1**–**8**.

**Figure 2:**
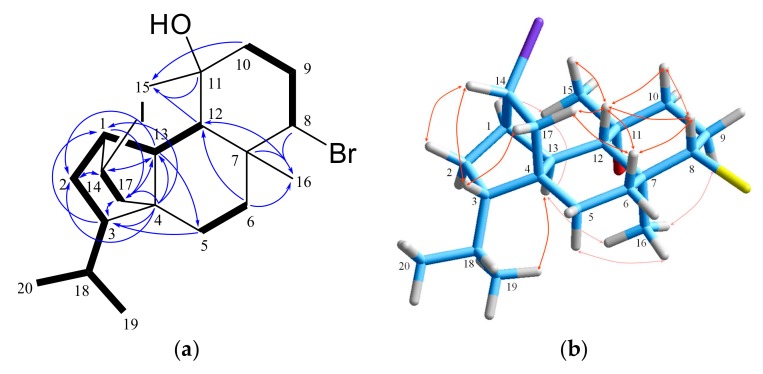
(**a**) COSY and key HMBC correlations and (**b**) important NOE interactions for iodocoronol (**1**).

Iodocoronol (**1**), presenting the molecular formula C_20_H_32_OBrI as established by HR-ESIMS, which exhibited isotopic pseudomolecular ion peaks [M−H]^−^ at *m*/*z* 493.0583 and 495.0563 with a ratio of 1:1, characteristic for the presence of one bromine atom in the molecule, was isolated as a colorless viscous liquid. Its ^13^C NMR spectrum revealed the resolved resonances of 20 carbons that on the basis of the HSQC-DEPT data were attributed to four methyls that are characteristic of the diterpenes produced by this algal species, comprising an isopropyl group (*δ*_H/C_ 0.85/23.4, 0.84/18.7), a carbinol methyl (*δ*_H/C_ 1.56/32.9) and an angular methyl group (*δ*_H/C_ 1.16/16.6), six methylenes, seven methines, including two halomethines (*δ*_H/C_ 4.09/68.5, 3.98/25.9), and three quaternary carbons, including an oxygenated non-protonated carbon (*δ*_C_ 73.6) (Table 1, Table 2 and Table S1). The four degrees of unsaturation necessitated the presence of four rings in the molecule. Comprehensive analysis of the COSY and HSQC data demonstrated the presence of one extended and three short spin systems (Figure 2). Diagnostic HMBC correlations of C-6, C-7, C-8 and C-12 with the angular methyl H_3_-16, and of C-10, C-11 and C-12 with the carbinol methyl H_3_-15, along with the cross-peaks of C-6 with H-12, of C-13 with H-5α, as well of C-4 with Η-5α and H-13 ascribed the decalin system, typical of the sphaerane class of diterpenes. Moreover, the combination of the HMBC correlations of C-1, C-3, C-4 and C-13 with the *sp*^3^ methylene H_2_-17, of C-17 with H-3, H-5α and H-13, of C-14 with H-1, H-2 and H-13, and of C-2 and C-13 with H-14, in conjunction with the long-range HMBC coupling network of the quaternary carbon C-4 with H-1, H-2 and H-3, and of C-5 with H-3 were suggestive of a structure comprising a 6/5/6/6 ring system, in agreement with the carbocycle of coronopifoliol [13]. While the ^1^H NMR spectrum of **1** resembled that of coronopifoliol [13], the major difference was observed in the pronounced shift of C-14 carbon resonance to lower frequencies (*δ*_C_ 25.9), as expected in the case of an iodo-substitution due to the electronegativity impact [32]. The relative configuration of the stereocenters of iodocoronol (**1**) was deciphered from the NOESY spectrum and the proton–proton coupling constants as 1R*,3S*,4S*,7S*,8S*,11R*,12S*,13R*,14R* (Figure 2), identical to that of coronopifoliol [13]. In particular, the large coupling constant values for H-6β, H-8, H-9α, H-10β, H-12 and H-13 placed these protons in axial positions in the decalin system, necessitating the *trans*-fusion of the cyclohexane rings. This was further confirmed by the NOEs observed between protons H-6β, H-8 and H-12, and by the cross-peaks of H-8 and H-12 with H-10β, of H-12 with H_3_-15, and of H-17a with both H-6β and H-12, in addition to the correlations of H_3_-16 with H-13, H-5α and H-9α. Furthermore, key NOE correlations between H-3, H-14 and H-17b, between H_3_-15 and H-1, H-12 and H-13, as well as of the latter with H_3_-19 confirmed the β-equatorial orientation of the carbinol methyl, and unambiguously secured the relative configuration around the stereogenic centers C-1, C-3 and C-4, thus establishing the structure of the new tetracyclic diterpene **1**. 

Bromocoronol (**2**) was obtained as a colorless oil and its molecular formula C_20_H_32_OBr_2_ was evidenced from the [M + H − H_2_O]^+^ ion at *m*/*z* 429.0775 by HR-ESIMS analysis. The ions at *m*/*z* 429.0775:431.0753:433.0732 (1:2:1) [M + H − H_2_O]^+^, and *m*/*z* 349.1514:351.1493 (1:1) [M + H − H_2_O − HBr]^+^ indicated the presence of two bromine atoms and a hydroxyl group, attributed to the respective bromomethine signals at *δ*_H/C_ 4.07/68.6, and 4.03/52.5, along with the oxygenated quaternary carbon resonating at *δ*_C_ 73.5 (Table 1, Table 2 and Table S2). The carbocycle of coronopifoliol [13], as in the case of **1**, was assigned to bromocoronol (**2**) after in-depth analyses of its 1D and 2D NMR spectra that exhibited close similarities with those of **1**. The position of the bromine atom at C-14 was secured by the intense correlations observed in COSY and HSQC-TOCSY spectra between H-14 and H_2_-17, in combination with the HMBC interactions of C-14 with H-1, H_2_-2 and H_2_-17, of C-3 and C-4 with H-17a, of C-13 with H-17b, and of C-17 with H-1. Careful examination of ^13^C and ^1^H NMR chemical shifts and coupling constants of all protons also supported the retention of relative stereochemistry in all stereogenic centers of bromocoronol (**2**), including C-14. This was further confirmed by extensive analysis of the NOESY spectrum, and the NOE interactions between H-3 and both H-14 and H-17b, and of H-17a/H-6β and H-17a/H-12.

**Figure 3:**
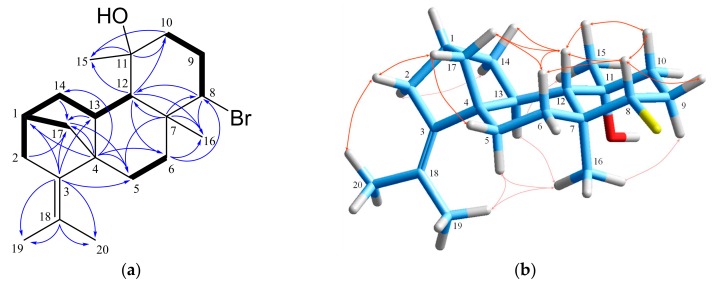
(**a**) COSY and key HMBC correlations and (**b**) important NOE interactions for bromotetrasphaereniol (**3**).

Compound **3** was isolated as a colorless liquid. The HR-ESIMS measurement of bromotetrasphaereniol (**3**) at *m*/*z* 349.1531 suggested a molecular formula of C_20_H_31_OBr, requiring five degrees of unsaturation. Its ^1^H and ^13^C NMR spectra (Table 1, Table 2 and Table S3) displayed frequencies assigned with the information from the HSQC-DEPT spectrum to two olefinic methyls (*δ*_H/C_ 1.84/20.3, 1.57/23.9) on a tetrasubstituted double bond (*δ*_C_ 119.9, 139.0), two singlet methyls (*δ*_H/C_1.14/32.7, 1.18/16.9), four methines, including a bromomethine (*δ*_H/C_ 3.97/68.5), seven methylenes with non-equivalent protons, and three quaternary angular carbons, including one oxygenated (*δ*_C_ 72.9). The above-mentioned data accounted for one degree of unsaturation, suggesting a tetracyclic diterpene skeleton. Detailed interpretation of the COSY spectrum revealed the presence of three spin systems, while their connectivities and corroboration of their substructures were established on the basis of HMBC analysis (Figure 3). The heteronuclear long-range correlations observed from C-10, C-11 and C-12 to the methyl H_3_-15, from C-11 to H-10β, and from C-12 to H-10α demonstrated the linkage of C-10 and C-12 through the carbinol quaternary carbon C-11. The connection of all spin systems through the quaternary carbon C-7 was validated by the correlations of C-6, C-7, C-8 and C-12 with H_3_-16, of C-7 with H-5β, H-8 and H-12, as well as by those of C-8 with H-12, of C-6with H-8, and of C-12 with H-6α. The cross-peaks displayed in the HMBC spectrum between C-4 and H-1, H_2_-5, H-6α and H-14α, and also between the quaternary olefinic carbon C-3 and H-1, H-5α and H-13 confirmed a perhydrophenanthrene core. The tetracyclic system of the bromotetrasphaerol class of diterpenes [14], comprising C-17 as the bridge carbon between C-1 and C-4, was evident by the HMBC correlations exhibited from C-17 to H-5α, from C-2 and C-4 to H_2_-17, and from C-3, C-13 and C-14 to H-17b. Finally, the HMBC interconnection network of both C-3 and C-18 with the methyls H_3_-19 and H_3_-20 secured the position of the isopropylidene group, thus completing the planar structure of the molecule. The relative configuration of the stereogenic centers of bromotetrasphaereniol (**3**) was determined on the basis of NOESY data (Figure 3). ^1^H NMR coupling constant pattern analyses and NOESY correlations of H-6β with H-8, and of the latter with H-10β and H-12, in addition to the NOE interactions of H_3_-16 with H-5α, H-6α, H-9α and H-13 established the *trans*-diaxial relationship of the couples H-5α/H-6β, H-8/H-9α, H-9α/H-10β, and H-12/H-13, with H-6β, H-8, H-10β and H-12 being β-oriented, while H-5α, H-9α, H-13 and methyl H_3_-16 being α-oriented on the opposite side. The β-equatorial orientation of the carbinol methyl H_3_-15 was deduced by the NOESY cross-peaks observed between H_3_-15 and H-10β, H-12 and H-13. Lastly, the NOESY correlations observed from H-17a to H-6β, H-12 and H-14β, from H-17b to H-2β and H-5β, and complimentary from H-2α to H-14α and from H-12 to H-14β secured the configuration at C-1 and C-4, therefore finalizing the relative configuration of **3** as 1R*,4R*,7S*,8S*,11R*,12S*,13S*. 

**Figure 4:**
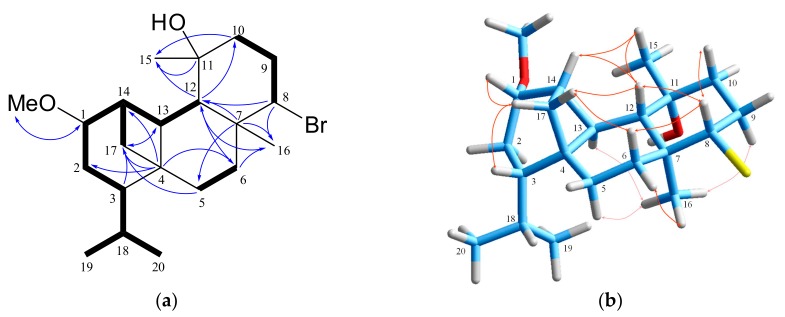
(**a**) COSY and key HMBC correlations and (**b**) important NOE interactions for 1-methoxy-ioniol I (**4**).

Compound **4**, displaying the molecular formula C_21_H_35_O_2_Br as suggested by HR-ESIMS data, was confidently assigned on the basis of 1D and 2D NMR (COSY, HSQC, HMBC) spectra analyses as the methoxy derivative of the known metabolite ioniol I [26]. The ^1^H and ^13^C NMR data (Table 1, Table 2 and Table S4) of the two compounds were almost identical, with the major difference being the addition of a methoxy group (*δ*_H/C_ 3.30/55.9) in the spectra of **4**. Its position at C-1 was evident from the diagnostic HMBC correlations of C-1 with the methoxy protons H_3_-21, and of C-21 with H-1 (Figure 4), as well as from the characteristic chemical shift effects of methoxylation on oxymethine C-1 (*δ*_H/C_ 3.58/81.9 in **4**, *δ*_H/C_ 4.14/72.8 in ioniol I [26]). Noticeable shift effects were also observed for the neighboring methylene at C-2 (*δ*_H/C_ 2.10, 1.60/25.1 in **4**, *δ*_H/C_ 2.14, 1.60/27.6 in ioniol I [26]) and methine at C-14 (*δ*_H/C_ 2.27/40.9 in **4**, *δ*_H/C_ 2.12/45.4 in ioniol I [26]). Retention of the relative configuration for all chiral carbons of 1-methoxy-ioniol I (**4**), including C-1, was deduced from analysis of the NOESY spectrum (Figure 4). Thus, prominent NOE correlations from H-1 to H-17b, from the latter to H-3, from H-17a to H-6β and H-12, from these to H-8, and from H-8 to H-9β, along with the cross-peaks observed among H-12, H-14 and H_3_-15, and also between H_3_-16 with H-5α, H-6α, H-9α, and H-13 confirmed the relative configuration of **4** as 1S*,3S*,4S*,7S*,8S*,11R*,12S*,13S*,14S*.

**Figure 5:**
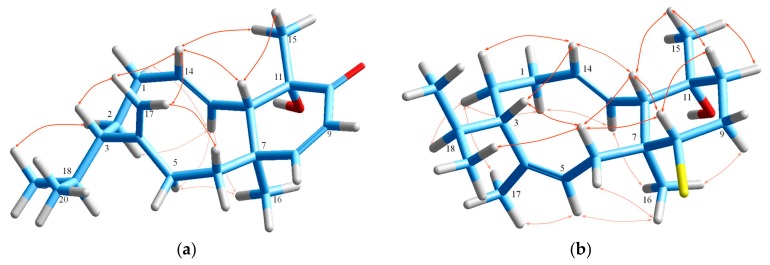
Important NOE interactions for (**a**) corotrienone (**5**) and (**b**) iso-bromocorodienol (**6**).

Positive ion-mode HR-ESIMS measurement of corotrienone (**5**) at *m*/*z* 325.2132 [M + Na]^+^ indicated the molecular formula C_20_H_30_O_2_, consistent with a hydrogen deficiency index of six. Strong IR bands at 3482 and 1673 cm^−1^ were suggestive of hydroxy and carbonyl functionalities in the molecule. Comprehensive examination of the ^1^H and ^13^C NMR and HSQC-DEPT spectra revealed the presence of a carbonyl (*δ*_C_ 200.6), an exomethylene (*δ*_H/C_ 4.87, 4.76/112.3, *δ*_C_ 153.3), four additional protonated olefinic carbons (*δ*_H/C_ 6.81/164.8, 5.92/124.6, 5.72/136.1, 5.60/124.2), two *sp*^3^ quaternary carbons, including one oxygenated (*δ*_C_ 73.5), two methyls attached on quaternary carbons (*δ*_H/C_ 1.20/25.2, 1.27/20.4), two non-equivalent methyls of an isopropyl group (*δ*_H/C_ 0.85/20.7, 0.76/21.5), four *sp*^3^ methylenes and two aliphatic methines (Table 1, Table 3 and Table S5). The remaining two degrees of unsaturation were attributed to a bicyclic structure. Three isolated spin systems were differentiated from the analysis of the COSY spectrum. The connection of C-3 and C-5 through the exomethylene carbon C-4 was secured by the cross-peaks observed in the HMBC spectrum from C-3 and C-5 to H_2_-17, from C-17 to H-3 and H-5α, and from C-4 to H-3, H-5β and H_2_-6. Moreover, the HMBC correlations of C-6, C-7, C-8 and C-12 with H_3_-16, of C-7 with H-9 and H-12, of C-12 with H_2_-6, of C-6 and C-12 with H-8, and of C-7 with H-9 led to the connection of the three spin systems through the quaternary carbon C-7, resulting in the formation of a ten-membered carbon ring, also present in bromocorodienol [12]. The presence of a fused cyclohexene ring containing the α,β-conjugated enone system previously found in sphaerococcenol [5], indicated by the downfield shift of the β-olefinic carbon (*δ*_H/C_ 6.81/164.8) and the shielding of the carbonyl to lower frequencies (*δ*_C_ 200.6), was validated by the HMBC correlations from C-10, C-11 and C-12 to H_3_-15, from C-10 to H-8, and from C-11 to H-9. The assignment of the relative configuration of the four chiral carbons of the molecule was deduced from extended analysis of the NOESY spectrum (Figure 5). Briefly, the NOE interactions of H-3/H-17a, H-17a/H-14, H-14/H-12, H-17b/H-12, and H-12/H_3_-15 secured the configuration at C-3, C-11, and C-12 as all-S*. The assignment of C-7 as R* was dictated by the observed NOESY correlations of H-13/H_3_-16, H-5a/H_3_-16, H-13/H-5a and H-13/H-1α. Furthermore, the aforementioned NOESY cross-peaks, along with the large coupling constant observed between H-13 and H-14 (*J* = 15.8 Hz) confirmed the *E* geometry of Δ^13^.

Compound **6** was isolated a colorless oil possessing the molecular formula C_20_H_33_OBr that derived from the HR-ESIMS measurement and ^13^C NMR data, dictating four degrees of unsaturation. The 1:1 isotope clusters observed in the MS spectra (*m*/*z* 351.1676:353.1656) were indicative for the presence of a bromine atom. The ^1^H and ^13^C NMR, COSY and HSQC-DEPT spectra of **6** (Table 1, Table 3 and Table S6) exhibited the characteristic signals of two double bonds, one disubstituted (*δ*_H/C_ 5.27/128.1, 5.18/133.0) and one trisubstituted (*δ*_H/C_ 133.3, 5.27/125.7), a bromomethine (*δ*_H/C_ 4.00/68.0), and two *sp*^3^ quaternary carbons, including one oxygenated (*δ*_C_ 71.7), along with three more aliphatic methines, five methylenes, and five methyls, comprising one vinyl (*δ*_H/C_ 1.52/19.1), two secondary (*δ*_H/C_ 0.90/21.1, 0.67/21.3) and two tertiary methyls (*δ*_H/C_ 1.06/30.9, 1.28/15.2). Comparison of the NMR data of **6** with reported values for bromocorodienol [12] showed a close similarity and led to the assignment of the structure as its Δ^4,5^ isomer. The methyl on the trisubstituted double bond was positioned next to the isopropyl group, thus linking C-3 and C-5 via the quaternary *sp*^2^ carbon C-4, in agreement with the HMBC correlations of C-3, C-4 and C-5 with H_3_-17 and of C-4 with H-2β and H_2_-6. The connection of C-10 to C-12, accomplished through a methyl carbinol group was verified by the cross-peaks from C-10, C-11 and C-12 to H_3_-15, and from C-12 to H-10α. Moreover, the HMBC correlations of C-6, C-7, C-8 and C-12 with the angular methyl H_3_-16, of C-6, C-7, C-8 and C-16 with H-12, of C-12 with H-6β, and of C-7 with both H_2_-6 connected all three spin systems in the bicyclic diterpene skeleton. The relative configurations of the stereogenic centers in compound **6** were determined on the basis of NOESY experiments and ^1^H NMR coupling constants (Figure 5). The prominent spatial NOE interactions observed between the olefinic H-5 and H_3_-17 led to the assignment of the Δ^4,5^ double bond as *Z*, a fact that was also supported by the ^13^C chemical shift of methyl C-17 (*δ*_C_ 19.1), exhibiting an upfield shift of 3–6 ppm compared to similar *Z*-trisubstituted bonds due to the γ-substitution of the isopropyl group [33]. Conversely, the *E* geometry was established for Δ^13^ double bond based on the NOESY correlations of H-14 with H-12, of H-12 with H-6β (confirmed by 1D NOE experiments) and H-8, and of the latter with H-6β, H-9β and H-10β, as well as on cross-peaks of H-13 with H_3_-16, and of H_3_-16 with both H-6α and H-9α, that also confirmed the *trans*-fusion of the two rings, assigning H-6β, H-8, H-9β and H-10β a β-orientation, while methyl H_3_-16 together with protons H-6α and H-9α were α-oriented. The β-equatorial orientation of methyl H_3_-15 was confirmed by the correlations of H_3_-15 with H-12, H-10α and Η-10β. The S*-configuration at C-3 was secured by the NOESY correlations of H-3 with H-14 and H-6β, therefore defining the structure of iso-bromocorodienol (**6**). 

**Figure 6:**
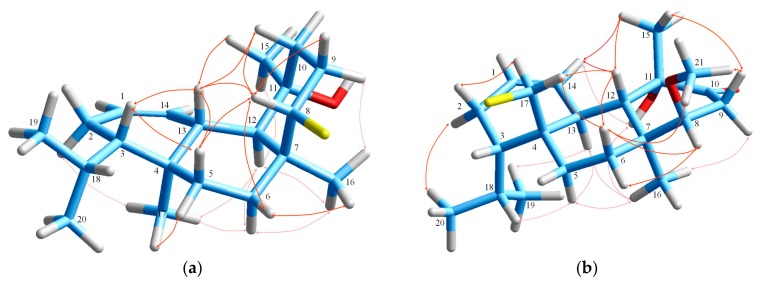
Important NOE interactions for (**a**) debromosphaerol (**7**) and (**b**) 8-methoxy-dihydro-sphaerococcenol (**8**).

Debromosphaerol (**7**) isolated as a colorless oily substance, displayed the fragment ion peak at *m*/*z* 351.1674 in the HR-ESIMS spectrum corresponding to [M + H − H_2_O]^+^ and consistent with the molecular formula C_20_H_33_OBr, which dictated four double bond equivalents. Analysis of the NMR data (Table 1, Table 3 and Table S7) revealed the presence of a disubstituted double bond (*δ*_H/C_ 5.80/131.9, 5.55/127.0) and a bromomethine (*δ*_H/C_ 4.55/60.6), along with signals attributed to five methyl groups, three displayed as singlets (*δ*_H/C_ 1.33/35.4, 1.34/28.1, 0.77/17.7) and two as doublets (*δ*_H/C_ 0.86/23.4, 0.78/16.6), as well as five methylene groups, four more methines, and three quaternary carbons, including one oxygenated (*δ*_C_ 75.7). Interpretation of the COSY spectrum confirmed the existence of four spin systems. The correlations observed in the HMBC spectrum from C-3, C-4, C-5 and C-13 to H_3_-17, from C-17 to H-3 and H-5β, from C-4 to H-3, H-6α, H-12 and H-14, and from C-13 to H-3 and H-5α established a cyclohexene ring in the molecule. The substitution of an isopropyl group at C-3 was verified by the HMBC cross-peaks of C-3 with H_3_-19 and H_3_-20. A second six-membered ring, 4,13-fused to the cyclohexene ring, was validated by the correlations of C-6, C-7, C-8 and C-12 with H_3_-16, of C-16 with H-8, of C-7 and C-8 with H-12, and of C-8 with H-6β. Furthermore, the cross-peaks observed from C-10, C-11 and C-12 to H_3_-15 and from C-11 to H-12 revealed an additional cyclohexane ring in a tricyclic perhydrophenanthrene core structure. The relative configuration of the stereogenic centers in debromosphaerol (**7**) was determined on the basis of thorough analyses of 1D and 2D NOE experiments, and *J* values observed in the ^1^H NMR spectrum (Figure 6). Τhe NOESY correlations among H-3, H-5β and H-13, along with the cross-peaks of Η_3_-17 with H-2α, Η-5α, H-6α and Η-12 secured the *trans*-fusion between the cyclohexene and middle cyclohexane ring. The additional NOESY interactions, among H-6α, Η-12 and Η_3_-16, and between the latter and H-9α, as well as the correlations of H-8 with H-5β, H-6β, H-9β, H-10β and H-13, and of H-13 with H-10β, established the uncommon *cis*-fusion of the two cyclohexane rings, previously reported for coronone [24]. Moreover, the β-equatorial orientation of Η_3_-15 was evident from its synchronous NOEs observed with H-12 and H-13, thus permitting the assignment of the relative configuration of debromosphaerol (**7**).

HR-ESIMS analysis of compound **8** indicated the molecular formula C_21_H_33_O_3_Br, designating five degrees of unsaturation. Detailed examination of the 1D and 2D NMR data of **8** (Table 1, Table 3 and Table S8) revealed remarkable structural similarity with that of 8-hydroxy-dihydro-sphaerococcenol [21]. The additional singlet integrating for three protons at *δ*_H_ 3.36 and the signal at *δ*_C_ 57.5 in the NMR spectra of **8** suggested that it was a methoxy derivative of hydroxy-dihydro-sphaerococcenol [21]. The site of methoxylation was unambiguously assigned at C-8 based on the strong cross-peak observed in HMBC spectrum between C-8 and the methoxy protons H_3_-21, as well as on the downfield shift by 9.3 ppm of C-8 (*δ*_C_ 83.8) and the upfield shift by 0.65 ppm of H-8 (*δ*_H_ 3.09). As expected, the substitution proceeded with retention of configuration at all stereogenic centers in the molecule, including C-8, a fact that was verified by the NOE interactions and the ^1^H NMR coupling constant patterns dictating the 3S*,4S*,7S*,8R*,11S*,12S*,13S* configuration (Figure 6). It is noteworthy that the carbonyl-bearing ring adopts an unusual pseudo-boat conformation that is more stable than the corresponding chair conformer (calculated energy 54.14 Kcal/mol for pseudo-boat conformer versus 59.57 Kcal/mol for chair conformer, see Supplementary Materials), thus justifying both NOESY interactions between H-9α/Η_3_-16 and H-9β/Η_3_-15, and also the observed *J* values of the ABX pattern indicating a dihedral angle of almost 90° between H-8 and H-9β.

Compounds **1**–**8** were evaluated for their in vitro growth inhibitory activity against one murine and five human tumor cell lines (Table 4). The 10-oxo compounds corotrienone (**5**) and 8-methoxy-dihydro-sphaerococcenol (**8**) displayed worth-noting in vitro antitumor activity against all tested cell lines, with IC_50_ values ranging from 8 to 23 and from 10 to 25 μM, respectively. Iso-bromocorodienol (**6**) and debromosphaerol (**7**) exhibited lower activities in the range 32–58 and 27–72 μM, respectively, while the tetracyclic diterpenes **1**–**4** showed even lower activity, with bromocoronol (**2**) and bromotetrasphaereniol (**3**) being active against the whole panel (IC_50_ values of 40-86 μM) and iodocoronol (**1**) and 1-methoxy-ioniol I (**4**) been mildly active against A549, Hs683, MCF7 and B16F10 cell lines.

## 3. Materials and Methods

### 3.1. General Experimental Procedures

Optical rotations were measured at the sodium D line (589.3 nm) at 20 °C on a PerkinElmer model 341 polarimeter (PerkinElmer Instruments, Norwalk, CT, USA) with a 10 cm cell. UV spectra were obtained in spectroscopic grade MeOH or CHCl_3_ on a Shimadzu UV-160A spectrophotometer (Shimadzu Europa GmbH, Duisburg, Germany). IR spectra were obtained using a Paragon 500 PerkinElmer spectrometer (PerkinElmer Instruments, Norwalk, CT, USA). NMR spectra were recorded using Bruker AC 200 (Bruker BioSpin GmbH, Rheinstetten, Germany), Varian 300 (Varian, Inc., Palo Alto, CA, USA), Bruker DRX 400 (Bruker BioSpin GmbH, Rheinstetten, Germany) and Varian 600 (Varian, Inc., Palo Alto, CA, USA). Unless otherwise specified, chemical shifts are expressed in ppm with reference to the solvent signals, and *J* values in Hz. High-resolution ESI mass spectra were measured in positive mode on a Thermo Scientific LTQ Orbitrap Velos mass spectrometer (Thermo Fisher Scientific, Bremen, Germany). Low-resolution EI and CI mass spectra were measured on a Thermo Electron Corporation DSQ mass spectrometer (Thermo Electron Corporation, Austin, TX, USA) using a direct-exposure probe with CH_4_ as reagent gas. Vacuum liquid chromatographic separations were performed with Kieselgel 60 (Merck, Darmstadt, Germany), gravity column chromatographic separations were performed with Kieselgel 60H (Merck, Darmstadt, Germany), thin layer chromatography (TLC) was performed with Kieselgel 60 F_254_ aluminum support plates (Merck, Darmstadt, Germany) and spots were visualized after spraying with 15% (v/v) of 96% H_2_SO_4_ in MeOH followed by heating. HPLC separations were conducted on an Agilent 1100 model (Agilent Technologies, Waldbronn, Germany) equipped with refractive index detector and a Kromasil 100 C_18_ (250 × 8 mm, 5 μm, MZ-Analysentechnik GmbH, Mainz, Germany) HPLC reversed phase column or a Kromasil 100 SIL (250×8 mm, 5 μm, MZ-Analysentechnik GmbH, Mainz, Germany) HPLC normal phase column. The 3D structures were generated and optimised in HyperChem™ Professional 8.0.8 (Hypercube, Inc., Gainesville, FL, USA) molecular modeling and simulation software (force field: MM+; optimization algorithm: Polak-Ribiere).

### 3.2. Biological Material

*S. coronopifolius* was collected by scuba diving in Ai Giannis Bay, Parga, Greece, at a depth of 10–22 m in September 2010. The alga was washed with seawater, immediately frozen, transferred to the laboratory and freeze-dried. A voucher specimen of the alga has been deposited at the Herbarium of the Section of Pharmacognosy and Chemistry of Natural Products, Department of Pharmacy, National and Kapodistrian University of Athens (ATPH/MP0288).

### 3.3. Extraction and Isolation

Freeze-dried algal material (125.3 g dry weight) was extracted with mixtures of CH_2_Cl_2_/MeOH (3/1) at room temperature. Evaporation of the solvents in vacuo afforded a dark green residue (3.94 g), which was subjected to vacuum liquid chromatography over silica gel, using a 2% step gradient elution of cyclohexane/EtOAc. Fraction F4 (4% EtOAc in cyclohexane, 2.19 g) was subjected to gravity column chromatography over silica gel using a 1% step gradient of cyclohexane/EtOAc. Fraction F4.3 (8% EtOAc in cyclohexane, 0.99 g) was further separated by reversed-phase HPLC (CH_3_CN 100%). Fraction F4.3.9 (11.7 mg) was purified by normal phase HPLC (15% EtOAc in cyclohexane) to yield **6** (2.0 mg). Fraction F4.4 (9% EtOAc in cyclohexane, 0.58 g) was further fractionated by non-aqueous reversed phase HPLC (MeOH 100%). Fractions F4.4.5 (5.1 mg) and F4.4.9 (9.4 mg) were separately purified by reversed-phase HPLC (MeOH 100%) and normal phase HPLC (5% EtOAc in cyclohexane), respectively, to afford **3** (0.8 mg) and **8** (4.7 mg). Fraction F4.5 (10% EtOAc in cyclohexane, 87.4 mg) was subjected to reversed-phase HPLC (MeOH 100%) to give pure **7** (2.4 mg). Compound **2** (3.5 mg) was isolated from fraction F4.5.11 (4.3 mg) by repeated reversed-phase HPLC (MeOH 100%). Fraction F5 (5% EtOAc in cyclohexane, 156.3 mg) was subjected to reversed-phase HPLC (MeOH 100%) to yield **1** (1.4 mg). Fraction F7 (10% EtOAc in cyclohexane, 62.7 mg) was subjected to reversed-phase HPLC (MeOH 100%) to give fraction F7.4 (3.1 mg), which was purified by reversed-phase HPLC (CH_3_CN 100%) to afford **5** (1.6 mg). Fraction F11 (20% EtOAc in cyclohexane, 112.0 mg) was further separated by reversed-phase HPLC (CH_3_CN 100%) to yield pure **4** (1.2 mg).

Iodocoronol (**1**): Colorless oil; ${\left[\alpha \right]}_{D}^{20}$ −60.2 (*c* 0.09, CHCl_3_); UV (CHCl_3_) *λ*_max_ (log *ε*) 258 (2.95); IR (thin film) *ν*_max_ 3468 (O–H), 2942 (C–H) cm^−1^; NMR data (CDCl_3_), see Table 1, Table 2 and Table S1; HR-ESIMS *m*/*z* 493.0583 [M − H]^−^ (calcd. for C_20_H_31_OBrI, 493.0608).

Bromocoronol (**2**): Colorless oil; ${\left[\alpha \right]}_{D}^{20}$ −67.1 (*c* 0.20, CHCl_3_); UV (CHCl_3_) *λ*_max_ (log *ε*) 240 (2.64); IR (thin film) *ν*_max_ 3452 (O–H), 2957 (C–H) cm^−1^; NMR data (CDCl_3_), see Table 1, Table 2 and Table S2; HR-ESIMS *m*/*z* 429.0775 [M + H − H_2_O]^+^ (calcd. for C_20_H_31_Br_2_, 429.0787).

Bromotetrasphaereniol (**3**): Colorless oil; ${\left[\alpha \right]}_{D}^{20}$ +6.1 (*c* 0.03, CHCl_3_); UV (CHCl_3_) *λ*_max_ (log *ε*) 239 (2.49); IR (thin film) *ν*_max_ 3397 (O–H), 2952 (C–H) cm^−1^; NMR data (CDCl_3_), see Table 1, Table 2 and Table S3; HR-ESIMS *m*/*z* 349.1531 [M + H − H_2_O]^+^ (calcd. for C_20_H_30_Br, 349.1525).

1-Methoxy-ioniol I (**4**): Colorless oil; ${\left[\alpha \right]}_{D}^{20}$ −43.6 (*c* 0.09, CHCl_3_); UV (CHCl_3_) *λ*_max_ (log *ε*) 271 (2.68), 334 (2.23), 420 (1.96); IR (thin film) *ν*_max_ 3440 (O–H), 2936 (C–H), 1097 (C–O) cm^−1^; NMR data (CDCl_3_), see Table 1, Table 2 and Table S4; HR-ESIMS *m*/*z* 421.1717 [M + Na]^+^ (calcd. for C_21_H_35_O_2_BrNa, 421.1713).

Corotrienone (**5**): Colorless oil; ${\left[\alpha \right]}_{D}^{20}$ −105.0 (*c* 0.13, CHCl_3_); UV (CHCl_3_) *λ*_max_ (log *ε*) 249 (3.27), 340 (1.96); IR (thin film) *ν*_max_ 3482 (O–H), 2930 (C–H), 1673 (C=O) cm^−1^; NMR data (CDCl_3_), see Table 1, Table 2 and Table S5; HR-ESIMS *m*/*z* 325.2132 [M + Na]^+^ (calcd. for C_20_H_30_O_2_Na, 325.2138).

iso-Bromocorodienol (**6**): Colorless oil; ${\left[\alpha \right]}_{D}^{20}$ +27.3 (*c* 0.14, CHCl_3_); UV (CHCl_3_) *λ*_max_ (log *ε*) 240 (2.90); IR (thin film) *ν*_max_ 3389 (O–H), 2936 (C–H) cm^−1^; NMR data (CDCl_3_), see Table 1, Table 2 and Table S6; HR-ESIMS *m*/*z* 351.1676 [M + H − H_2_O]^+^ (calcd. for C_20_H_32_Br, 351.1682).

Debromosphaerol (**7**): Colorless oil; ${\left[\alpha \right]}_{D}^{20}$ +56.9 (*c* 0.12, CHCl_3_); UV (CHCl_3_) *λ*_max_ (log *ε*) 242 (2.70); IR (thin film) *ν*_max_ 3478 (O–H), 2952 (C–H) cm^−1^; NMR data (CDCl_3_), see Table 1, Table 2 and Table S7; HR-ESIMS *m*/*z* 351.1674 [M + H − H_2_O]^+^ (calcd. for C_20_H_32_Br, 351.1682).

8-Methoxy-dihydro-sphaerococcenol (**8**): Colorless oil; ${\left[\alpha \right]}_{D}^{20}$ −136.7 (*c* 0.37, CHCl_3_); UV (CHCl_3_) *λ*_max_ (log *ε*) 238 (2.59), 274 (2.22), 327 (1.79); IR (thin film) *ν*_max_ 3468 (O–H), 2932 (C–H), 1703 (C=O) cm^−1^; NMR data (CDCl_3_), see Table 1, Table 2 and Table S8; HR-ESIMS *m*/*z* 435.1498 [M + Na]^+^ (calcd. for C_21_H_33_O_3_BrNa, 435.1511).

### 3.4. Evaluation of In Vitro Growth Inhibitory Activity

Compounds **1**–**8** were evaluated for their in vitro growth inhibitory activity against five human tumor cell lines, including the A549 non-small cell lung cancer (ACC107, Deutsche Sammlung von Mikroorganismen und Zellkulturen, Braunschweig, Germany), the U373 glioblastoma (ECACC 08061901, European Collection of Authenticated Cell Cultures, Salisbury, UK), the Hs683 oligodendroglioma (ATCC HTB-138, American Type Culture Collection, Manassas, VA, USA), the MCF7 breast cancer (ATCC HTB22) and the SKMEL28 melanoma (ATCC HTB72) cell lines, and against the B16F10 murine melanoma (ATCC CRL 6475) cell line. The inhibitory growth activity of the compounds under study was determined by means of the MTT [3-(4,5-dimethylthiazol-2-yl)-2,5-diphenyltetrazolium bromide] colorimetric assay [34,35]. The cancer cells were cultured during three days in the presence of the compounds and the data were reported as mean IC_50_ values calculated on the sextuplicates of the experiment conducted once for each compound and for each cell line.

## 4. Conclusions

Iodination occurs more frequently in brown algae metabolites, but only less than 1% of secondary metabolites from brown algae contain bromine or chlorine in contrast with as much as 90% and 7% of red and green algal compounds, respectively [1]. In our continuing investigation aimed at the bioactivity screening and isolation of bioactive metabolites from the Greek seas, eight new structurally diverse halogenated diterpenes (**1**–**8**) were isolated from the red alga *S. coronopifolius*. Their structures and relative configurations were determined on the basis of their spectroscopic data (mainly NMR and MS). The isolated metabolites were evaluated for their in vitro antitumor activity against one murine and five human cancer cell lines, exhibiting IC_50_ growth inhibitory concentrations ranging between 15 and 78 μM.

## Figures and Tables

**Table 1:** ^13^C NMR data (*δ* in ppm, multiplicity) of compounds **1**–**8**.

No.	1 ^a^	2 ^a^	3 ^b^	4 ^a^	5 ^a^	6 ^a^	7 ^a^	8 ^a^
1	49.4 (d)	49.3 (d)	34.7 (d)	81.9 (d)	28.1 (t)	29.7 (t)	127.0 (d)	127.7 (d)
2	35.6 (t)	34.0 (t)	41.0 (t)	25.1 (t)	32.2 (t)	24.8 (t)	23.3 (t)	22.7 (t)
3	48.8 (d)	48.9 (d)	139.0 (q)	48.3 (d)	55.8 (d)	44.4 (d)	52.0 (d)	42.0 (d)
4	51.9 (q)	51.3 (q)	52.8 (q)	43.5 (q)	153.3 (q)	133.3 (q)	38.2 (q)	40.1 (q)
5	22.8 (t)	22.9 (t)	24.4 (t)	24.0 (t)	25.0 (t)	125.7 (d)	31.6 (t)	24.7 (t)
6	37.8 (t)	37.7 (t)	38.7 (t)	37.0 (t)	39.3 (t)	40.1 (t)	34.8 (t)	29.1 (t)
7	41.5 (q)	41.4 (q)	41.0 (q)	39.3 (q)	41.4 (q)	44.7 (q)	39.5 (q)	39.8 (q)
8	68.5 (d)	68.6 (d)	68.5 (d)	68.7 (d)	164.8 (d)	68.0 (d)	60.6 (d)	83.8 (d)
9	31.0 (t)	30.9 (t)	30.9 (t)	30.6 (t)	124.6 (d)	31.1 (t)	30.9 (t)	38.9 (t)
10	43.6 (t)	43.7 (t)	43.3 (t)	42.5 (t)	200.6 (q)	40.8 (t)	38.5 (t)	216.9 (q)
11	73.6 (q)	73.5 (q)	72.9 (q)	72.6 (q)	73.5 (q)	71.7 (q)	75.7 (q)	76.3 (q)
12	47.6 (d)	47.9 (d)	56.4 (d)	52.0 (d)	58.5 (d)	62.3 (d)	53.6 (d)	42.9 (d)
13	44.8 (d)	44.5 (d)	39.0 (d)	31.8 (d)	124.2 (d)	128.1 (d)	45.4 (d)	35.4 (d)
14	25.9 (d)	52.5 (d)	43.1 (t)	40.9 (d)	136.1 (d)	133.0 (d)	131.9 (d)	129.0 (d)
15	32.9 (s)	33.2 (s)	32.7 (s)	30.3 (s)	25.2 (s)	30.9 (s)	35.4 (s)	31.2 (s)
16	16.6 (s)	16.3 (s)	16.9 (s)	16.0 (s)	20.4 (s)	15.2 (s)	28.1 (s)	17.2 (s)
17	50.2 (t)	48.6 (t)	43.9 (t)	34.2 (t)	112.3 (t)	19.1 (s)	17.7 (s)	40.6 (t)
18	28.3 (d)	28.3 (d)	119.9 (q)	27.6 (d)	29.9 (d)	30.4 (d)	26.5 (d)	25.8 (d)
19	23.4 (s)	23.4 (s)	20.3 (s)	15.8 (s)	20.7 (s)	21.1 (s)	23.4 (s)	19.3 (s)
20	18.7 (s)	18.8 (s)	23.9 (s)	22.6 (s)	21.5 (s)	21.3 (s)	16.6 (s)	25.8 (s)
OMe	–	–	–	55.9 (s)	–	–	–	57.5 (s)

^a^ Recorded in CDCl_3_ at 50 MHz. ^b^ Recorded in CDCl_3_ at 75 MHz.

**Table 2:** ^1^H NMR data (*δ* in ppm, multiplicity, *J* in Hz) of compounds **1**–**4**.

No.	1 ^a^		2 ^a^		3 ^b^		4 ^a^	
1	2.88	br s	2.90	br d 4.1	2.13	br s	3.58	ddd 7.5, 7.5, 1.4
2	1.34	m	α 1.48 β 1.34	m m	α 1.89 β 2.20	br d 14.4 br d 14.4	α 1.60 β 2.10	m m
3	1.17	m	1.14	m	–		1.61	m
5	α 1.73 β 1.31	m ddd 14.2, 4.0, 3.8	α 1.76 β 1.36	m m	α 2.60 β 1.60	ddd 13.9, 13.8, 4.1 dm 13.9	α 1.62 β 0.92	ddd 13.2, 13.2, 4.4 m
6	α 1.86 β 1.38	ddd 12.9, 4.0, 2.2 ddd 12.9, 12.9, 3.8	α 1.88 β 1.40	ddd 13.2, 4.7, 2.3 ddd 13.2, 13.2, 3.2	α 1.92 β 1.17	ddd 13.2, 4.2, 3.0 m	α 1.90 β 1.34	ddd 13.2, 4.4, 2.9 ddd 13.2, 13.2, 4.0
8	4.09	dd 12.6, 4.0	4.07	dd 12.6, 4.1	3.97	dd 12.6, 4.2	4.04	dd 12.8, 4.0
9	α 2.48 β 2.08	dddd 13.4, 13.4, 12.6, 4.6 dddd 13.4, 4.6, 4.0, 3.0	α 2.47 β 2.06	dddd 13.4, 13.4, 12.6, 4.7 dddd 13.4, 4.7, 4.1, 2.9	α 2.48 β 2.04	dddd 13.8, 13.8, 12.6, 4.8 dddd 13.8, 4.2, 4.2, 3.0	α 2.49 β 2.05	dddd 13.4, 13.4, 12.8, 4.4 m
10	α 1.59 β 1.68	ddd 14.5, 4.6, 3.0 ddd 14.5, 13.4, 4.6	α 1.58 β 1.66	ddd 14.3, 4.7, 2.9 ddd 14.3, 13.4, 4.7	α 1.58 β 1.54	m m	α 1.67 β 1.54	ddd 13.4, 4.4, 2.9 m
12	1.97	d 12.1	1.93	d 12.0	1.07	d 11.0	1.49	d 9.9
13	1.81	br d 12.1	1.74	m	1.77	ddd 11.0, 8.4, 4.8	2.02	m
14	3.98	dd 8.6, 5.6	4.03	dd 8.5, 5.0	α 1.66 β 1.55	ddd 12.0, 8.4, 2.4 m	2.27	dd 7.3, 1.4
15	1.56	s	1.49	s	1.14	s	1.10	s
16	1.16	s	1.16	s	1.18	s	1.05	s
17	a 2.60 b 1.75	dd 14.2, 5.6 dd 14.2, 8.6	a 2.52 b 1.75	dd 14.3, 5.0 dd 14.3, 8.5	a 1.83 b 1.01	br d 9.6 br d 9.6	a 2.44 b 0.61	dd 9.5, 7.3 dd 9.5, 5.4
18	1.72	m	1.71	m	–		2.04	m
19	0.85	d 6.7	0.86	d 6.4	1.84	br s	0.91	d 6.9
20	0.84	d 6.7	0.85	d 6.4	1.57	br s	0.89	d 6.9
OMe	–		–		–		3.30	s

^a^ Recorded in CDCl_3_ at 400 MHz; ^b^ Recorded in CDCl_3_ at 600 MHz.

**Table 3:** ^1^H NMR data (*δ* in ppm, multiplicity, *J* in Hz) of compounds **5**–**8**.

No.	5 ^a^		6 ^a^		7 ^a^		8 ^a^	
1	α 2.28 β 1.82	m m	a 2.23 b 1.75	m dddd 12.6, 12.6, 10.5, 5.6	5.55	dm 10.2	5.69	dm 10.5
2	a 1.85 b 1.72	m m	α 1.25 β 1.63	m dddd 12.6, 12.0, 6.2, 5.0	α 1.93 β 2.02	m m	α 1.98 β 2.10	m m
3	1.76	m	2.01	ddd 12.0, 10.2, 4.3	1.57	ddd 10.5, 7.3, 3.2	1.74	m
5	a 2.09 b 1.84	dt 16.6, 4.6 m	5.27	dd 12.0, 6.2	α 1.49 β 1.29	m ddd 14.3, 14.0, 2.9	α 1.73 β 1.50	ddd 14.0, 14.0, 4.0 ddd 14.0, 4.7, 2.9
6	1.73	m	α 2.23 β 1.93	dd 14.0, 6.2 dd 14.0, 12.0	α 1.49 β 1.84	m ddd 14.0, 3.5, 2.9	α 0.97 β 2.13	ddd 14.0, 4.0, 2.9 ddd 14.0, 14.0, 4.7
8	6.81	d 10.2	4.00	dd 12.6, 4.1	4.55	dd 12.9, 4.7	3.09	br d 6.9
9	5.92	d 10.2	α 2.53 β 2.11	dddd 13.8, 13.8, 12.6, 4.4 dddd 13.8, 4.7, 4.1, 2.6	α 2.53 β 2.08	dddd 13.1, 13.1, 12.9, 4.4 dddd 13.1, 4.7, 4.4, 3.8	α 2.81 β 2.67	dd 18.4, 6.9 br d 18.4
10	–		α 1.69 β 1.46	ddd 14.3, 4.4, 2.6 ddd 14.3, 13.8, 4.7	α 1.45 β 1.71	ddd 14.0, 4.4, 3.8 ddd 14.0, 13.1, 4.4	–	
12	2.13	d 10.0	1.79	d 9.6	1.74	dd 12.3, 1.8	2.46	d 12.9
13	5.60	ddd 15.8, 10.0, 1.1	5.27	dd 14.9, 9.6	1.93	dm 12.3	2.71	dm 12.9
14	5.72	dt 15.8, 6.7	5.18	ddd 14.9, 10.5, 2.2	5.80	dm 10.2	5.95	br d 10.5
15	1.20	s	1.06	s	1.33	s	1.29	s
16	1.27	s	1.28	s	1.34	s	0.76	s
17	a 4.87 b 4.76	br s br s	1.52	br s	0.77	s	a 3.93 b 3.70	d 10.5 dd 10.5, 1.8
18	1.42	br hept 6.6	1.43	dhept 10.2, 6.7	2.13	dhept 7.0, 3.2	1.94	dhept 6.7, 2.0
19	0.85	d 6.6	0.90	d 6.7	0.86	d 7.0	0.87	d 6.7
20	0.76	d 6.6	0.67	d 6.7	0.78	d 7.0	0.93	d 6.7
OMe	–		–		–		3.36	s
11OH	–		–		–		3.48	s

^a^ Recorded in CDCl_3_ at 400 MHz.

**Table 4:** In vitro growth inhibitory activity (IC_50_ values in μM) of compounds **1**–**8** and doxorubicin used as positive control.

	Human Cancer Cell Line					Murine Cancer Cell Line	
Compound	A549	Hs683	MCF7	SKMEL28	U373	B16F10	Mean ± SEM
**1**	84	66	50	>100	98	69	>78
**2**	55	50	48	82	86	68	65 ± 7
**3**	48	40	43	62	81	56	55 ± 7
**4**	68	52	46	>100	>100	49	>69
**5**	20	18	9	23	11	8	15 ± 3
**6**	42	40	41	35	32	58	41 ± 4
**7**	42	35	45	72	65	27	48 ± 8
**8**	17	15	10	25	13	16	16 ± 2
Doxorubicin	0.45	0.36	0.16	0.44	0.33	n.d.^a^	0.35 ± 0.03

^a^ Not determined.

## Supplementary Materials

The following are available online at https://www.mdpi.com/1660-3397/18/1/29/s1, Tables S1–S8: detailed NMR data of compounds **1**–**8**; Figures S1–S72: 1D and 2D NMR, HR-MS and IR spectra of compounds **1**–**8**.
