# Supplementary material for: Halogenated Diterpenes with In Vitro Antitumor Activity from the Red Alga Sphaerococcus coronopifolius
Source: Mar Drugs. 2019 Dec 29;18(1):29. doi: 10.3390/md18010029 (PMC7024270; doi:10.3390/md18010029)

Supplementary Materials for

Halogenated Diterpenes with *in vitro* Anti-Tumor Activity from the Red Alga *Sphaerococcus coronopifolius*

Vangelis Smyrniotopoulos [1], Anna Cláudia de Andrade Tomaz [1,2,3], Maria de Fátima Vanderlei de Souza [2,4], Emídio Vasconcelos Leitão da Cunha [2,5], Robert Kiss [6], Véronique Mathieu [7,8], Efstathia Ioannou [1] and Vassilios Roussis [1,*]

1 Section of Pharmacognosy and Chemistry of Natural Products, Department of Pharmacy, National and Kapodistrian University of Athens, Panepistimiopolis Zografou, 15771 Athens, Greece; esmiriniot@pharm.uoa.gr (V.S.); annacatomaz@gmail.com (A.C.A.T.); eioannou@pharm.uoa.gr (E.I.)

2 Postgraduate Program in Bioactive Natural and Synthetic Products, Health Sciences Center, Federal University of Paraíba, João Pessoa 58051-970, PB, Brazil; mfvanderlei@ltf.ufpb.br (M.F.V.S.); emidiovlcunha@gmail.com (E.V.L.C.)

3 Present address: Hospital Universitário Professor Alberto Antunes, Maceió 57072-900, AL, Brazil

4 Present address: Department of Pharmaceutical Sciences, Federal University of Paraíba, João Pessoa 58051-900, PB, Brazil

5 Present address: Department of Pharmacy, Center of Health and Biological Sciences, State University of Paraiba, Campina Grande 58100-000, PB, Brazil

6 Retired – previously at the Fonds National de la Recherche Scientifique, Belgium; rkiss2012@gmail.com (R.K.)

7 Department of Pharmacotherapy and Pharmaceutics, Université Libre de Bruxelles, Boulevard du Triomphe, 1050 Brussels, Belgium; vemathie@ulb.ac.be (V.M.)

8 ULB Cancer Research Center, Université Libre de Bruxelles, Boulevard du Triomphe, 1050 Brussels, Belgium

* Correspondence: roussis@pharm.uoa.gr; Tel.: +30-210-727-4592

Table of Contents

Table S1. ^{1}H (400 MHz) and ^{13}C (50 MHz) NMR chemical shifts ($CDCl_3$), NOESY and HMBC correlations of iodocoronol (**1**). 1

Table S2. ^{1}H (400 MHz) and ^{13}C (50 MHz) NMR chemical shifts ($CDCl_3$), NOESY and HMBC correlations of bromocoronol (**2**). 2

Table S3. ^{1}H (600 MHz) and ^{13}C (75 MHz) NMR chemical shifts ($CDCl_3$), NOESY and HMBC correlations of bromotetrasphaereniol (**3**). 3

Table S4. ^{1}H (400 MHz) and ^{13}C (50 MHz) NMR chemical shifts ($CDCl_3$), NOESY and HMBC correlations of 1-methoxy-ioniol I (**4**). 4

Table S5. ^{1}H (400 MHz) and ^{13}C (50 MHz) NMR chemical shifts ($CDCl_3$), NOESY and HMBC correlations of corotrienone (**5**). 5

Table S6. ^{1}H (400 MHz) and ^{13}C (50 MHz) NMR chemical shifts ($CDCl_3$), NOESY and HMBC correlations of iso-bromocorodienol (**6**). 6

Table S7. ^{1}H (400 MHz) and ^{13}C (50 MHz) NMR chemical shifts ($CDCl_3$), NOESY and HMBC correlations of debromosphaerol (**7**). 7

Table S8. ^{1}H (400 MHz) and ^{13}C (50 MHz) NMR chemical shifts ($CDCl_3$), NOESY and HMBC correlations of 8-methoxy-dihydro-sphaerococcenol (**8**). 8

Figure S1. ^{1}H NMR spectrum (400 MHz, $CDCl_3$) of iodocoronol (**1**). 9

Figure S2. ^{13}C NMR spectrum (50 MHz, $CDCl_3$) of iodocoronol (**1**). 10

Figure S3. COSY spectrum (400 MHz, $CDCl_3$) of iodocoronol (**1**). 11

Figure S4. HSQC-DEPT spectrum (400 MHz, $CDCl_3$) of iodocoronol (**1**). 12

Figure S5. HMBC spectrum (400 MHz, $CDCl_3$) of iodocoronol (**1**). 13

Figure S6. NOESY spectrum (400 MHz, $CDCl_3$) of iodocoronol (**1**). 14

Figure S7. HRMS (ESI-) measurement of iodocoronol (**1**). 15

Figure S8. IR spectrum of iodocoronol (**1**). 16

Figure S9. ^{1}H NMR spectrum (400 MHz, $CDCl_3$) of bromocoronol (**2**). 17

Figure S10. ^{13}C NMR spectrum (50 MHz, $CDCl_3$) of bromocoronol (**2**). 18

Figure S11. DEPT-135 spectrum (50 MHz, $CDCl_3$) of bromocoronol (**2**). 19

Figure S12. COSY spectrum (400 MHz, $CDCl_3$) of bromocoronol (**2**). 20

Figure S13. HSQC spectrum (400 MHz, $CDCl_3$) of bromocoronol (**2**). 21

Figure S14. HSQC-TOCSY spectrum (400 MHz, $CDCl_3$) of bromocoronol (**2**). 22

Figure S15. HMBC spectrum (400 MHz, $CDCl_3$) of bromocoronol (**2**). 23

Figure S16. NOESY spectrum (400 MHz, $CDCl_3$) of bromocoronol (**2**). 24

Figure S17. HRMS (ESI+) measurement of bromocoronol (**2**). 25

Figure S18. IR spectrum of bromocoronol (**2**). 26

Figure S19. ^{1}H NMR spectrum (600 MHz, $CDCl_3$) of bromotetrasphaereniol (**3**). 27

Figure S20. ^{13}C NMR spectrum (75 MHz, $CDCl_3$) of bromotetrasphaereniol (**3**). 28

Figure S21. COSY spectrum (600 MHz, $CDCl_3$) of bromotetrasphaereniol (**3**). 29

Figure S22. HSQC-DEPT spectrum (400 MHz, $CDCl_3$) of bromotetrasphaereniol (**3**). 30

Figure S23. HMBC spectrum (600 MHz, $CDCl_3$) of bromotetrasphaereniol (**3**). 31

Figure S24. NOESY spectrum (600 MHz, $CDCl_3$) of bromotetrasphaereniol (**3**). 32

Figure S25. HRMS (ESI+) measurement of bromotetrasphaereniol (**3**). 33

Figure S26. IR spectrum of bromotetrasphaereniol (**3**). 34

Figure S27. ^{1}H NMR spectrum (400 MHz, $CDCl_3$) of 1-methoxy-ioniol I (**4**). 35

Figure S28. ^{13}C NMR spectrum (50 MHz, $CDCl_3$) of 1-methoxy-ioniol I (**4**). 36

Figure S29. COSY spectrum (400 MHz, $CDCl_3$) of 1-methoxy-ioniol I (**4**). 37

Figure S30. HSQC spectrum (400 MHz, $CDCl_3$) of 1-methoxy-ioniol I (**4**). 38
Figure S31. HMBC spectrum (400 MHz, $CDCl_3$) of 1-methoxy-ioniol I (**4**). 39
Figure S32. NOESY spectrum (400 MHz, $CDCl_3$) of 1-methoxy-ioniol I (**4**). 40
Figure S33. HRMS (ESI+) measurement of 1-methoxy-ioniol I (**4**). 41
Figure S34. IR spectrum of 1-methoxy-ioniol I (**4**). 42
Figure S35. ^{1}H NMR spectrum (400 MHz, $CDCl_3$) of corotrienone (**5**). 43
Figure S36. ^{13}C NMR spectrum (50 MHz, $CDCl_3$) of corotrienone (**5**). 44
Figure S37. COSY spectrum (400 MHz, $CDCl_3$) of corotrienone (**5**). 45
Figure S38. HSQC-DEPT spectrum (400 MHz, $CDCl_3$) of corotrienone (**5**). 46
Figure S39. HMBC spectrum (400 MHz, $CDCl_3$) of corotrienone (**5**). 47
Figure S40. NOESY spectrum (400 MHz, $CDCl_3$) of corotrienone (**5**). 48
Figure S41. HRMS (ESI+) measurement of corotrienone (**5**). 49
Figure S42. IR spectrum of corotrienone (**5**). 50
Figure S42. ^{1}H NMR spectrum (400 MHz, $CDCl_3$) of iso-bromocorodienol (**6**). 51
Figure S43. ^{13}C NMR spectrum (50 MHz, $CDCl_3$) of iso-bromocorodienol (**6**). 52
Figure S44. COSY spectrum (400 MHz, $CDCl_3$) of iso-bromocorodienol (**6**). 53
Figure S45. HSQC-DEPT spectrum (400 MHz, $CDCl_3$) of iso-bromocorodienol (**6**). 54
Figure S47. NOESY spectrum (400 MHz, $CDCl_3$) of iso-bromocorodienol (**6**). 56
Figure S48. 1D NOE spectrum (400 MHz, $CDCl_3$), excitation of H-6β of iso-bromocorodienol (**6**). 57
Figure S49. HRMS (ESI+) measurement of iso-bromocorodienol (**6**). 58
Figure S50. IR spectrum of iso-bromocorodienol (**6**). 59
Figure S51. ^{1}H NMR spectrum (400 MHz, $CDCl_3$) of debromosphaerol (**7**). 60
Figure S52. ^{13}C NMR spectrum (50 MHz, $CDCl_3$) of debromosphaerol (**7**). 61
Figure S53. DEPT-135 spectrum (50 MHz, $CDCl_3$) of debromosphaerol (**7**). 62
Figure S54. COSY spectrum (400 MHz, $CDCl_3$) of debromosphaerol (**7**). 63
Figure S55. HSQC-DEPT spectrum (400 MHz, $CDCl_3$) of debromosphaerol (**7**). 64
Figure S56. HMBC spectrum (400 MHz, $CDCl_3$) of debromosphaerol (**7**). 65
Figure S57. NOESY spectrum (400 MHz, $CDCl_3$) of debromosphaerol (**7**). 66
Figure S58. 1D NOE spectrum (400 MHz, $CDCl_3$), excitation of H-3 of debromosphaerol (**7**). 67
Figure S59. 1D NOE spectrum (400 MHz, $CDCl_3$), excitation of H-12 of debromosphaerol (**7**). 68
Figure S60. 1D NOE spectrum (400 MHz, $CDCl_3$), excitation of H-13 and H-2α of debromosphaerol (**7**). 69
Figure S61. HRMS (ESI+) measurement of debromosphaerol (**7**). 70
Figure S62. IR spectrum of debromosphaerol (**7**). 71
Figure S63. ^{1}H NMR spectrum (400 MHz, $CDCl_3$) of 8-methoxy-dihydro-sphaerococcenol (**8**). 72
Figure S64. ^{13}C NMR spectrum (50 MHz, $CDCl_3$) of 8-methoxy-dihydro-sphaerococcenol (**8**). 73
Figure S65. DEPT-135 spectrum (50 MHz, $CDCl_3$) of 8-methoxy-dihydro-sphaerococcenol (**8**). 74
Figure S66. COSY spectrum (400 MHz, $CDCl_3$) of 8-methoxy-dihydro-sphaerococcenol (**8**). 75
Figure S67. HSQC spectrum (400 MHz, $CDCl_3$) of 8-methoxy-dihydro-sphaerococcenol (**8**). 76
Figure S68. HMBC spectrum (400 MHz, $CDCl_3$) of 8-methoxy-dihydro-sphaerococcenol (**8**). 77
Figure S69. NOESY spectrum (400 MHz, $CDCl_3$) of 8-methoxy-dihydro-sphaerococcenol (**8**). 78
Figure S70. Chair conformation of 8-methoxy-dihydro-sphaerococcenol (**8**), energy: 59.57 Kcal/mole. 79
Figure S71. HRMS (ESI+) measurement of 8-methoxy-dihydro-sphaerococcenol (**8**). 80
Figure S72. IR spectrum of 8-methoxy-dihydro-sphaerococcenol (**8**). 81

Table S1. ^{1}H (400 MHz) and ^{13}C (50 MHz) NMR chemical shifts ($CDCl_3$), NOESY and HMBC correlations of iodocoronol (**1**).

No.	^{1}H (δ)	m (J)	NOESY	^{13}C (δ)	Type	HMBC ($^{13}C \rightarrow {}^{1}H$)
1	2.88	br. s	2, 13, 14, 15	49.4	CH	2, 17a
2	1.34	m	1, 14, 20	35.6	CH_2	3, 14
3	1.17	m	14, 17b	48.8	CH	1, 17a, 17b, 19, 20
4	-			51.9	C	1, 2, 3, 5α, 13, 17b
5	α 1.73	m	16	22.8	CH_2	3
	β 1.31	ddd 14.2, 4.0, 3.8				
6	α 1.86	ddd 12.9, 4.0, 2.2		37.8	CH_2	12, 16
	β 1.38	ddd 12.9, 12.9, 3.8	8, 12, 17a			
7	-			41.5	C	16
8	4.09	dd 12.6, 4.0	6β, 9β. 10β, 12	68.5	CH	16
9	α 2.48	dddd 13.4, 13.4, 12.6, 4.6	16	31.0	CH_2	10b
	β 2.08	dddd 13.4, 4.6, 4.0, 3.0	8, 10β			
10	α 1.59	ddd 14.5, 4.6, 3.0		43.6	CH_2	15
	β 1.68	ddd 14.5, 13.4, 4.6	8, 9β, 12			
11	-			73.6	C	15
12	1.97	d 12.1	6β, 8, 10β, 15, 17a	47.6	CH	15, 16
13	1.81	br. d 12.1	1, 15, 16, 19	44.8	CH	2, 5, 12, 14, 17b
14	3.98	dd 8.6, 5.6	1, 2, 3, 17b	25.9	CH	1, 2, 13, 17a
15	1.56	s	1, 12, 13	32.9	CH_3	
16	1.16	s	5α, 9α, 13	16.6	CH_3	12
17	a 2.60	dd 14.2, 5.6	6β, 12	50.2	CH_2	3, 5α, 13
	b 1.75	dd 14.2, 8.6	3, 14			
18	1.72	m		28.3	CH	2, 3, 19, 20
19	0.85	d 6.7	13	23.4	CH_3	3, 20
20	0.84	d 6.7	2	18.7	CH_3	3, 19

Table S2. ^{1}H (400 MHz) and ^{13}C (50 MHz) NMR chemical shifts ($CDCl_3$), NOESY and HMBC correlations of bromocoronol (**2**).

No.	^{1}H (δ)	m (J)	NOESY	^{13}C (δ)	Type	HMBC ($^{13}C \rightarrow {}^{1}H$)
1	2.90	br d 4.1	2α, 2β, 13, 14, 15	49.3	CH	2α, 13
2	α 1.48	m	1, 13	34.0	CH_2	3, 14, 18
	β 1.34	m	1, 14			
3	1.14	m	14, 17b	48.9	CH	2β, 5α, 17a, 17b, 19, 20
4	–			51.3	C	1, 3, 6α, 13, 17a
5	α 1.76	m	16	22.9	CH_2	6α
	β 1.36	m	17a			
6	α 1.88	ddd 13.2, 4.7, 2.3	16	37.7	CH_2	5β, 16
	β 1.40	ddd 13.2, 13.2, 3.2	8, 12, 17a			
7	–			41.4	C	6α, 12, 16
8	4.07	dd 12.6, 4.1	6β, 9β, 10β, 12	68.6	CH	9α, 10α, 10β, 12, 16
9	α 2.47	dddd 13.4, 13.4, 12.6, 4.7	16	30.9	CH_2	10α, 10β
	β 2.06	dddd 13.4, 4.7, 4.1, 2.9	8, 10β			
10	α 1.58	ddd 14.3, 4.7, 2.9		43.7	CH_2	15
	β 1.66	ddd 14.3, 13.4, 4.7	8, 9β,12			
11	–			73.5	C	15
12	1.93	d 12.0	6β, 8, 10β, 15, 17a	47.9	CH	15, 16
13	1.74	m	1, 2α, 16	44.5	CH	2β, 3, 5α, 14, 17b
14	4.03	dd 8.5, 5.0	1, 2β, 3, 17b	52.5	CH	1, 2α, 2β, 17a, 17b
15	1.49	s	1, 12	33.2	CH_3	
16	1.16	s	5α, 6α, 9α, 13	16.3	CH_3	12
17	a 2.52	dd 14.3, 5.0	5β, 6β, 12	48.6	CH_2	1
	b 1.75	dd 14.3, 8.5	3, 14			
18	1.71	m		28.3	CH	3, 19, 20
19	0.86	d 6.4		23.4	CH_3	3, 20
20	0.85	d 6.4		18.8	CH_3	3, 18, 19

Table S3. ^{1}H (600 MHz) and ^{13}C (75 MHz) NMR chemical shifts ($CDCl_3$), NOESY and HMBC correlations of bromotetrasphaereniol (**3**).

No.	^{1}H (δ)	m (J)	NOESY	^{13}C (δ)	Type	HMBC ($^{13}C \rightarrow {}^{1}H$)
1	2.13	br. s	2α, 14α, 14β, 17a, 17b	34.7	CH	2α, 14α, 17a, 17b
2	α 1.89	br. d 14.4	1, 14α, 20	41.0	CH_2	14α, 17a, 17b
	β 2.20	br. d 14.4	17b, 20			
3	–			139.0	C	1, 5α, 13, 17b, 19, 20
4	–			52.8	C	1, 5α, 5β, 6α, 14α, 17a, 17b
5	α 2.60	ddd 13.9, 13.8, 4.1	6α, 16, 19	24.4	CH_2	6β
	β 1.60	dm 13.9	6α, 6β, 17b			
6	α 1.92	ddd 13.2, 4.2, 3.0	5α, 5β, 16	38.7	CH_2	5α, 8, 16
	β 1.17	m	5β, 8, 12, 17a			
7	–			41.0	C	5β, 8, 12, 16
8	3.97	dd 12.6, 4.2	6β, 9β, 10β, 12	68.5	CH	9α, 10α, 10β, 12, 16
9	α 2.48	dddd 13.8, 13.8, 12.6, 4.8	16	30.9	CH_2	8, 10β
	β 2.04	dddd 13.8, 4.2, 4.2, 3.0	8			
10	α 1.58	m	15	43.3	CH_2	9α, 15
	β 1.54	m	8, 12, 15			
11	–			72.9	C	10β, 15
12	1.07	d 11.0	6β, 8, 10β, 14β, 15, 17a	56.4	CH	6α, 10α, 13, 14α, 15, 16
13	1.77	ddd 11.0, 8.4, 4.8	15, 16	39.0	CH	1, 5β, 12, 17b
14	α 1.66	ddd 12.0, 8.4, 2.4	1, 2α, 15	43.1	CH_2	2α, 2β, 12, 13, 17b
	β 1.55	m	1, 12, 15, 17a			
15	1.14	s	10α, 10β, 12, 13, 14α, 14β	32.7	CH_3	
16	1.18	s	5α, 6α, 9α, 13, 19	16.9	CH_3	8, 12
17	a 1.83	br. d 9.6	1, 6β, 12, 14β	43.9	CH_2	2α, 5α, 14α
	b 1.01	br. d 9.6	1, 2β, 5β			
18	–			119.9	C	19, 20
19	1.84	br. s	5α, 16, 20	20.3	CH_3	20
20	1.57	br. s	2α, 2β, 19	23.9	CH_3	19

Table S4. ^{1}H (400 MHz) and ^{13}C (50 MHz) NMR chemical shifts ($CDCl_3$), NOESY and HMBC correlations of 1-methoxy-ioniol I (**4**).

No.	^{1}H (δ)	m (*J*)	NOESY	^{13}C (δ)	Type	HMBC
1	3.58	ddd 7.5, 7.5, 1.4	2β, 14, 17b	81.9	CH	2α, 2β, 3, 14, 17a, 21
2	α 1.60	m		25.1	CH_2	14
	β 2.10	m	1			
3	1.61	m	17b	48.3	CH	17a, 19, 20
4	–			43.5	C	2, 6α, 14, 17a
5	α 1.62	ddd 13.2, 13.2, 4.4	16	24.0	CH_2	17a
	β 0.92	m	6β			
6	α 1.90	ddd 13.2, 4.4, 2.9	16	37.0	CH_2	12, 16
	β 1.34	ddd 13.2, 13.2, 4.0	5β, 8, 17a			
7	–			39.3	C	5α, 12, 16
8	4.04	dd 12.8, 4.0	6β, 9β, 10β, 12	68.7	CH	12, 16
9	α 2.49	dddd 13.4, 13.4, 12.8, 4.4	16	30.6	CH_2	
	β 2.05	m	8			
10	α 1.67	ddd 13.4, 4.4, 2.9		42.5	CH_2	15
	β 1.54	m	8			
11	–			72.6	C	15
12	1.49	d 9.9	8, 14, 15, 17a	52.0	CH	6β, 10α, 15, 16
13	2.02	m	16	31.8	CH	1, 12, 17b
14	2.27	dd 7.3, 1.4	1, 12, 15	40.9	CH	2β, 12, 17a
15	1.10	s	12, 14	30.3	CH_3	
16	1.05	s	5α, 6α, 9α, 13	16.0	CH_3	12
17	a 2.44	dd 9.5, 7.3	6β, 12	34.2	CH_2	13
	b 0.61	dd 9.5, 5.4	1, 3			
18	2.04	m		27.6	CH	19, 20
19	0.91	d 6.9		15.8	CH_3	20
20	0.89	d 6.9		22.6	CH_3	19
21	3.30	s		55.9	OCH_3	1

Table S5. ^1H (400 MHz) and ^{13}C (50 MHz) NMR chemical shifts ($CDCl_3$), NOESY and HMBC correlations of corotrienone (**5**).

No.	^1H (δ)	m (J)	NOESY	^{13}C (δ)	Type	HMBC ($^{13}C \rightarrow {}^1H$)
1	α 2.28	m	13	28.1	CH_2	2, 13, 14
	β 1.82	m	14			
2	a 1.85	m	19	32.2	CH_2	18
	b 1.72	m	13			
3	1.76	m	17a	55.8	CH	1α, 17a, 17b, 19, 20
4	-		-	153.3	C	3, 5b, 6
5	a 2.09	dt 16.6, 4.6	13, 16, 18	25.0	CH_2	6, 17a, 17b
	b 1.84	m				
6	1.73	m		39.3	CH_2	5b, 8, 12, 16
7	-		-	41.4	C	9, 12, 16
8	6.81	d 10.2	16	164.8	CH	16
9	5.92	d 10.2		124.6	CH	
10	-		-	200.6	C	8, 15
11	-		-	73.5	C	9, 15
12	2.13	d 10.0	14, 15, 17b	58.5	CH	6, 8, 14, 15, 16
13	5.60	ddd 15.8, 10.0, 1.1	1α, 2b, 5a, 16	124.2	CH	12, 14
14	5.72	dt 15.8, 6.7	1β, 12, 15, 17a	136.1	CH	1α, 12
15	1.20	s	12, 14	25.2	CH_3	
16	1.27	s	5a, 8, 13	20.4	CH_3	12
17	a 4.87	br s	3, 14, 20	112.3	CH_2	3, 5a
	b 4.76	br s	12			
18	1.42	br. hept 6.6	5a	29.9	CH	3, 19, 20
19	0.85	d 6.6	2a	20.7	CH_3	20
20	0.76	d 6.6	17a	21.5	CH_3	19

Table S6. ^{1}H (400 MHz) and ^{13}C (50 MHz) NMR chemical shifts ($CDCl_3$), NOESY and HMBC correlations of iso-bromocorodienol (**6**).

No.	^{1}H (δ)	m (J)	NOESY	^{13}C (δ)	Type	HMBC ($^{13}C \rightarrow ^{1}H$)
1	a 2.23	m	14	29.7	CH_2	2α, 13
	b 1.75	dddd 12.6, 12.6, 10.5, 5.6	13			
2	α 1.25	m	17, 18	24.8	CH_2	1a, 1b
	β 1.63	dddd 12.6, 12.0, 6.2, 5.0	3, 14			
3	2.01	ddd 12.0, 10.2, 4.3	2β, 6β, 14, 19, 20	44.4	CH	1b, 17, 18, 19, 20
4	–		–	133.3	C	2β, 6α, 6β, 17
5	5.27	dd12.0, 6.2	6α, 16, 17	125.7	CH	3, 6α, 6β, 17
6	α 2.23	dd 14.0, 6.2	5, 16	40.1	CH_2	12, 16
	β 1.93	dd 14.0, 12.0	3, 8, 12, 20			
7	–		–	44.7	C	6α, 6β, 12, 16
8	4.00	dd 12.6, 4.1	6β, 9β, 10β, 12	68.0	CH	9α, 12, 16
9	α 2.53	dddd 13.8, 13.8, 12.6, 4.4	10α, 16	31.1	CH_2	10β
	β 2.11	dddd 13.8, 4.7, 4.1, 2.6	8, 10α, 10β			
10	α 1.69	ddd 14.3, 4.4, 2.6	9α, 9β, 15	40.8	CH_2	9α, 15
	β 1.46	ddd 14.3, 13.8, 4.7	8, 9β, 15			
11	–		–	71.7	C	15
12	1.79	d 9.6	6β, 8, 14, 15	62.3	CH	6β, 10α, 14, 15, 16
13	5.27	dd 14.9, 9.6	1b, 16	128.1	CH	1b, 12
14	5.18	ddd 14.9, 10.5, 2.2	1a, 2β, 3, 12	133.0	CH	2β, 12, 13
15	1.06	s	10α, 10β, 12	30.9	CH_3	
16	1.28	s	5, 6α, 9α, 13	15.2	CH_3	12
17	1.52	br. s	2α, 5	19.1	CH_3	
18	1.43	d hept 10.2, 6.7	2α, 19, 20	30.4	CH	19, 20
19	0.90	d 6.7	3, 18	21.1	CH_3	20
20	0.67	d 6.7	3, 6β, 18	21.3	CH_3	19

Table S7. ^{1}H (400 MHz) and ^{13}C (50 MHz) NMR chemical shifts ($CDCl_3$), NOESY and HMBC correlations of debromosphaerol (**7**).

No.	^{1}H (δ)	m (J)	NOESY	^{13}C (δ)	Type	HMBC ($^{13}C \rightarrow {}^{1}H$)
1	5.55	dm 10.2	2α, 2β	127.0	CH	2β
2	α 1.93	m	1, 17, 19, 20	23.3	CH_2	3, 14, 18
	β 2.02	m	1, 3, 19			
3	1.57	ddd 10.5, 7.3, 3.2	2β, 5β, 13, 18, 19	52.0	CH	17, 19, 20
4	-			38.2	C	3, 6α, 12, 14, 17
5	α 1.49	m	17, 18	31.6	CH_2	6β, 17
	β 1.29	ddd 14.3, 14.0, 2.9	3, 8, 13, 18			
6	α 1.49	m	12, 16, 17	34.8	CH_2	16
	β 1.84	ddd 14.0, 3.5, 2.9	8, 16			
7	-			39.5	C	5α, 6β, 12, 16
8	4.55	dd 12.9, 4.7	5β, 6β, 9β, 10β, 13	60.6	CH	6β, 10α, 10β, 12, 16
9	α 2.53	dddd 13.1, 13.1, 12.9, 4.4	16	30.9	CH_2	10β
	β 2.08	dddd 13.1, 4.7, 4.4, 3.8	8, 10β			
10	α 1.45	ddd 14.0, 4.4, 3.8		38.5	CH_2	15
	β 1.71	ddd 14.0, 13.1, 4.4	8, 9β, 13			
11	-			75.7	C	12, 15
12	1.74	dd 12.3, 1.8	6α, 14, 15, 16, 17	53.6	CH	14, 15, 16
13	1.93	dm 12.3	3, 5β, 8, 10β, 15	45.4	CH	1, 3, 5α, 12, 14, 17
14	5.80	dm 10.2	12, 15	131.9	CH	2α, 12
15	1.33	s	12, 13, 14	35.4	CH_3	
16	1.34	s	6α, 6β, 9α, 12	28.1	CH_3	8
17	0.77	s	2α, 5α, 6α, 12	17.7	CH_3	3, 5β
18	2.13	d hept 7.0, 3.2	3, 5α, 5β, 19, 20	26.5	CH	3, 19, 20
19	0.86	d 7.0	2α, 2β, 3, 18	23.4	CH_3	3, 20
20	0.78	d 7.0	2α, 18	16.6	CH_3	3, 19

Table S8. ^{1}H (400 MHz) and ^{13}C (50 MHz) NMR chemical shifts ($CDCl_3$), NOESY and HMBC correlations of 8-methoxy-dihydro-sphaerococcenol (**8**).

No.	^{1}H (δ)	m (J)	NOESY	^{13}C (δ)	Type	HMBC ($^{13}C \rightarrow ^{1}H$)
1	5.69	dm 10.5	2α, 2β	127.7	CH	2α,2β, 3, 13
2	α 1.98	m	1, 20	22.7	CH_2	1, 3, 14, 18
	β 2.10	m	1, 3, 17b			
3	1.74	m	2β	42.0	CH	5α, 5β, 13, 17a, 17b, 18, 19, 20
4	-			40.1	C	2α, 3, 5α, 5β, 6α, 14, 17a, 17b, 18
5	α 1.73	ddd 14.0, 14.0, 4.0	13, 16, 18	24.7	CH_2	6β, 17a, 17b
	β 1.50	ddd 14.0, 4.7, 2.9	6a, 6β			
6	α 0.97	ddd 14.0, 4.0, 2.9	5β, 8, 16	29.1	CH_2	5α, 8, 16
	β 2.13	ddd 14.0, 14.0, 4.7	5β, 12, 17a, 21			
7	-			39.8	C	5α, 5β, 6α, 8, 9β, 12, 16
8	3.09	br d 6.9	6α, 9α, 16, 21	83.8	CH	9β, 12, 16, 21
9	α 2.81	dd 18.4, 6.9	8, 16	38.9	CH_2	8
	β 2.67	d 18.4	15, 21			
10	-			216.9	C	8, 9α, 9β, 15, 11OH
11	-			76.3	C	9α, 12, 15, 11OH
12	2.46	d 12.9	6β, 14, 15, 17a	42.9	CH	8, 14, 15, 16, 11OH
13	2.71	dm 12.9	5α, 16, 19	35.4	CH	1, 3, 5α, 5β, 12, 14
14	5.95	br d 10.5	12, 15, 11OH	129.0	CH	2α, 12, 13
15	1.29	s	9β, 12, 14, 11OH	31.2	CH_3	12
16	0.76	s	5a, 6a, 8, 9α, 13, 11OH	17.2	CH_3	8, 6β, 12
17	a 3.93	d 10.5	6β, 12	40.6	CH_2	3, 5α, 5β
	b 3.70	dd 10.5, 1.8	2β			
18	1.94	dhept 6.7, 2.0	5α	25.8	CH	19, 20
19	0.87	d 6.7	13	19.3	CH_3	3, 18, 20
20	0.93	d 6.7	2α	25.8	CH_3	3, 18, 19
21	3.36	s	6β, 8, 9β	57.5	CH_3	8
11OH	3.48	s	14, 15, 16	-	OH	

Figure S1. ^{1}H NMR spectrum (400 MHz, $CDCl_3$) of iodocoronol (**1**).

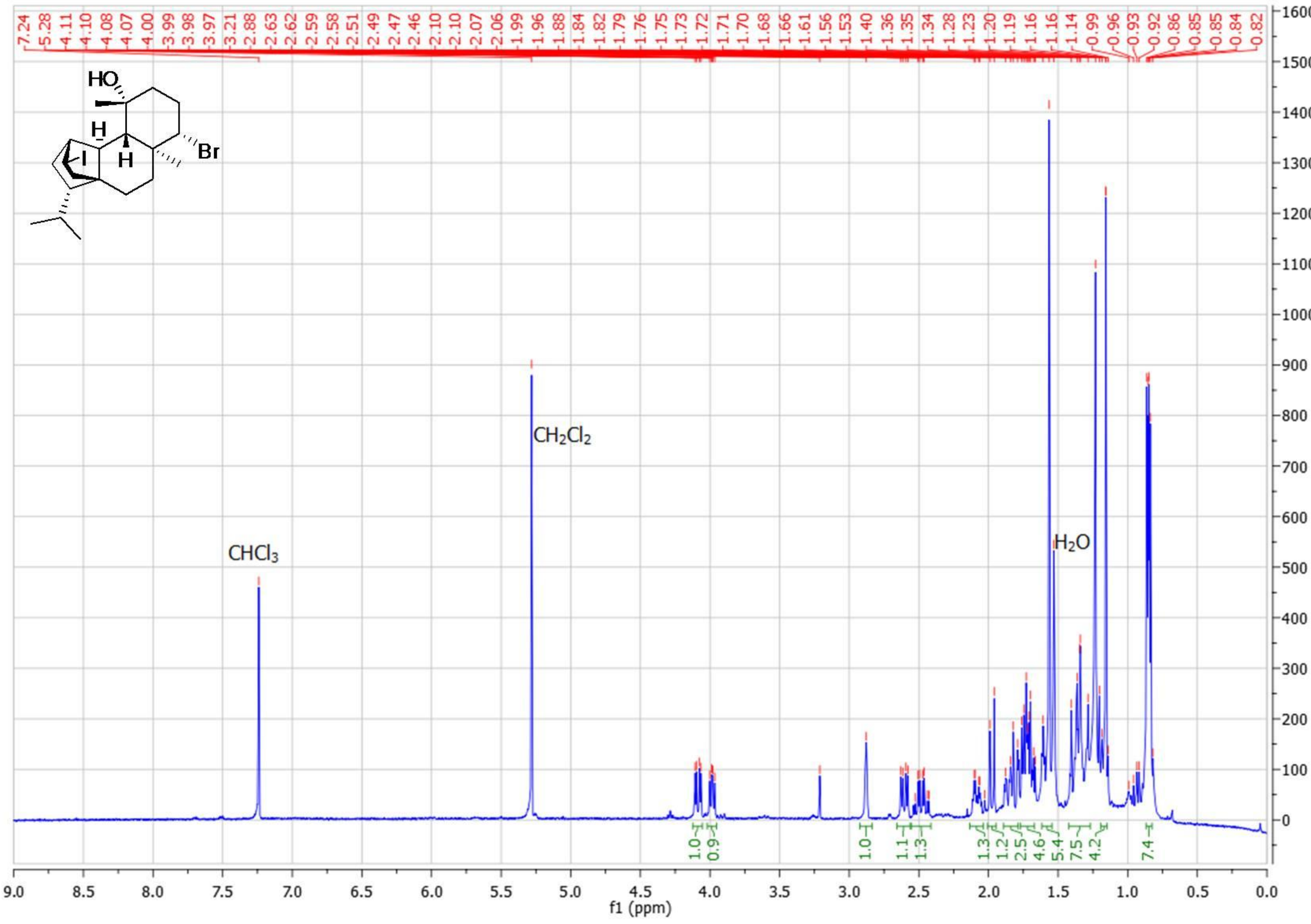

Figure S2. ^{13}C NMR spectrum (50 MHz, $CDCl_3$) of iodocoronol (**1**).

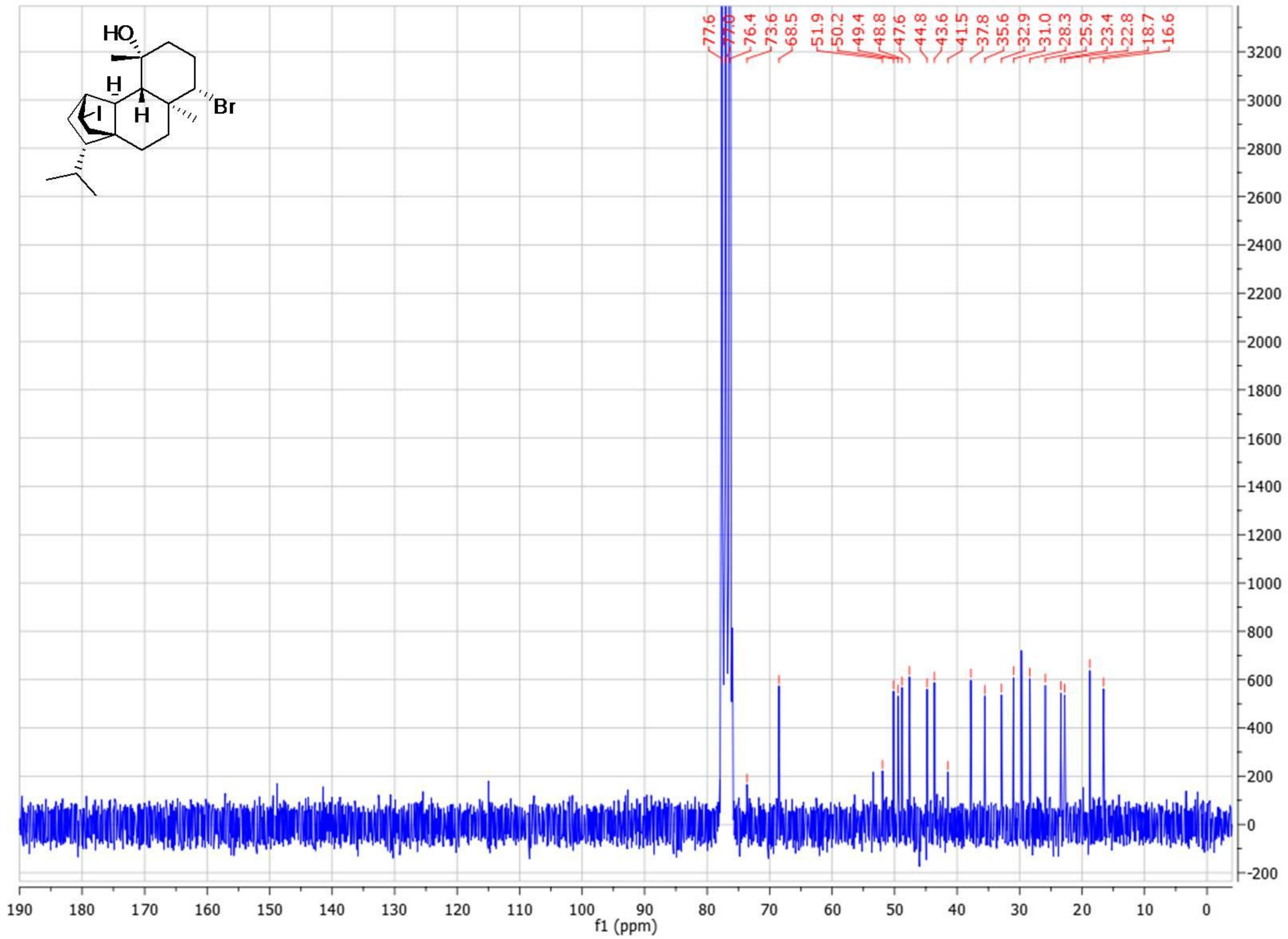

Figure S3. COSY spectrum (400 MHz, $CDCl_3$) of iodocoronol (**1**).

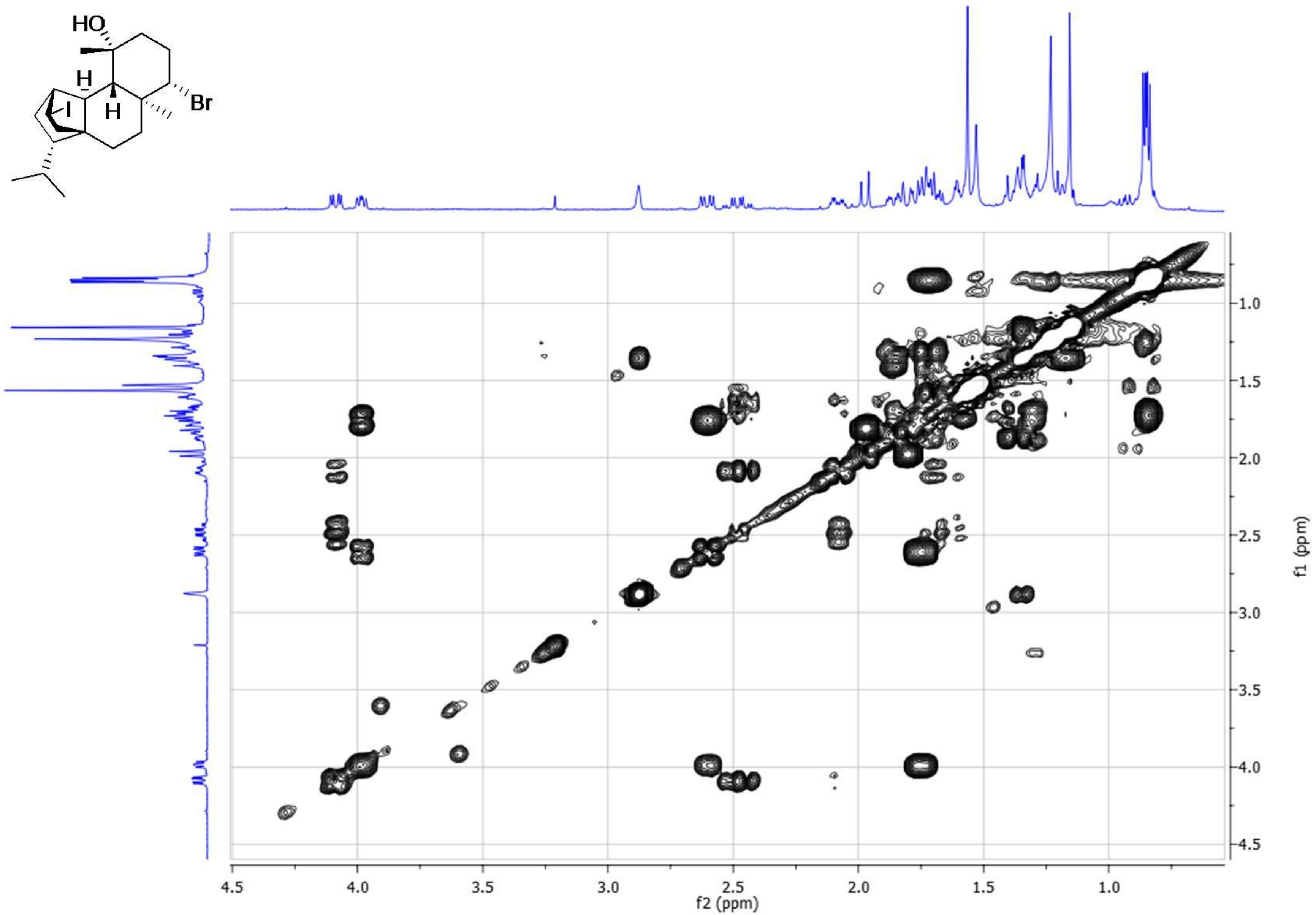

Figure S4. HSQC-DEPT spectrum (400 MHz, $CDCl_3$) of iodocoronol (**1**).

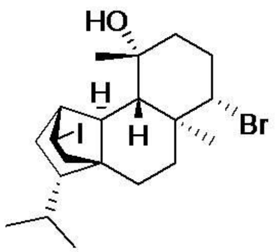

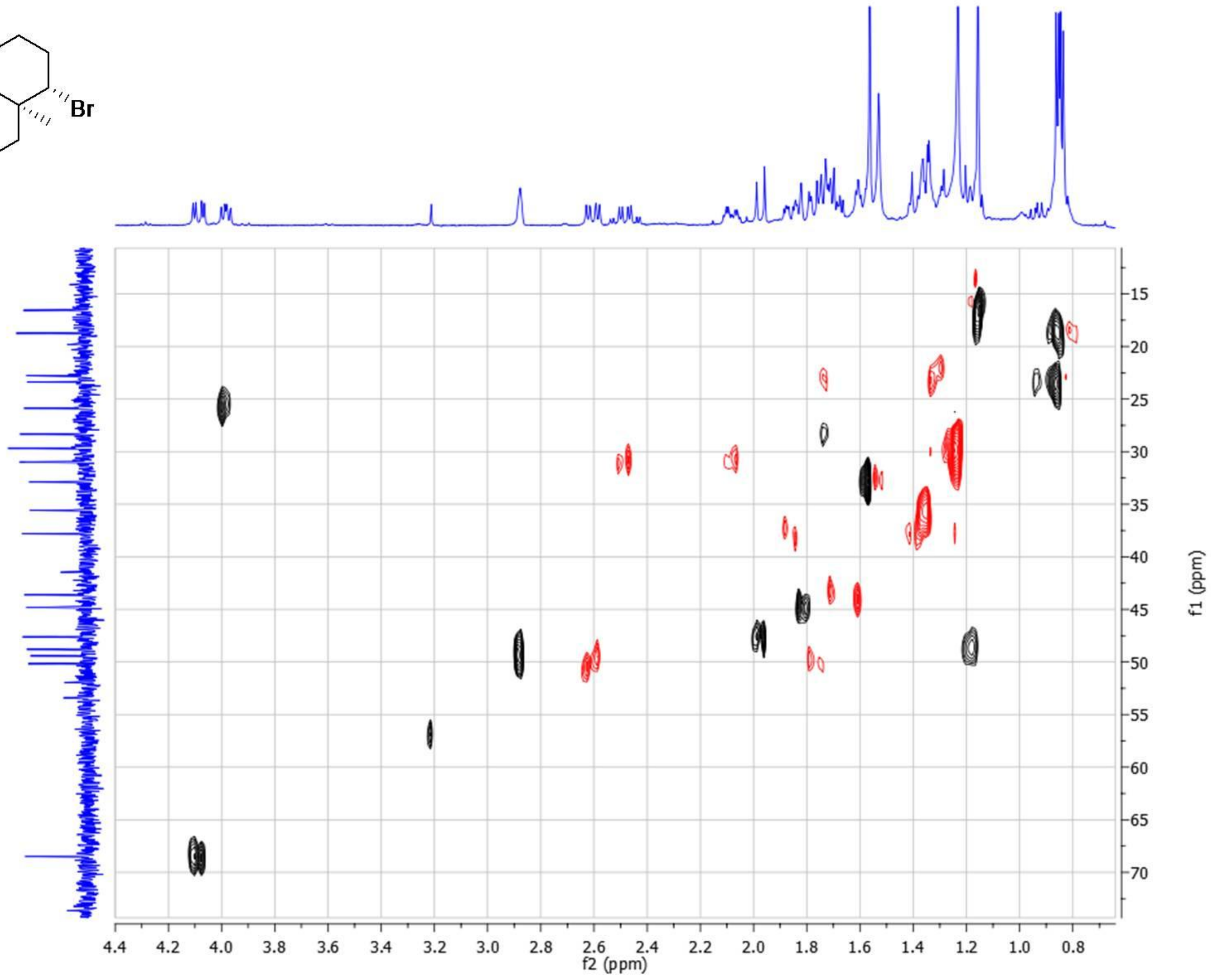

Figure S5. HMBC spectrum (400 MHz, $CDCl_3$) of iodocoronol (**1**).

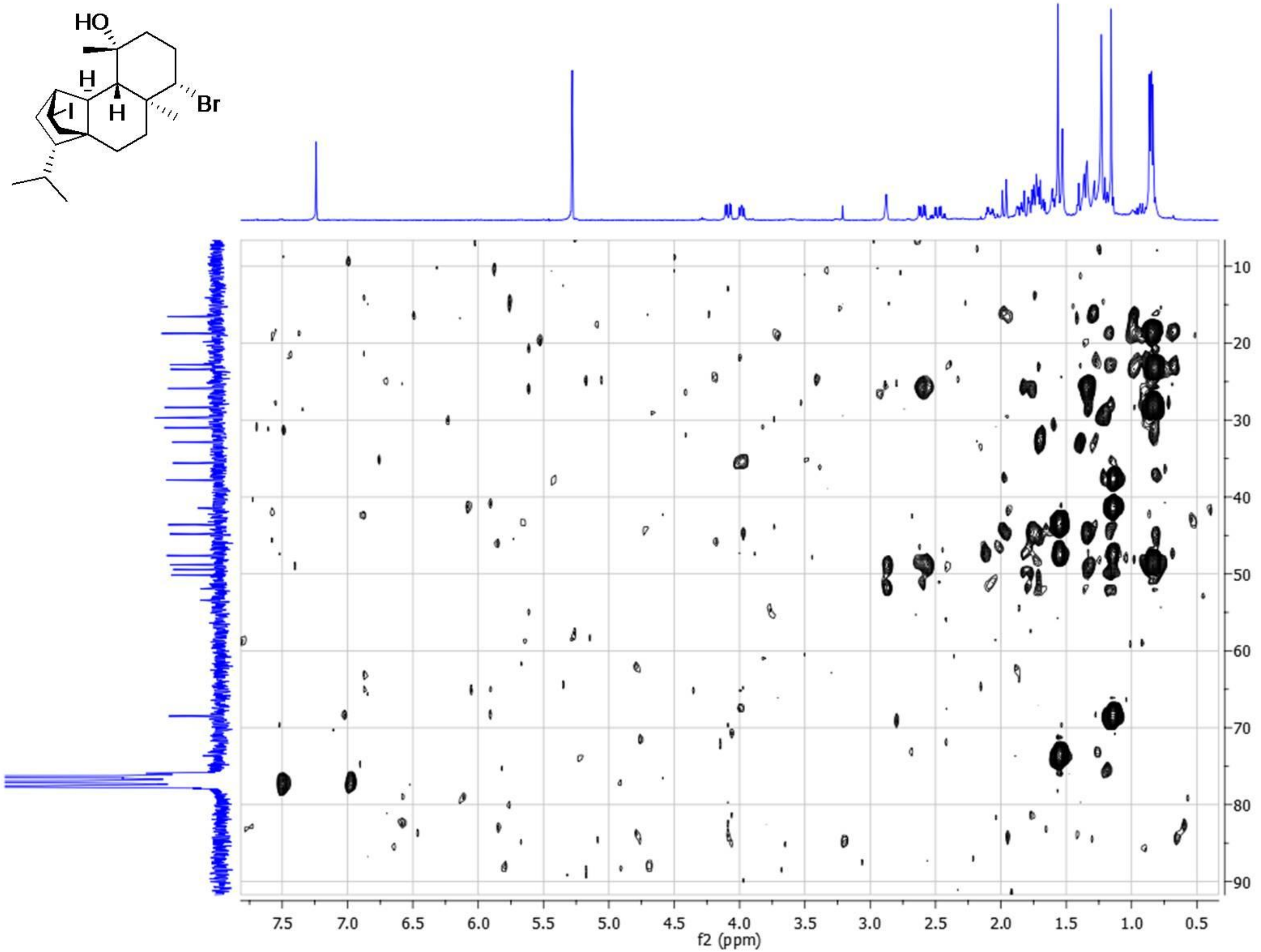

Figure S6. NOESY spectrum (400 MHz, $CDCl_3$) of iodocoronol (**1**).

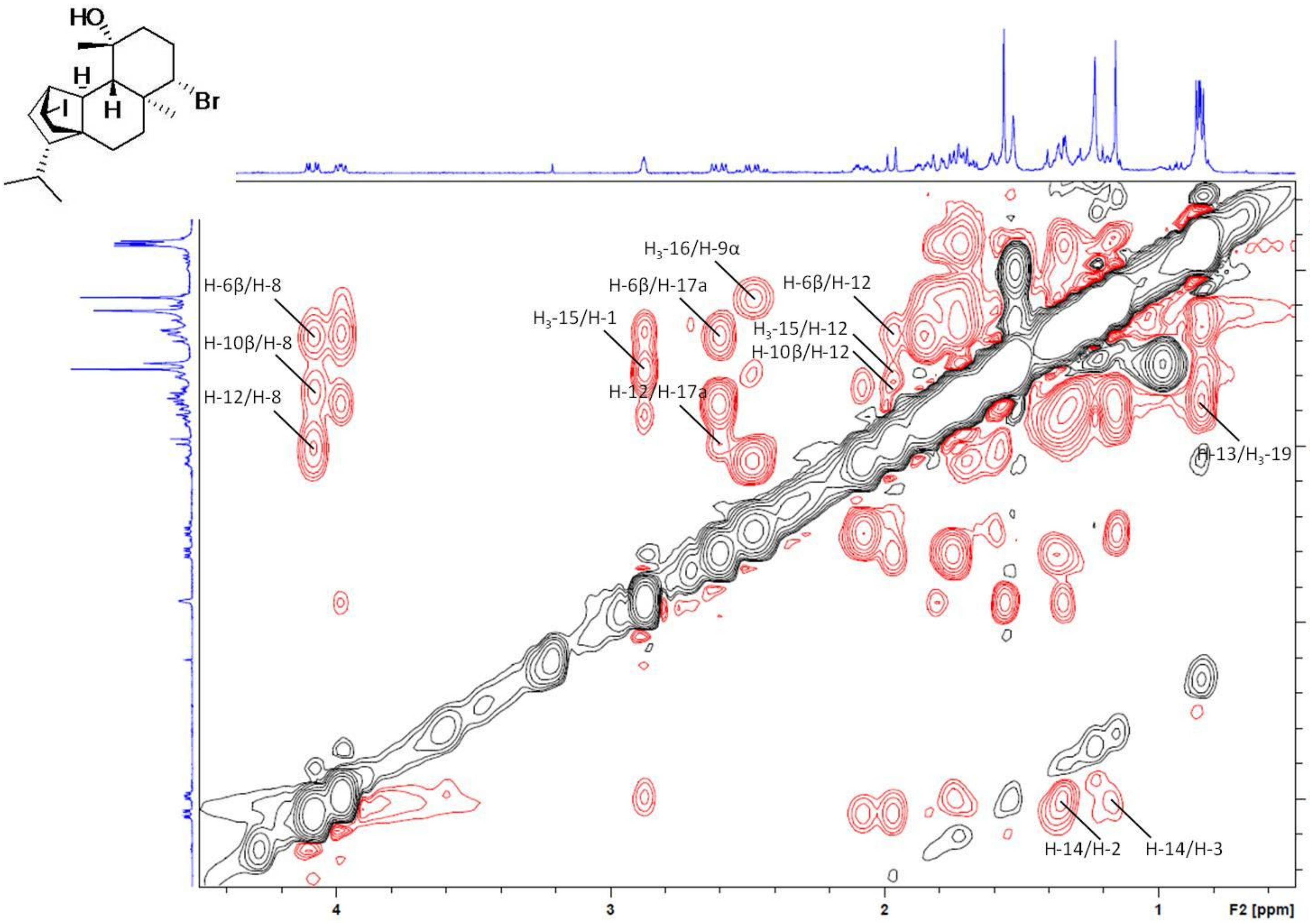

Figure S7. HRMS (ESI-) measurement of iodocoronol (**1**).

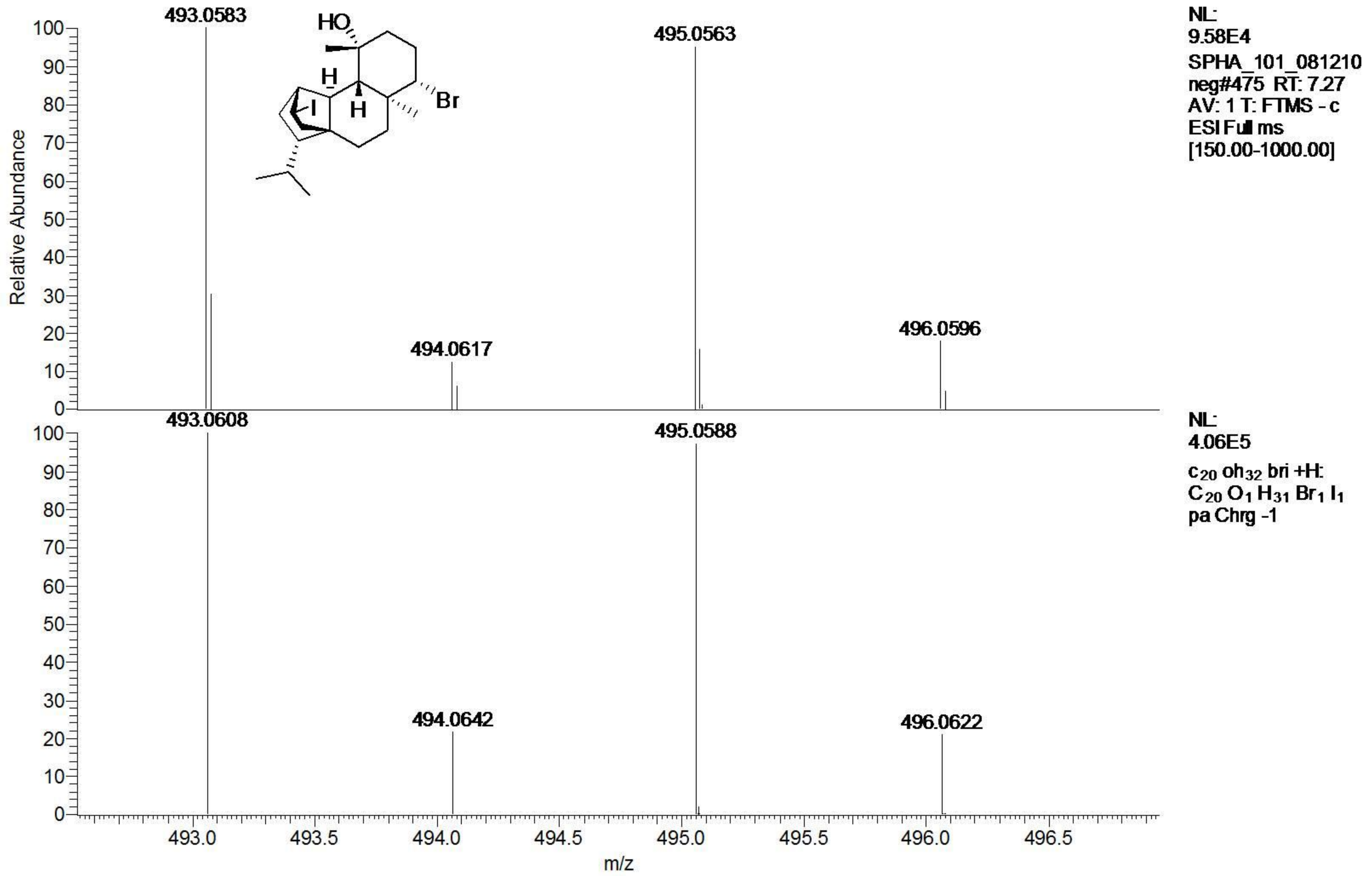

Figure S8. IR spectrum of iodocoronol (**1**).

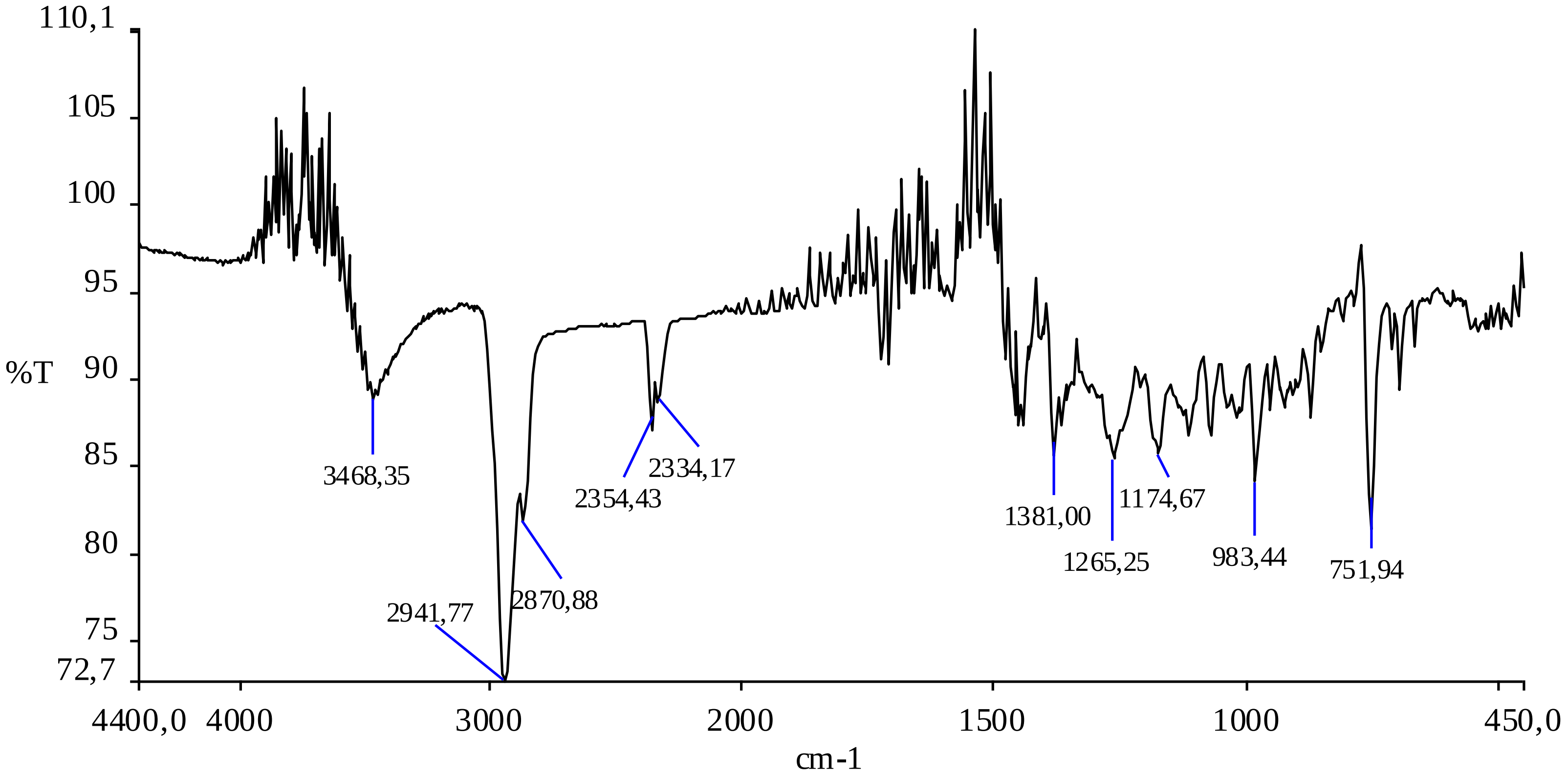

Figure S9. ^{1}H NMR spectrum (400 MHz, $CDCl_3$) of bromocoronol (**2**).

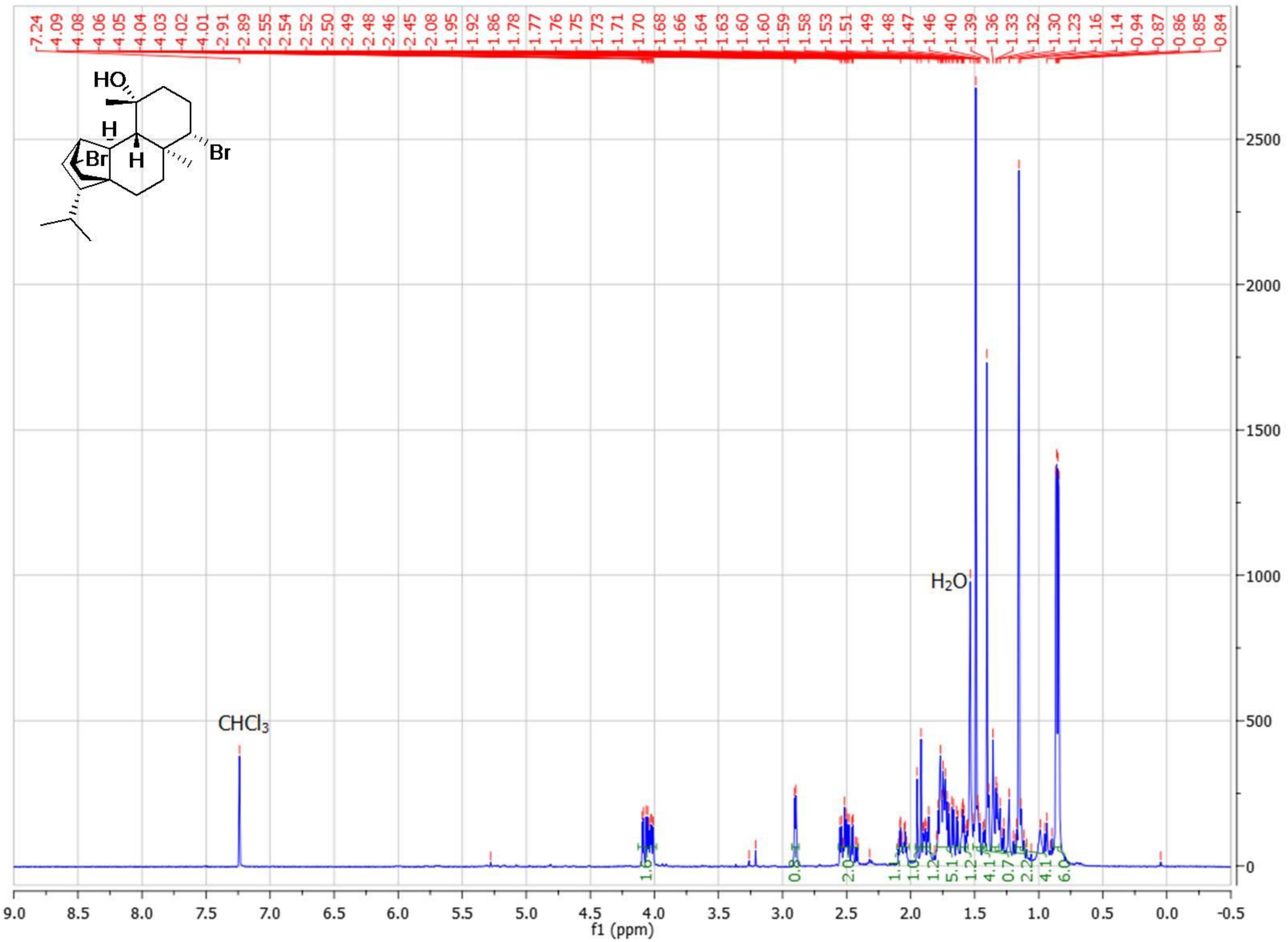

Figure S10. ^{13}C NMR spectrum (50 MHz, $CDCl_3$) of bromocoronol (**2**).

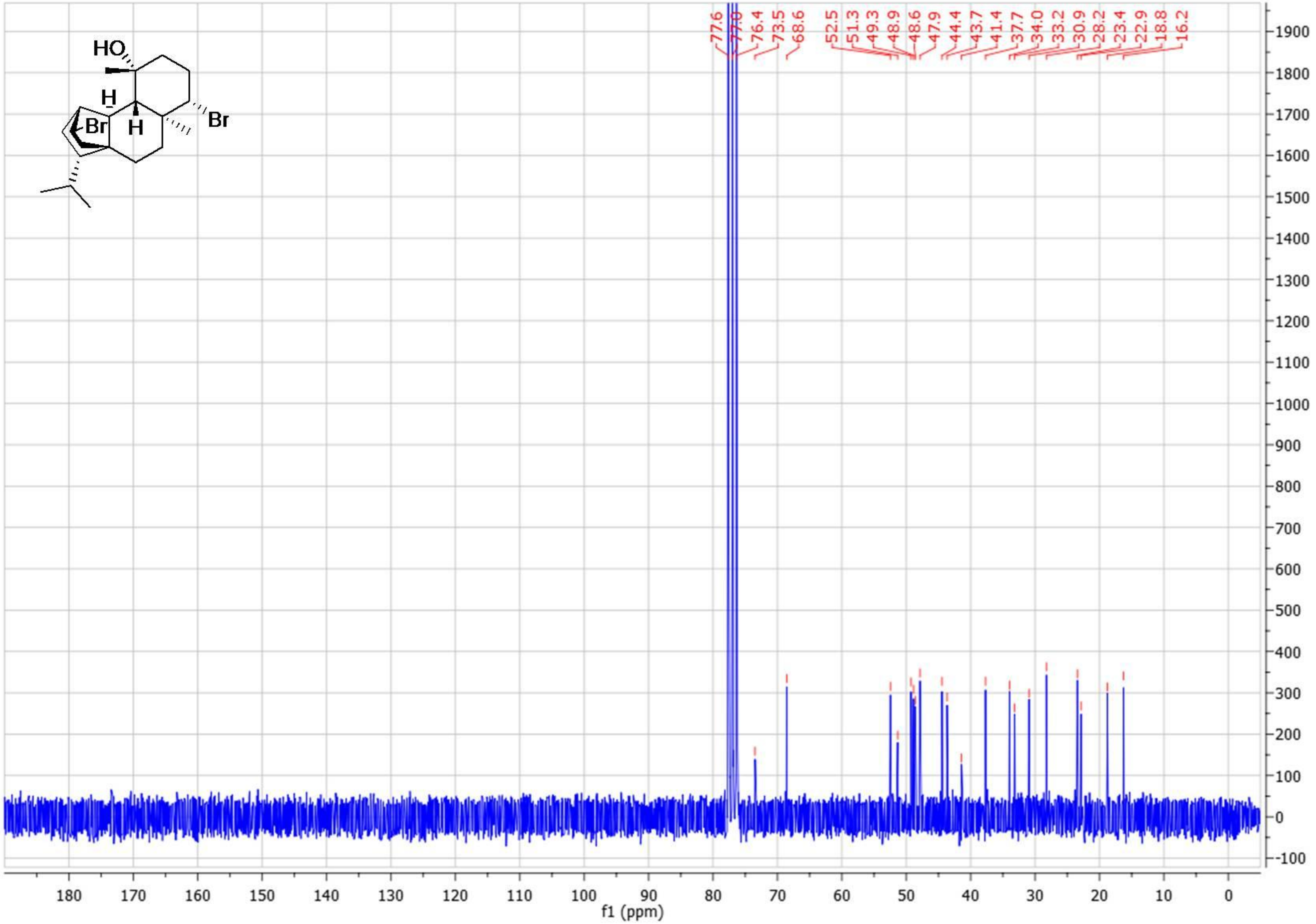

Figure S11. DEPT-135 spectrum (50 MHz, $CDCl_3$) of bromocoronol (**2**).

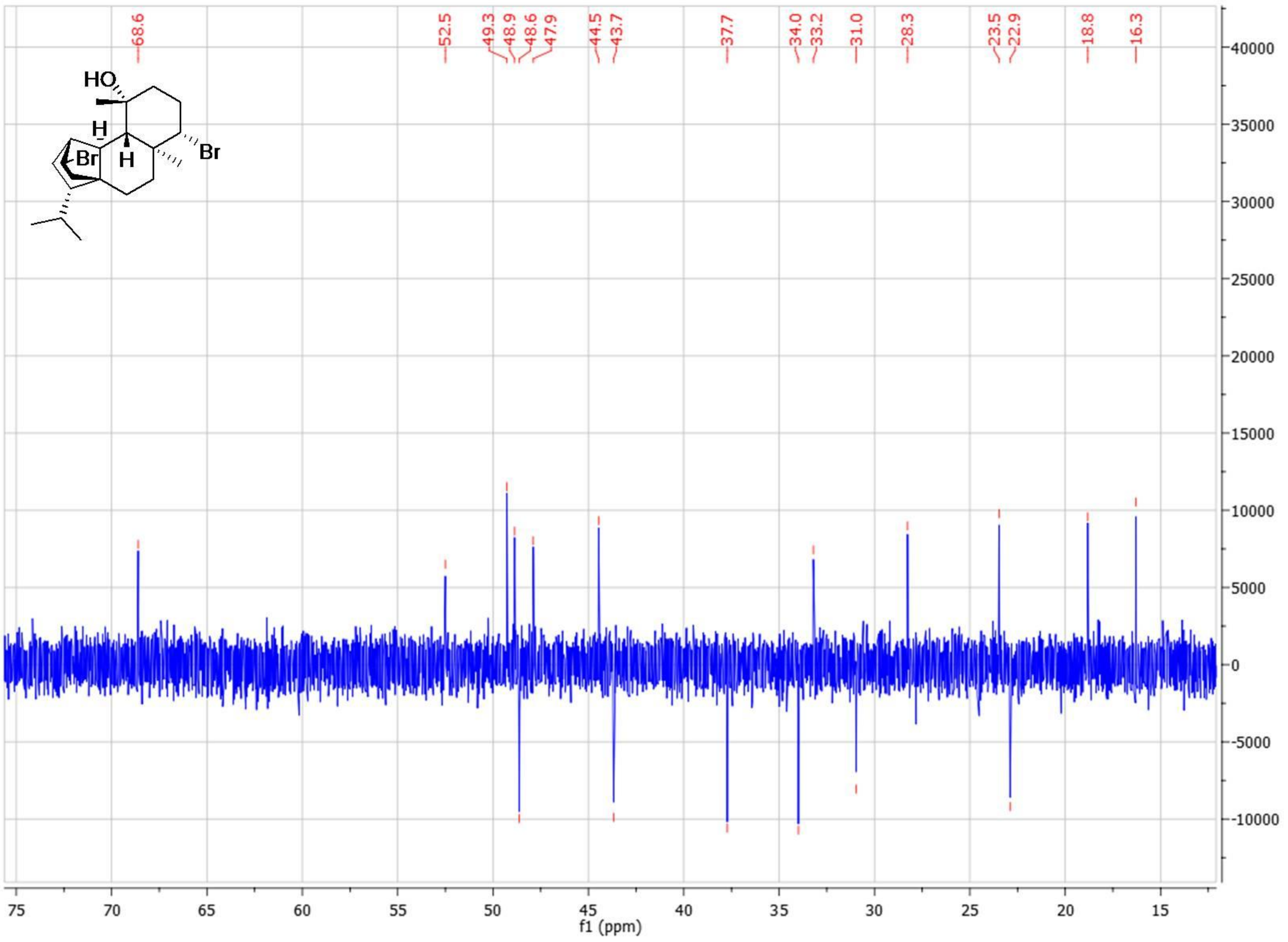

Figure S12. COSY spectrum (400 MHz, $CDCl_3$) of bromocoronol (**2**).

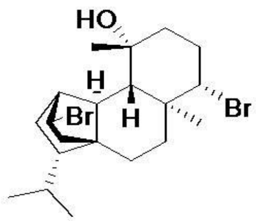

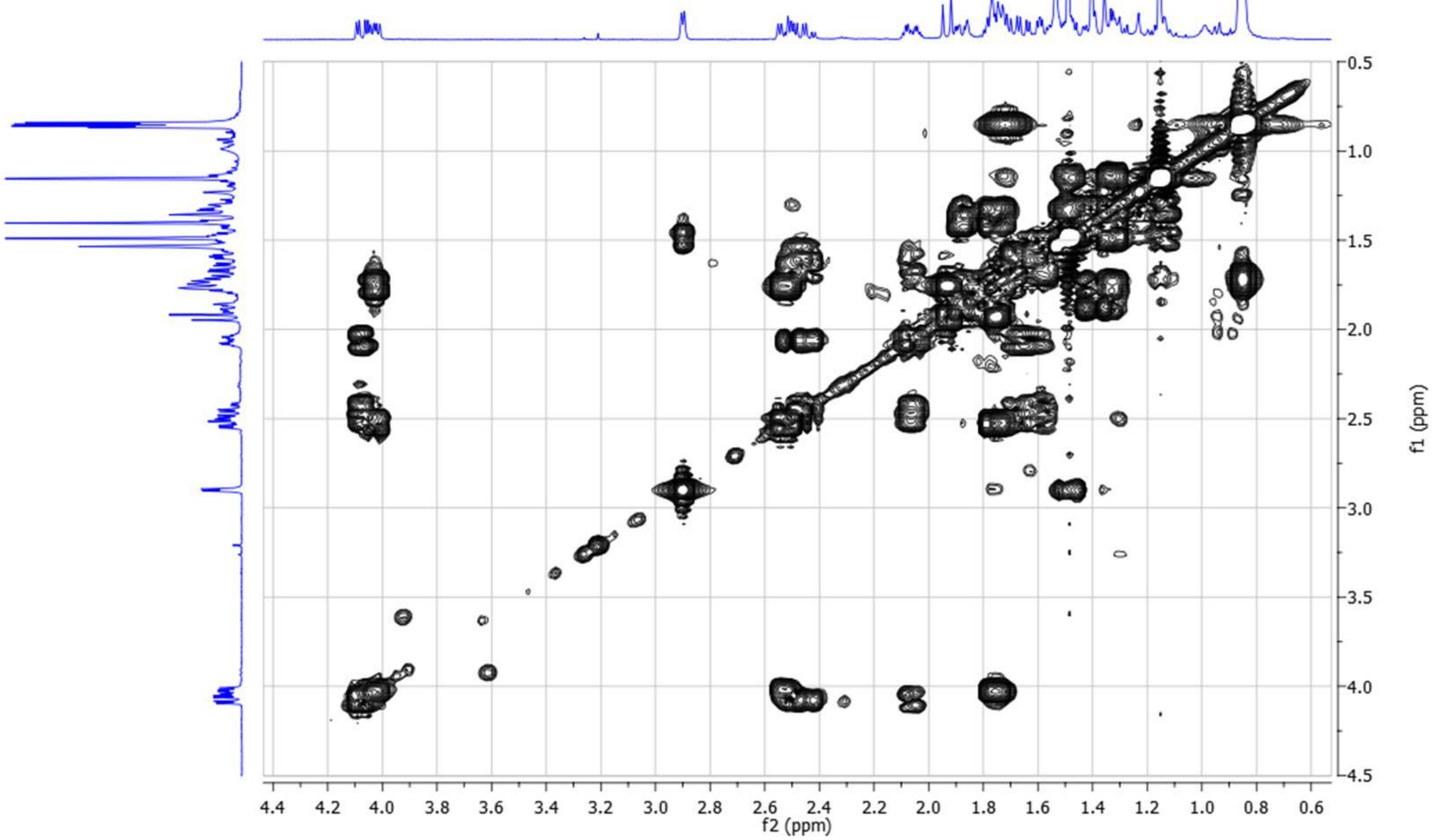

Figure S13. HSQC spectrum (400 MHz, $CDCl_3$) of bromocoronol (**2**).

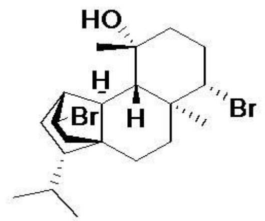

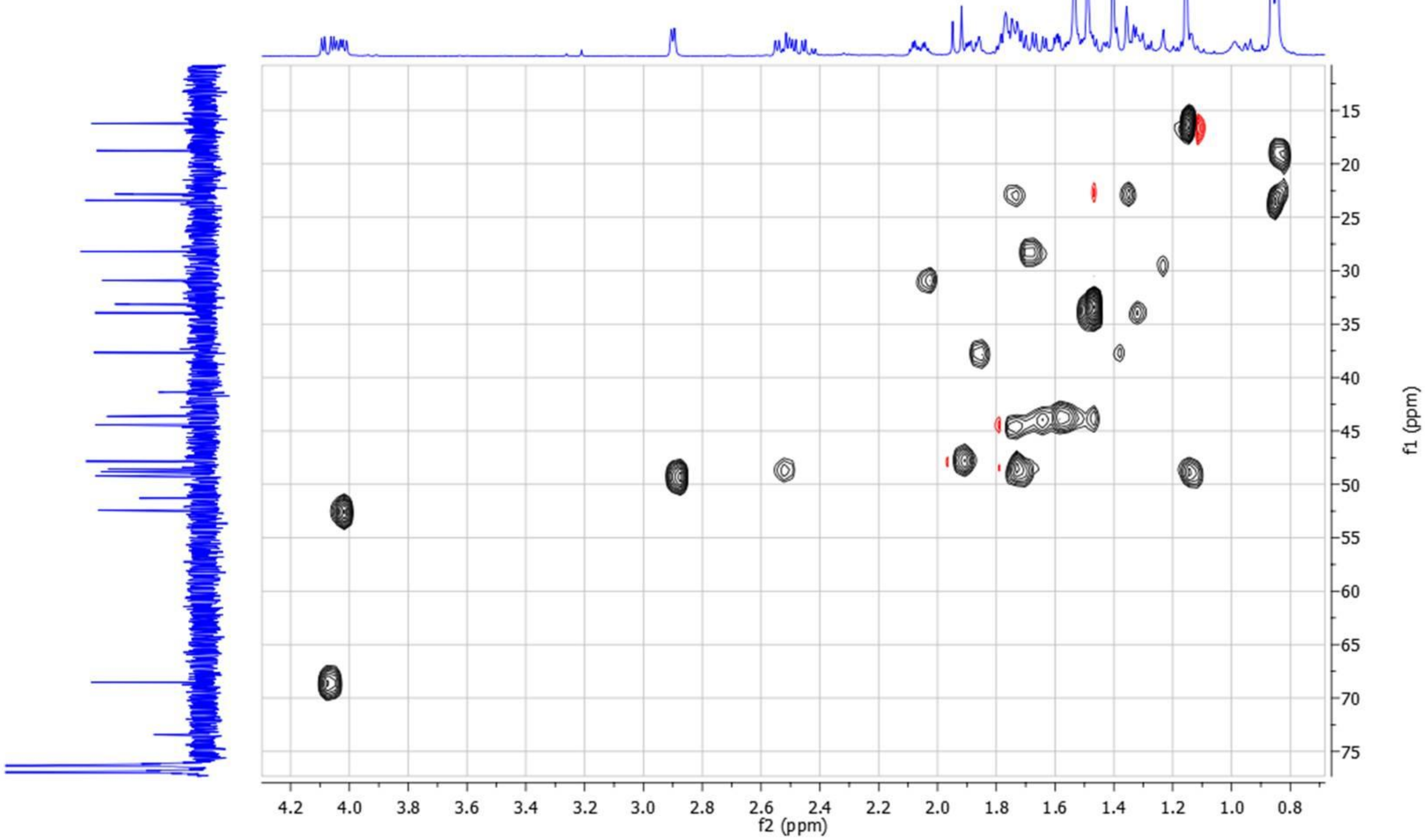

Figure S14. HSQC-TOCSY spectrum (400 MHz, $CDCl_3$) of bromocoronol (**2**).

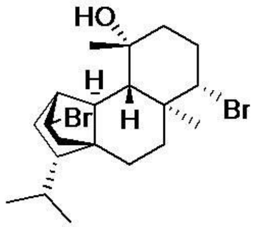

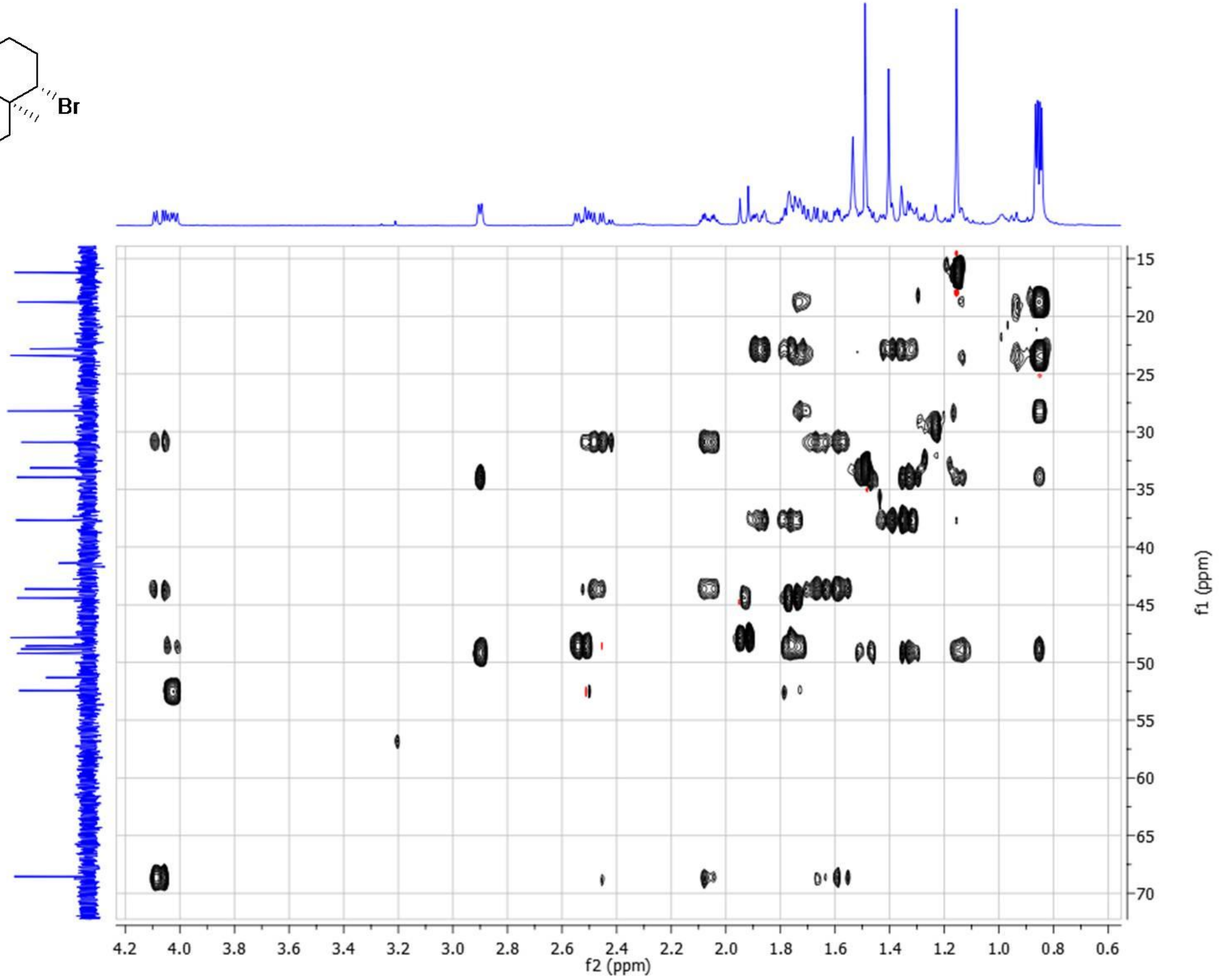

Figure S15. HMBC spectrum (400 MHz, $CDCl_3$) of bromocoronol (**2**).

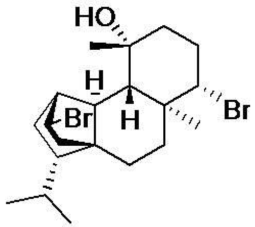

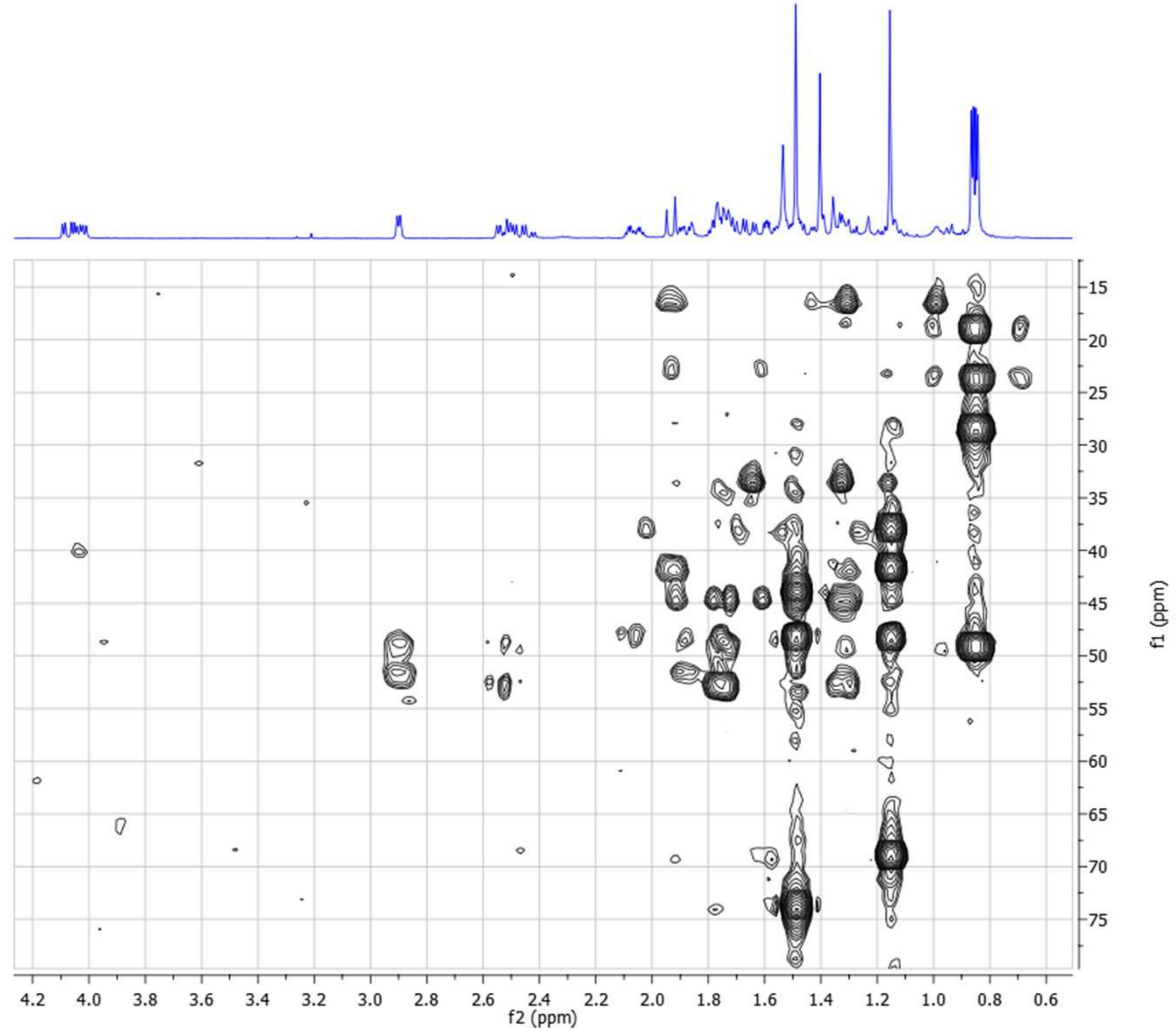

Figure S16. NOESY spectrum (400 MHz, $CDCl_3$) of bromocoronol (**2**).

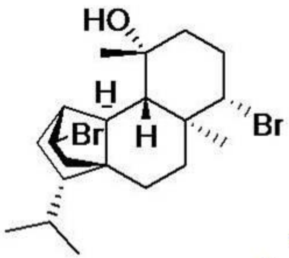

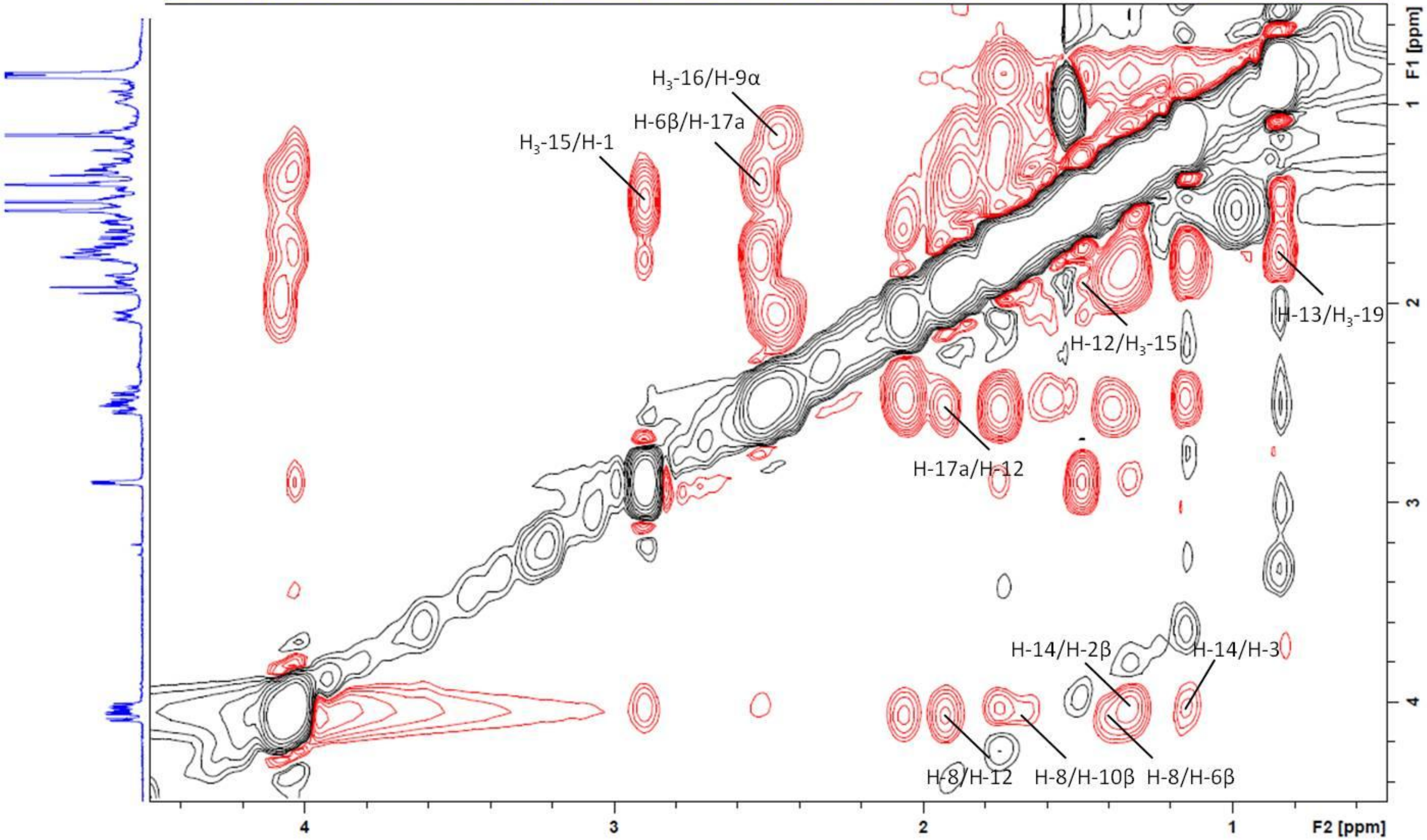

Figure S17. HRMS (ESI+) measurement of bromocoronol (**2**).

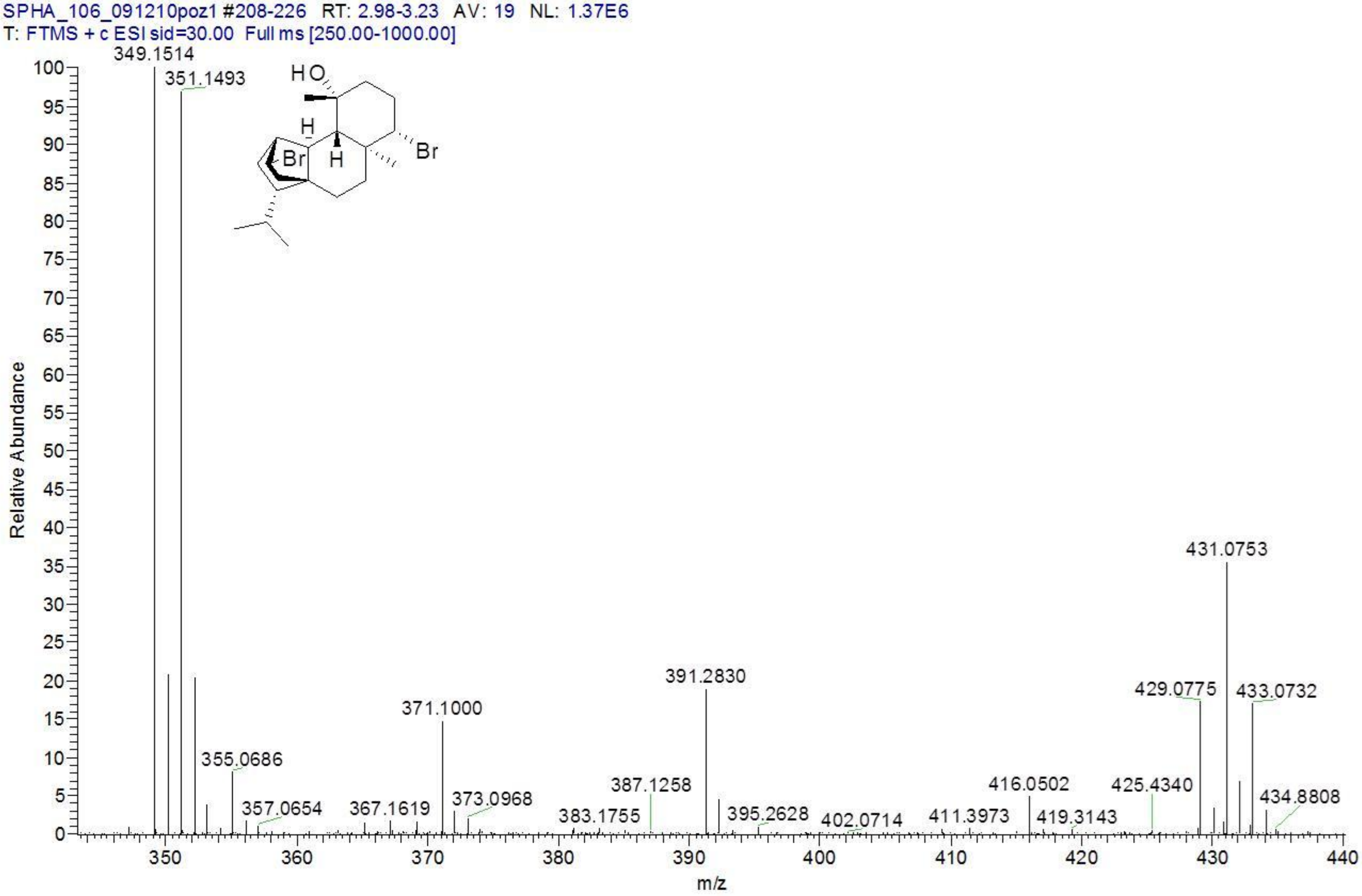

Figure S18. IR spectrum of bromocoronol (**2**).

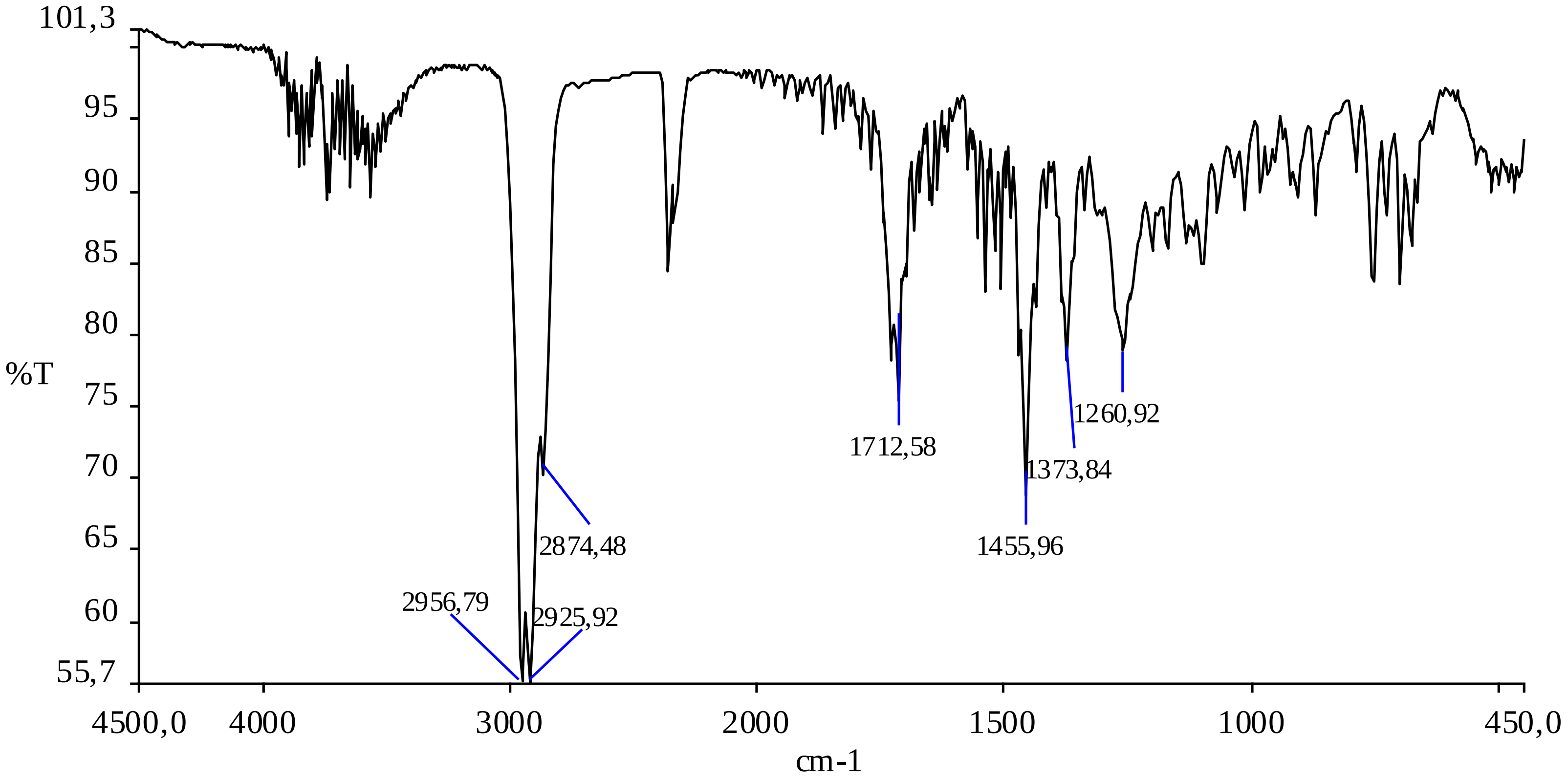

Figure S19. ^{1}H NMR spectrum (600 MHz, $CDCl_3$) of bromotetrasphaereniol (**3**).

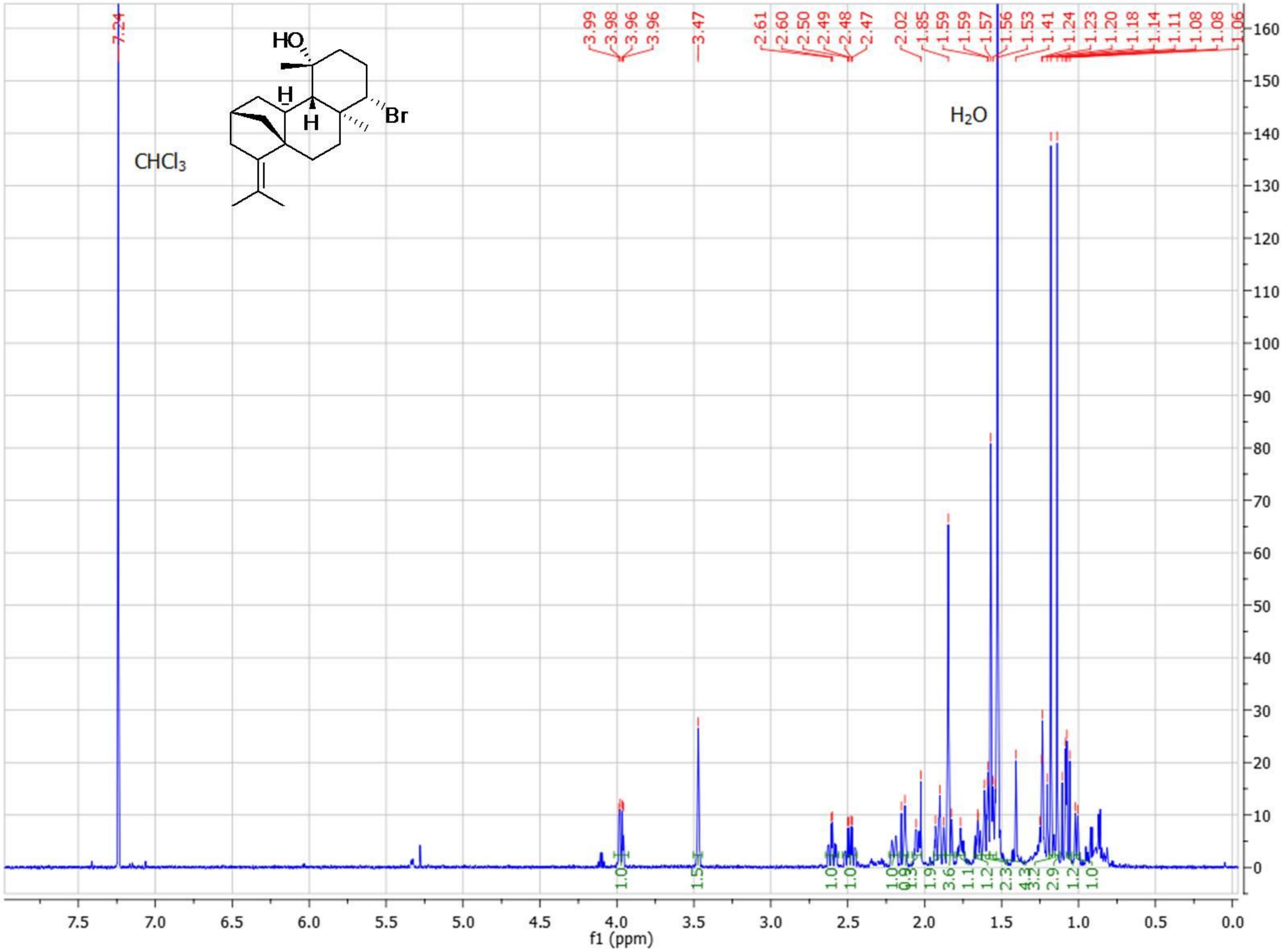

Figure S20. ^{13}C NMR spectrum (75 MHz, $CDCl_3$) of bromotetrasphaereniol (**3**).

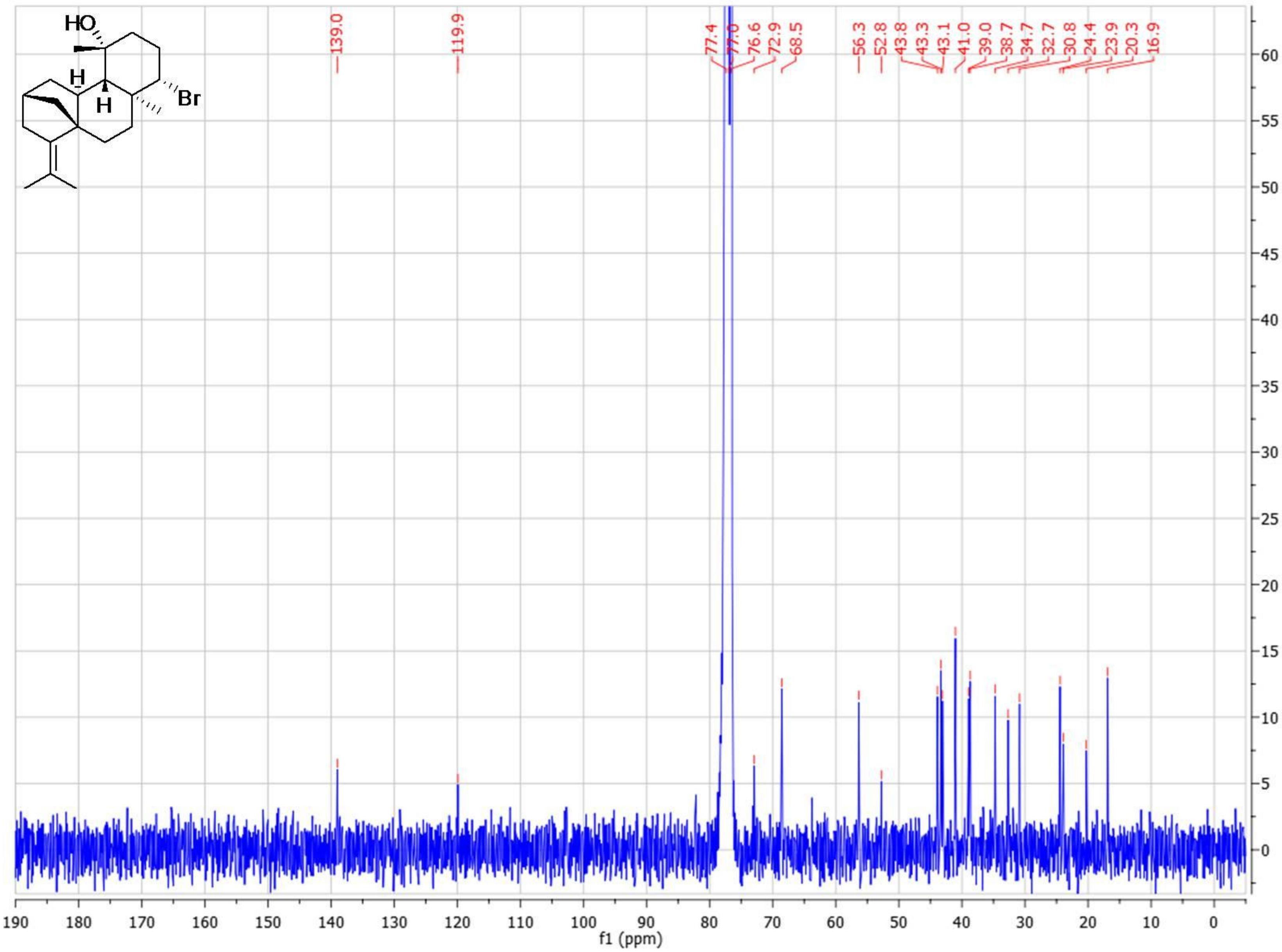

Figure S21. COSY spectrum (600 MHz, $CDCl_3$) of bromotetrasphaereniol (**3**).

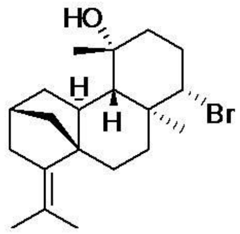

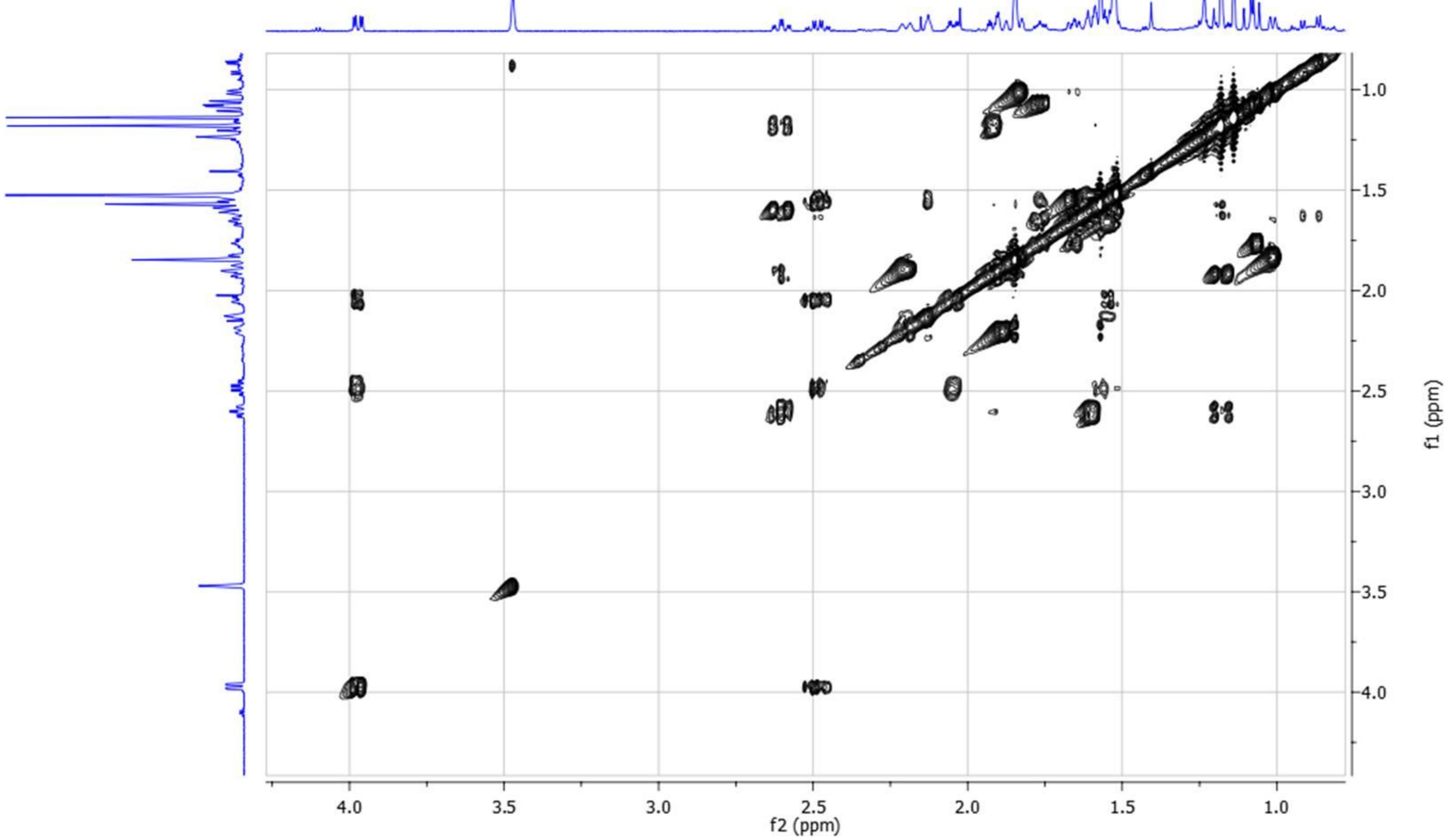

Figure S22. HSQC-DEPT spectrum (400 MHz, $CDCl_3$) of bromotetrasphaereniol (**3**).

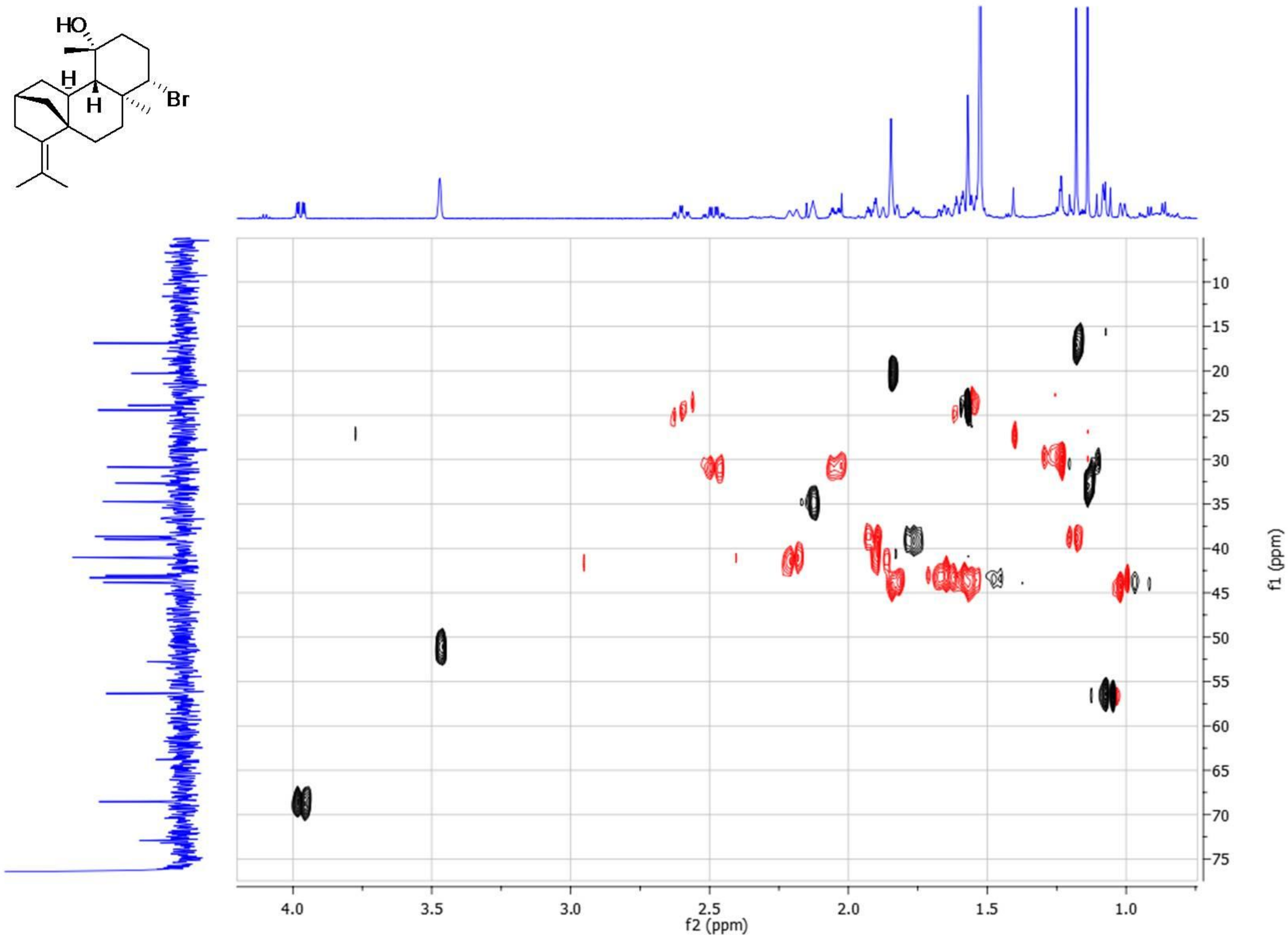

Figure S23. HMBC spectrum (600 MHz, $CDCl_3$) of bromotetrasphaereniol (**3**).

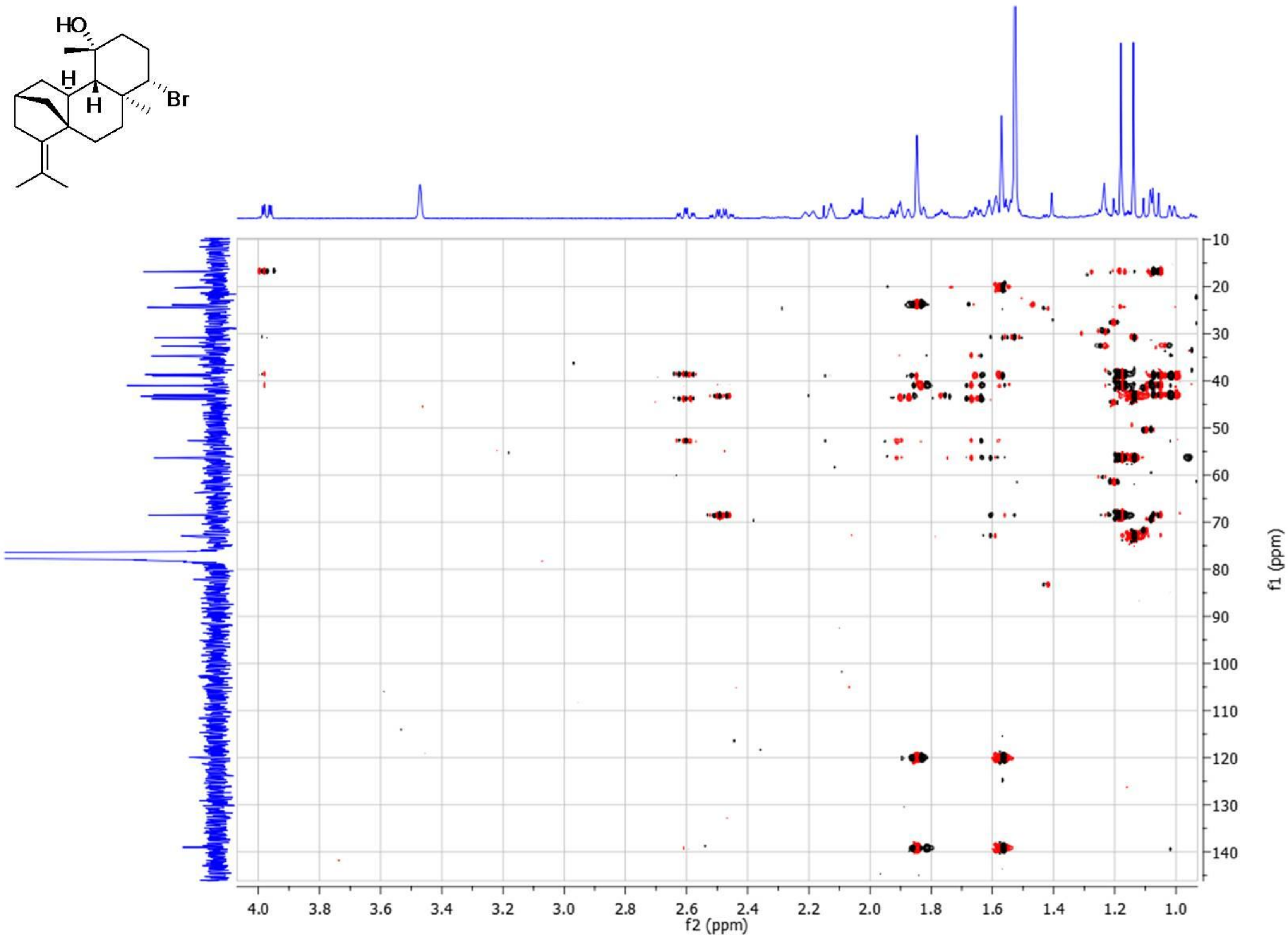

Figure S24. NOESY spectrum (600 MHz, $CDCl_3$) of bromotetrasphaerenol (**3**).

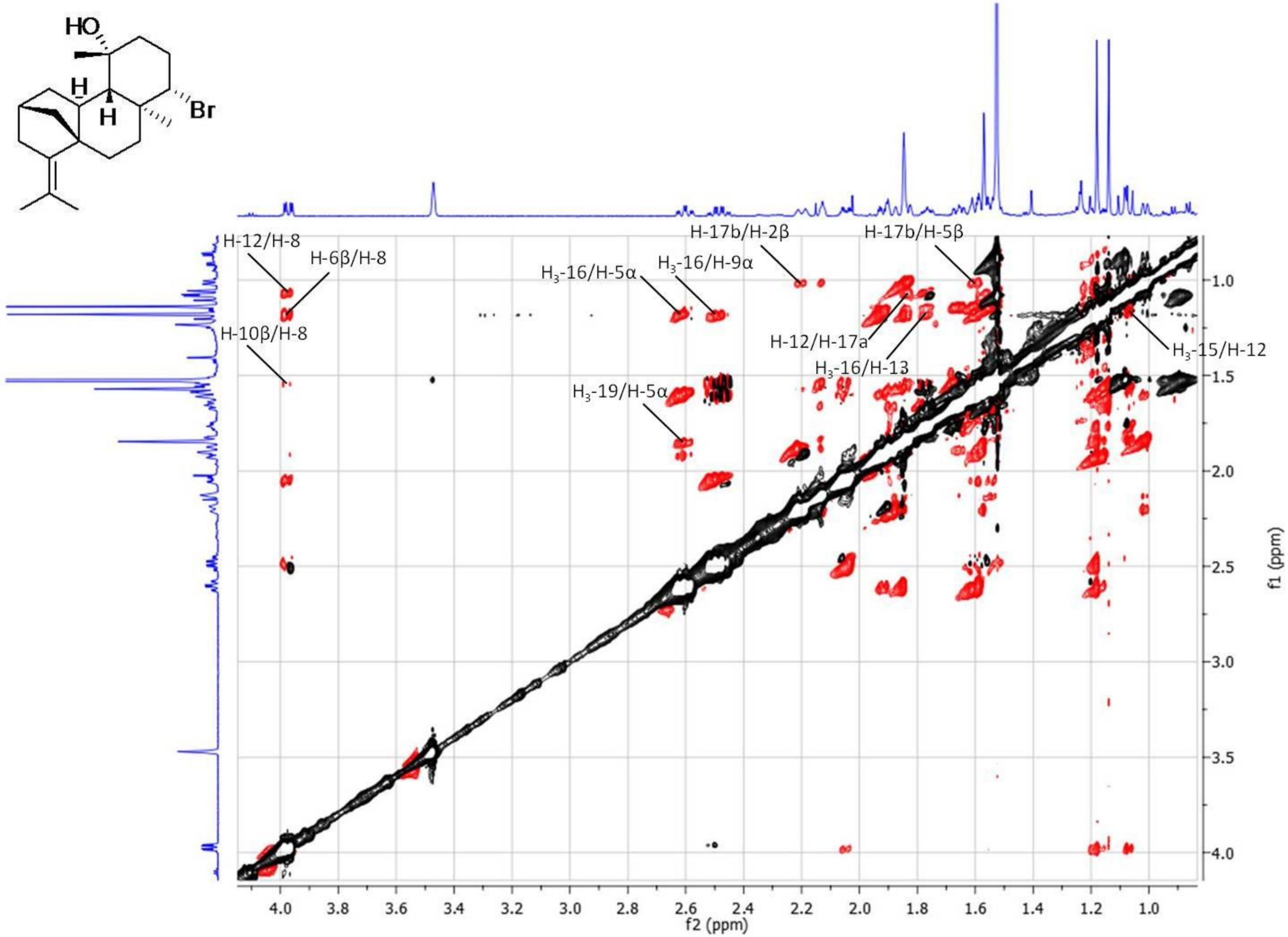

Figure S25. HRMS (ESI+) measurement of bromotetrasphaereniol (**3**).

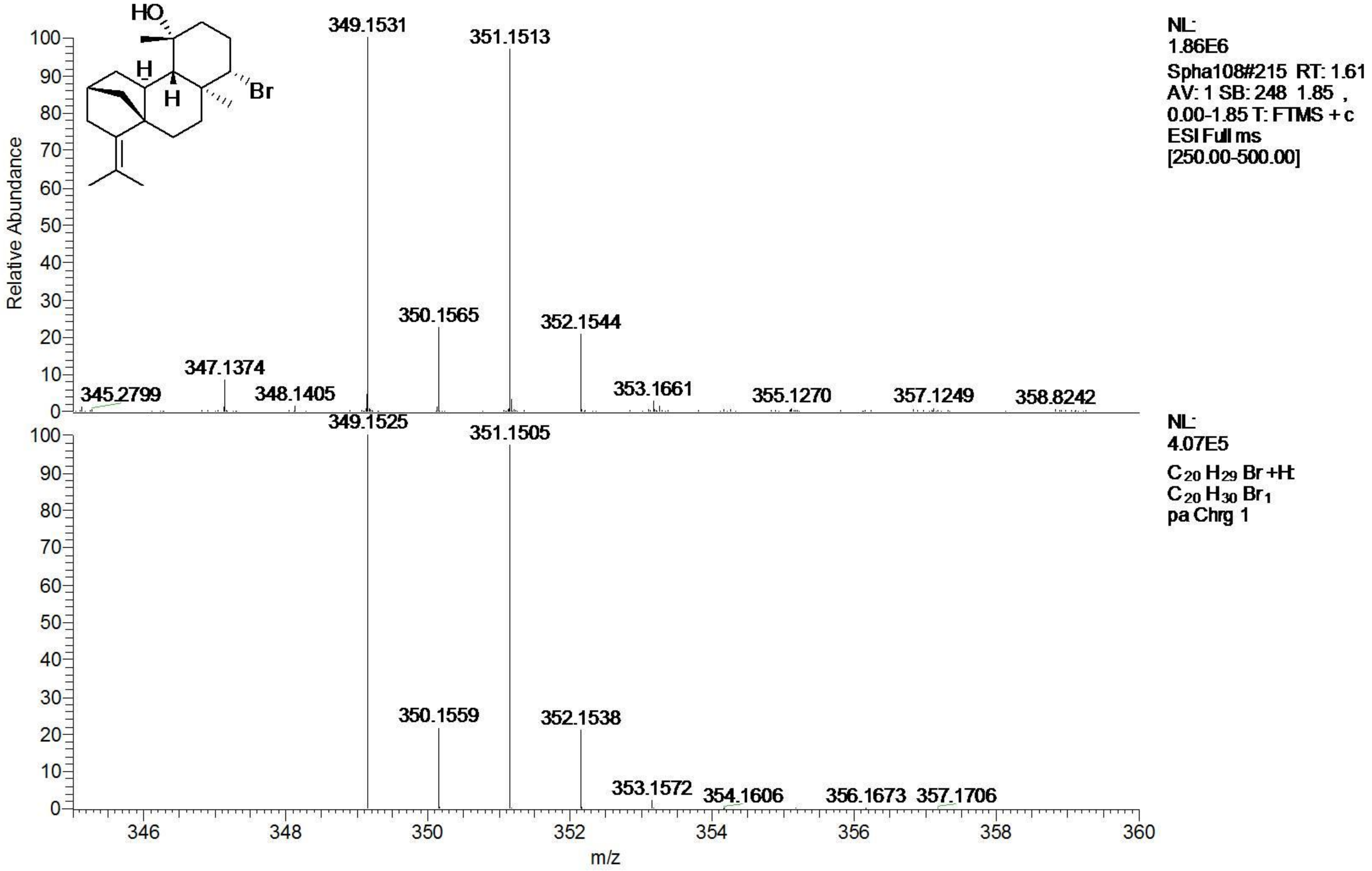

Figure S26. IR spectrum of bromotetrasphaereniol (**3**).

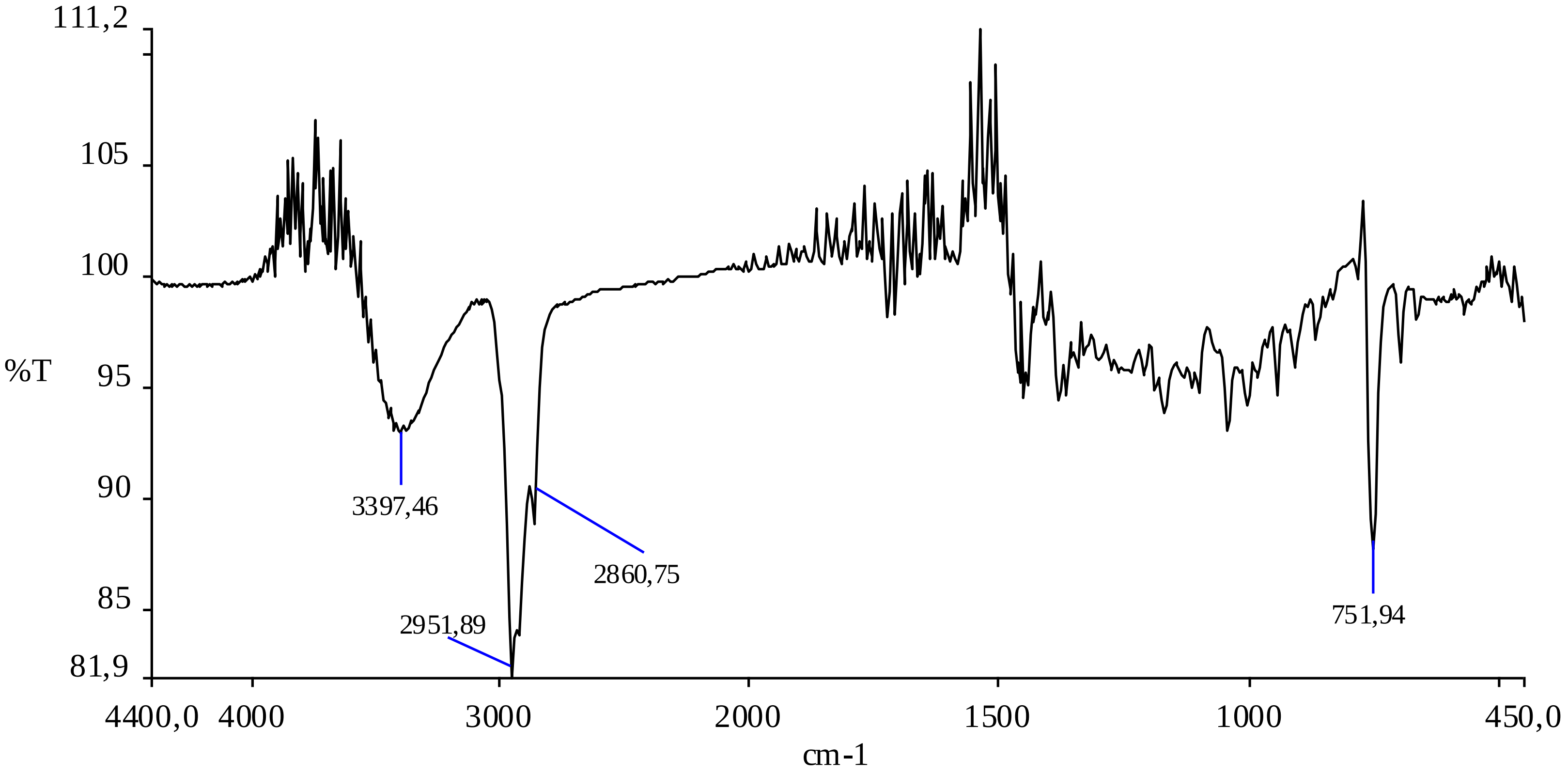

Figure S27. ^{1}H NMR spectrum (400 MHz, $CDCl_3$) of 1-methoxy-ioniol I (**4**).

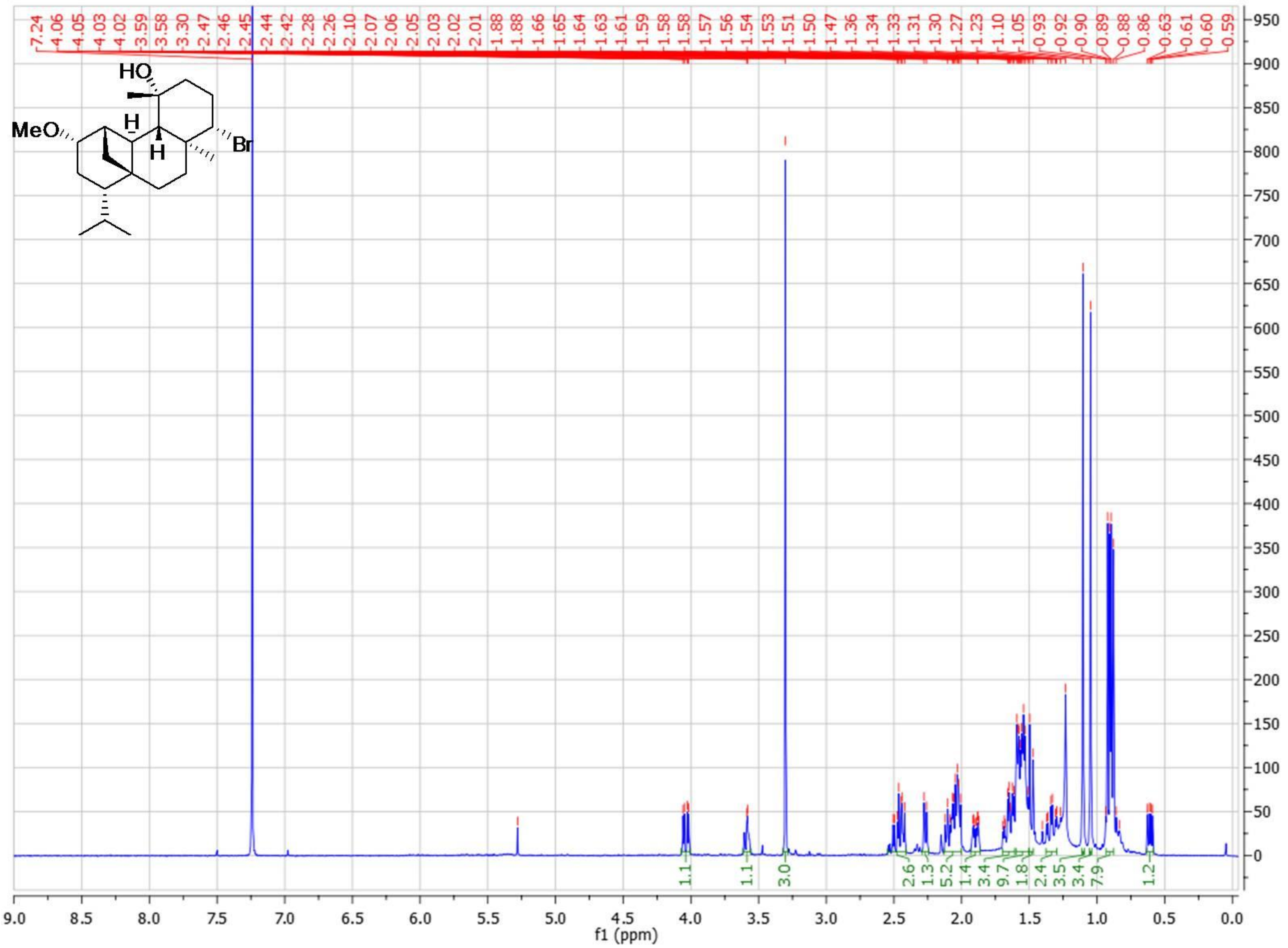

Figure S28. ^{13}C NMR spectrum (50 MHz, $CDCl_3$) of 1-methoxy-ioniol I (**4**).

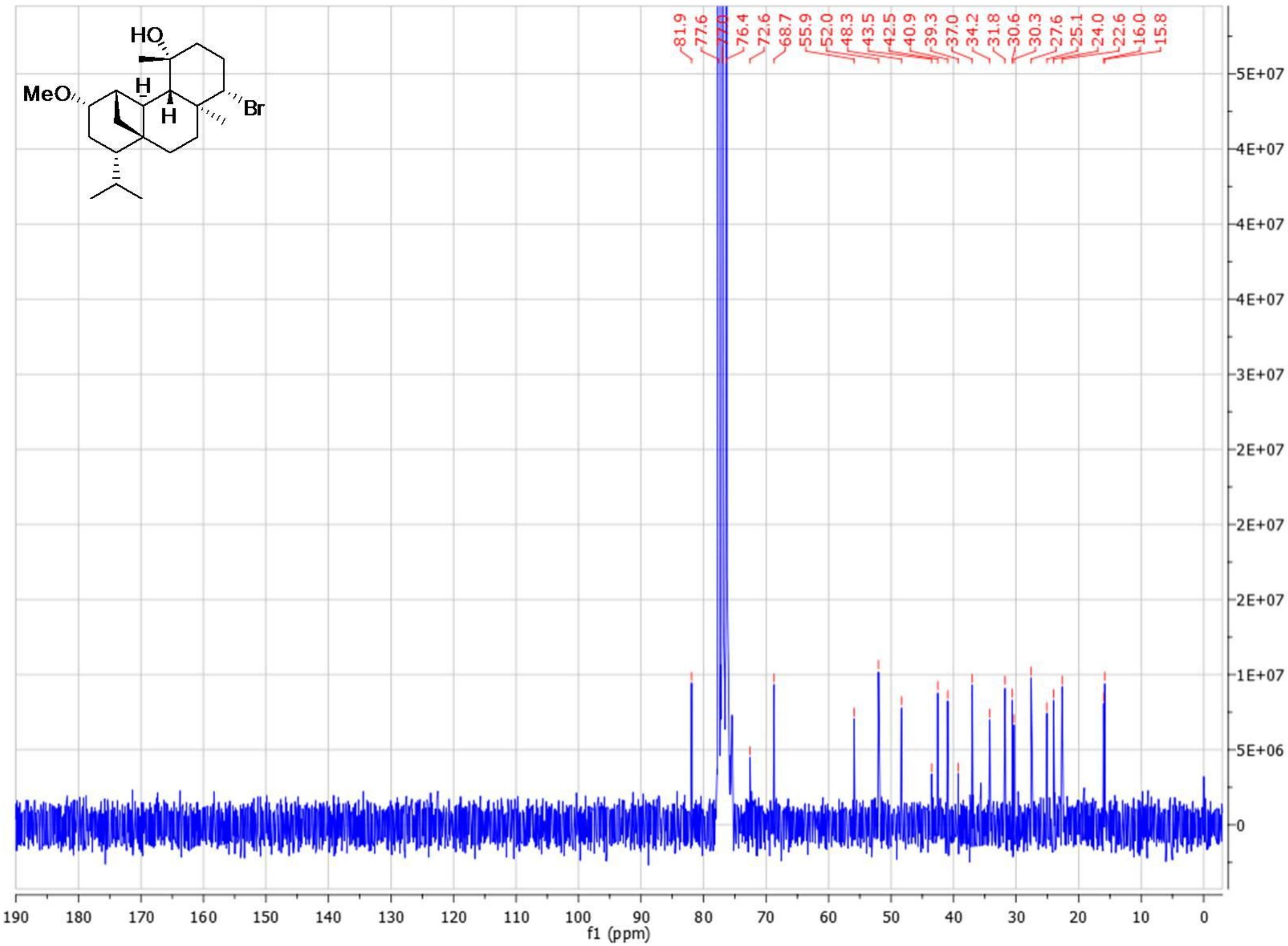

Figure S29. COSY spectrum (400 MHz, $CDCl_3$) of 1-methoxy-ioniol I (**4**).

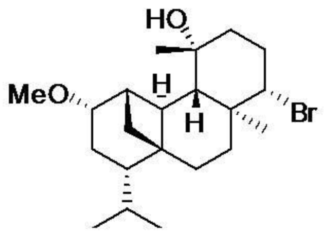

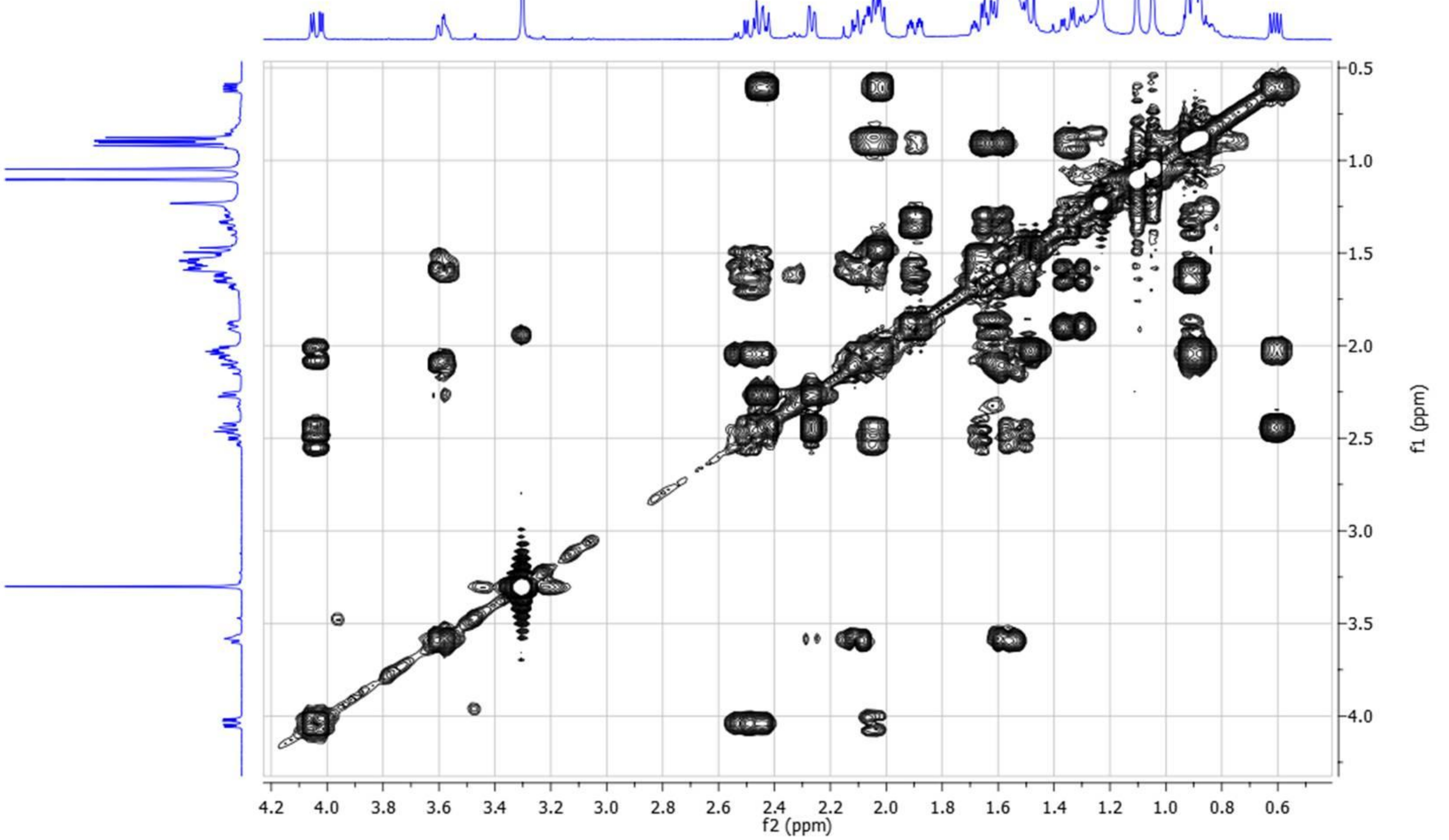

Figure S30. HSQC spectrum (400 MHz, $CDCl_3$) of 1-methoxy-ioniol I (**4**).

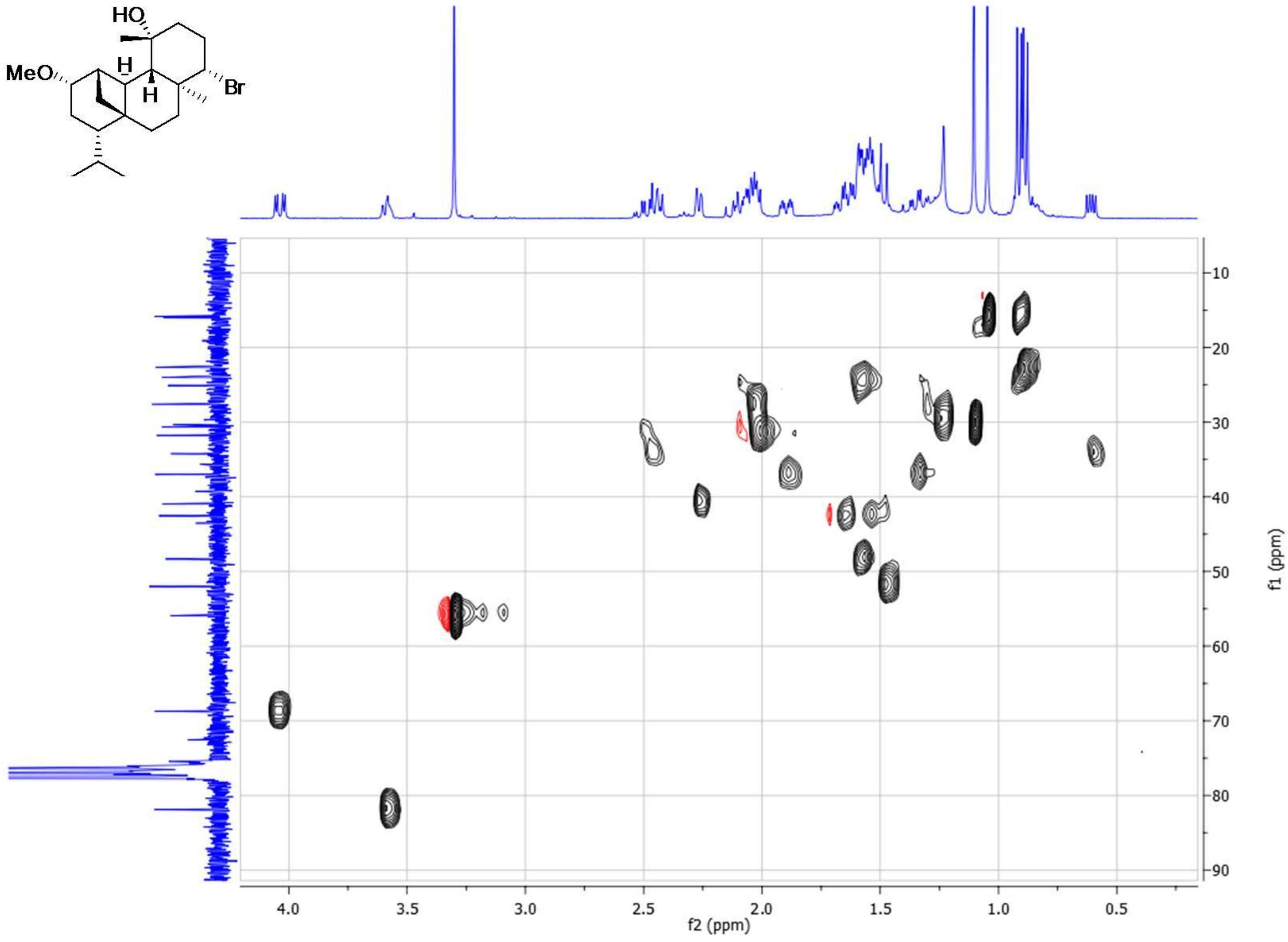

Figure S31. HMBC spectrum (400 MHz, $CDCl_3$) of 1-methoxy-ioniol I (**4**).

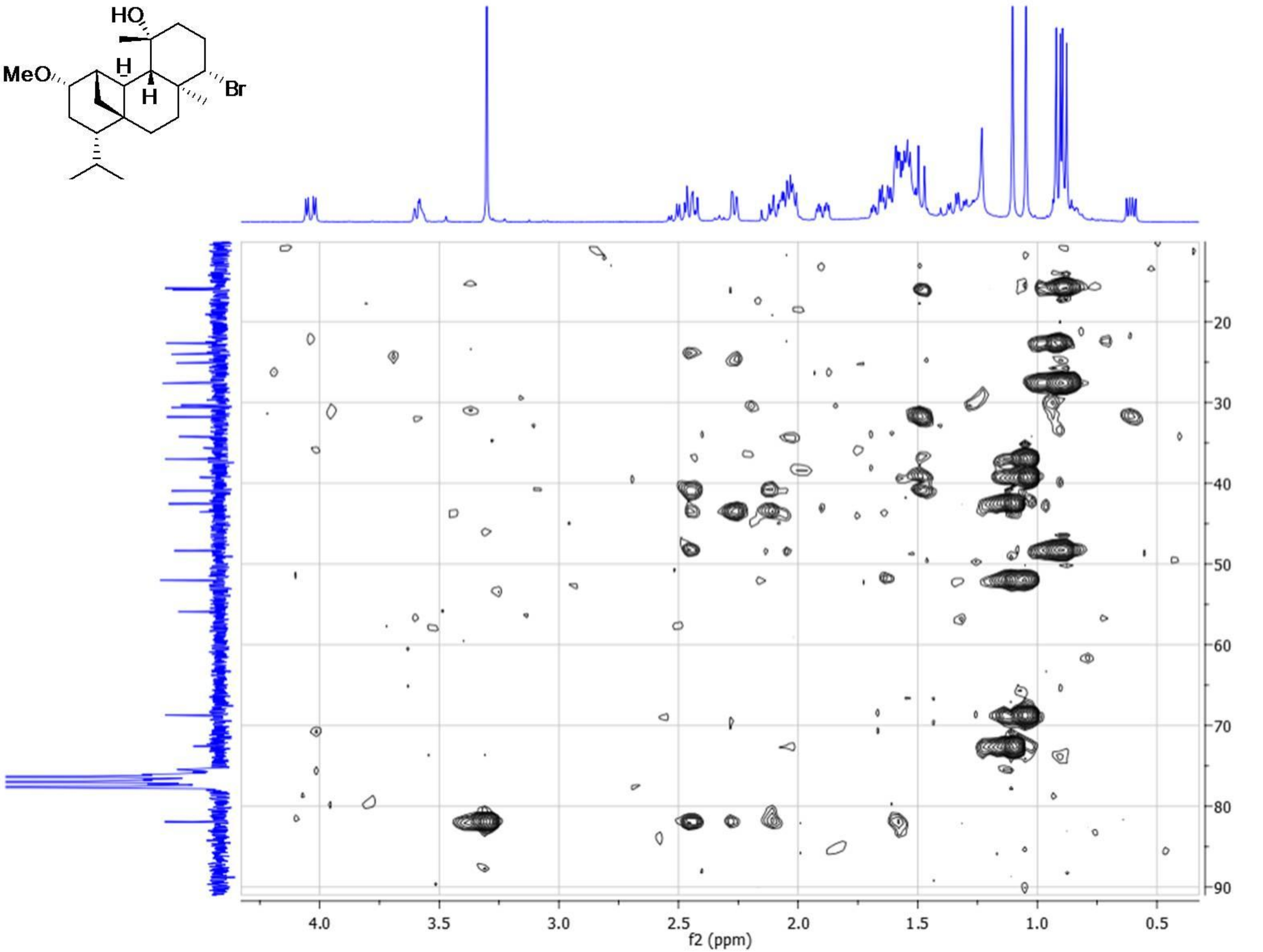

Figure S32. NOESY spectrum (400 MHz, $CDCl_3$) of 1-methoxy-ioniol I (**4**).

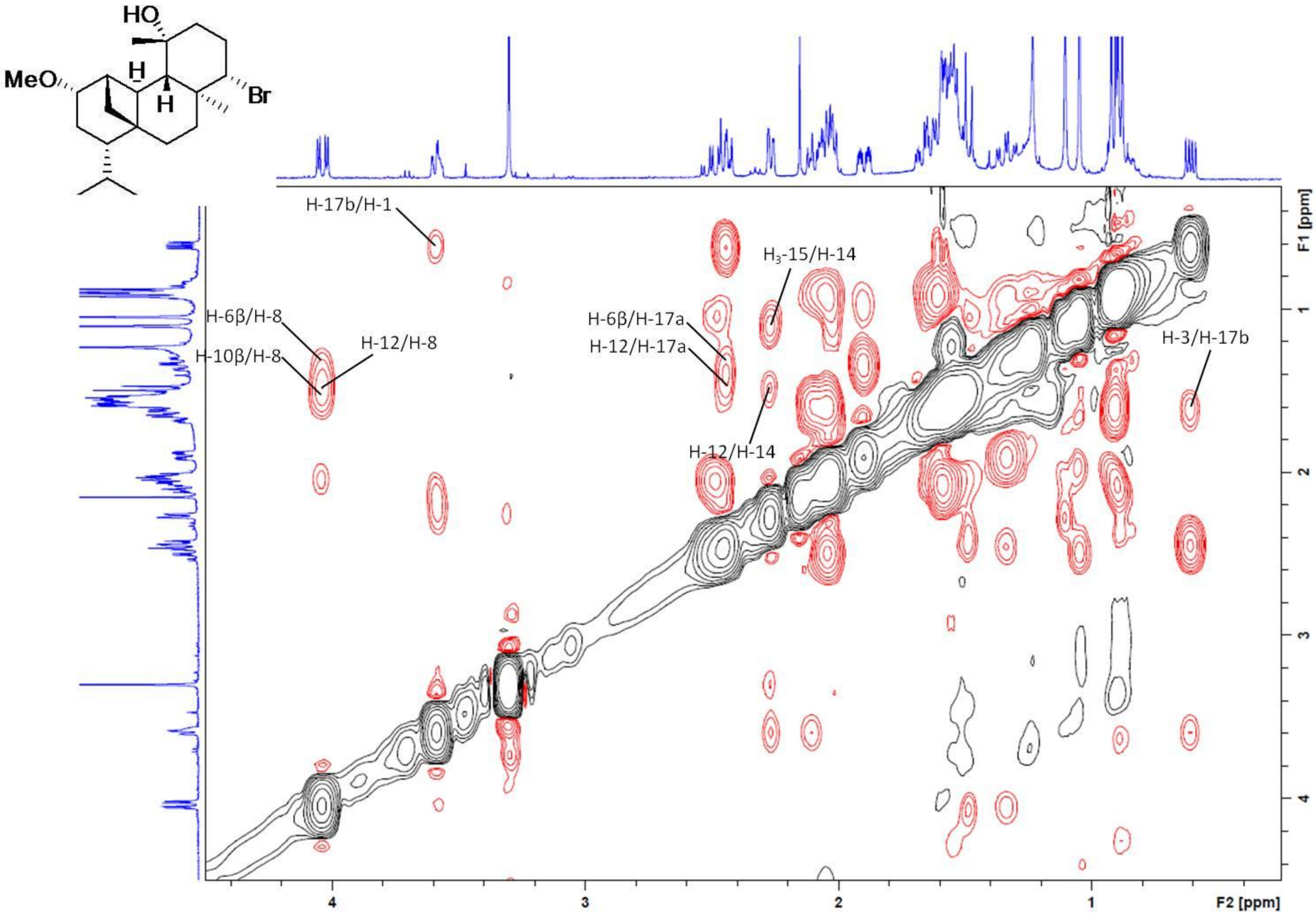

Figure S33. HRMS (ESI+) measurement of 1-methoxy-ioniol I (**4**).

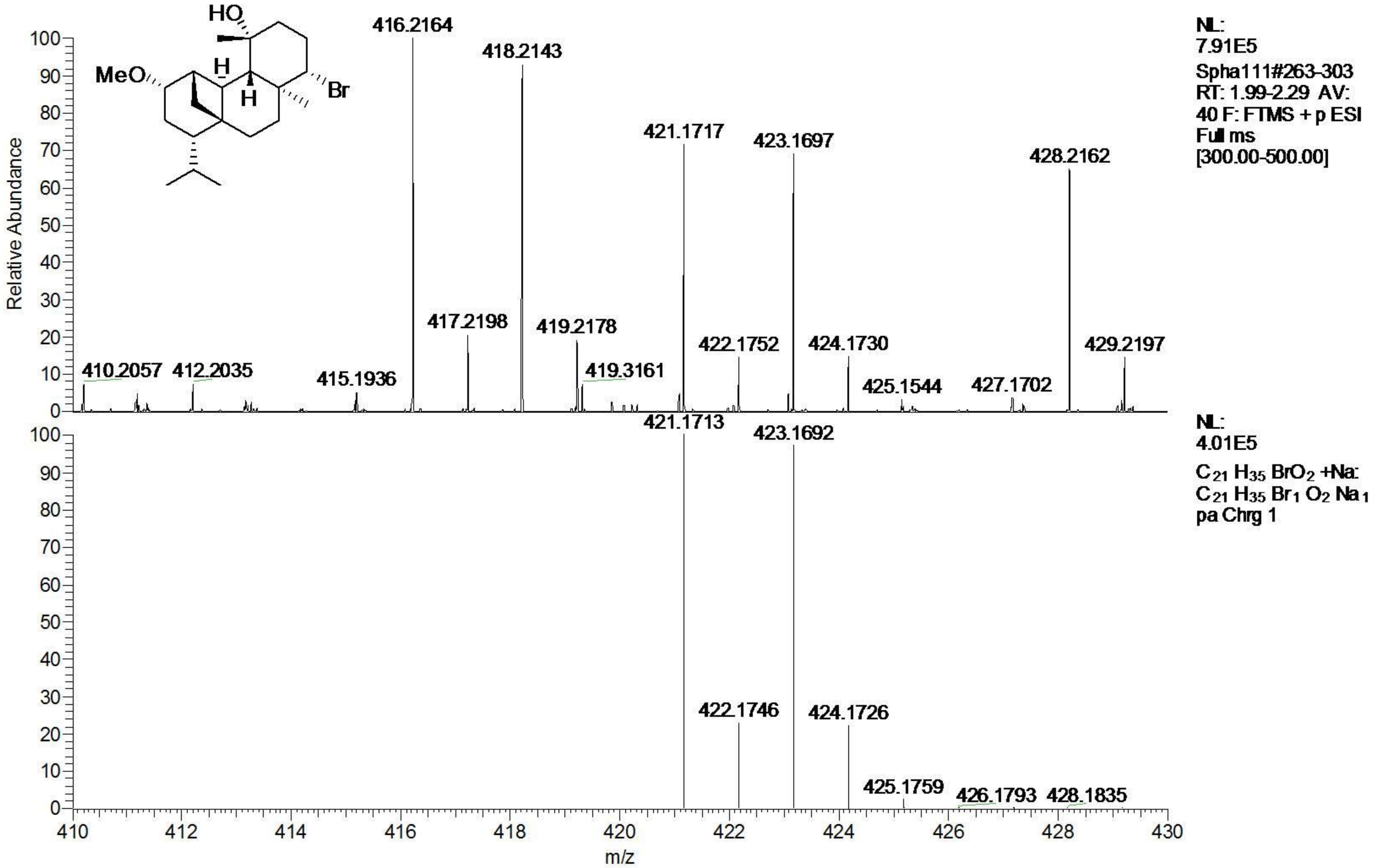

Figure S34. IR spectrum of 1-methoxy-ioniol I (**4**).

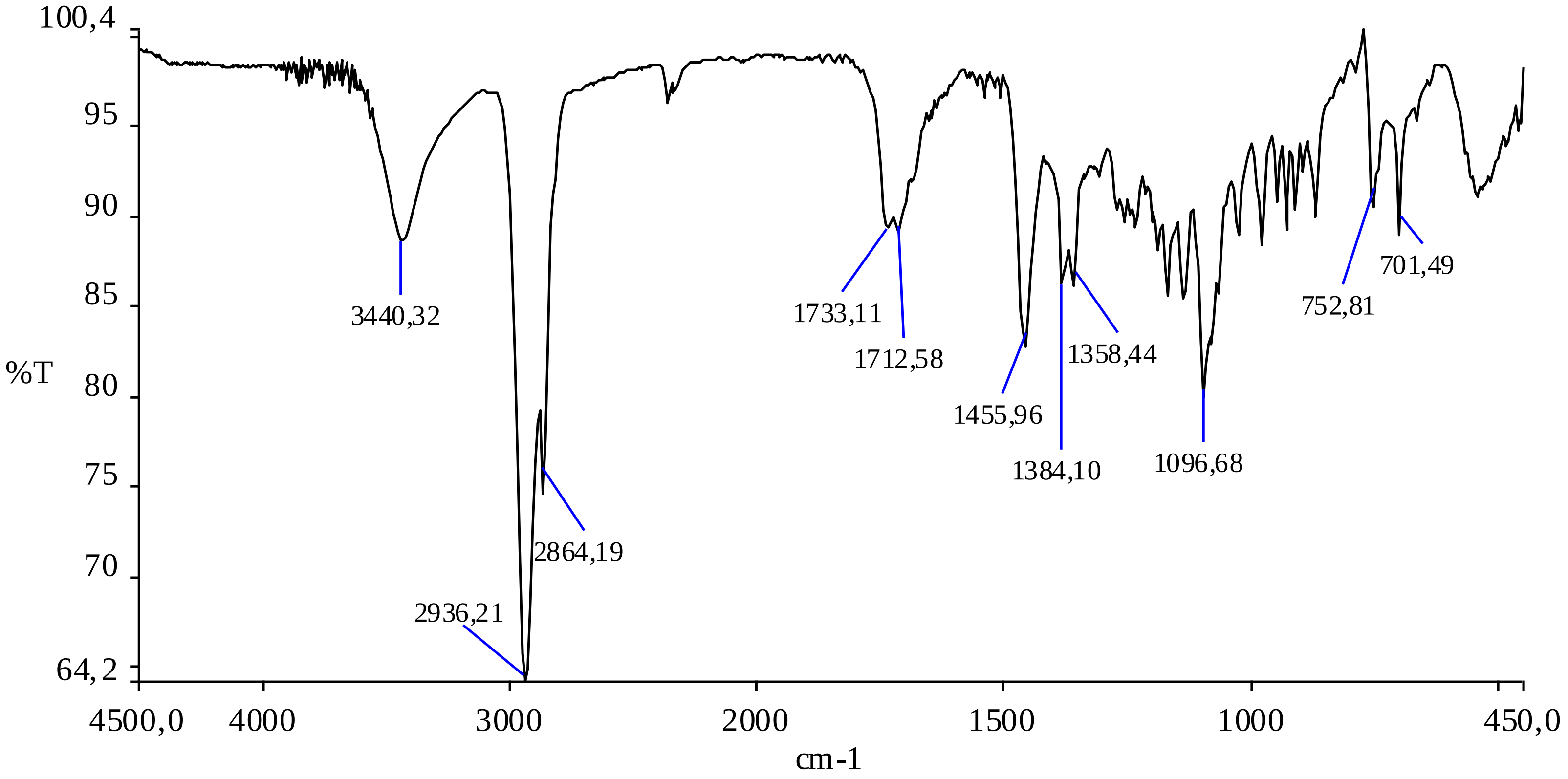

Figure S35. ^{1}H NMR spectrum (400 MHz, $CDCl_3$) of corotrienone (**5**).

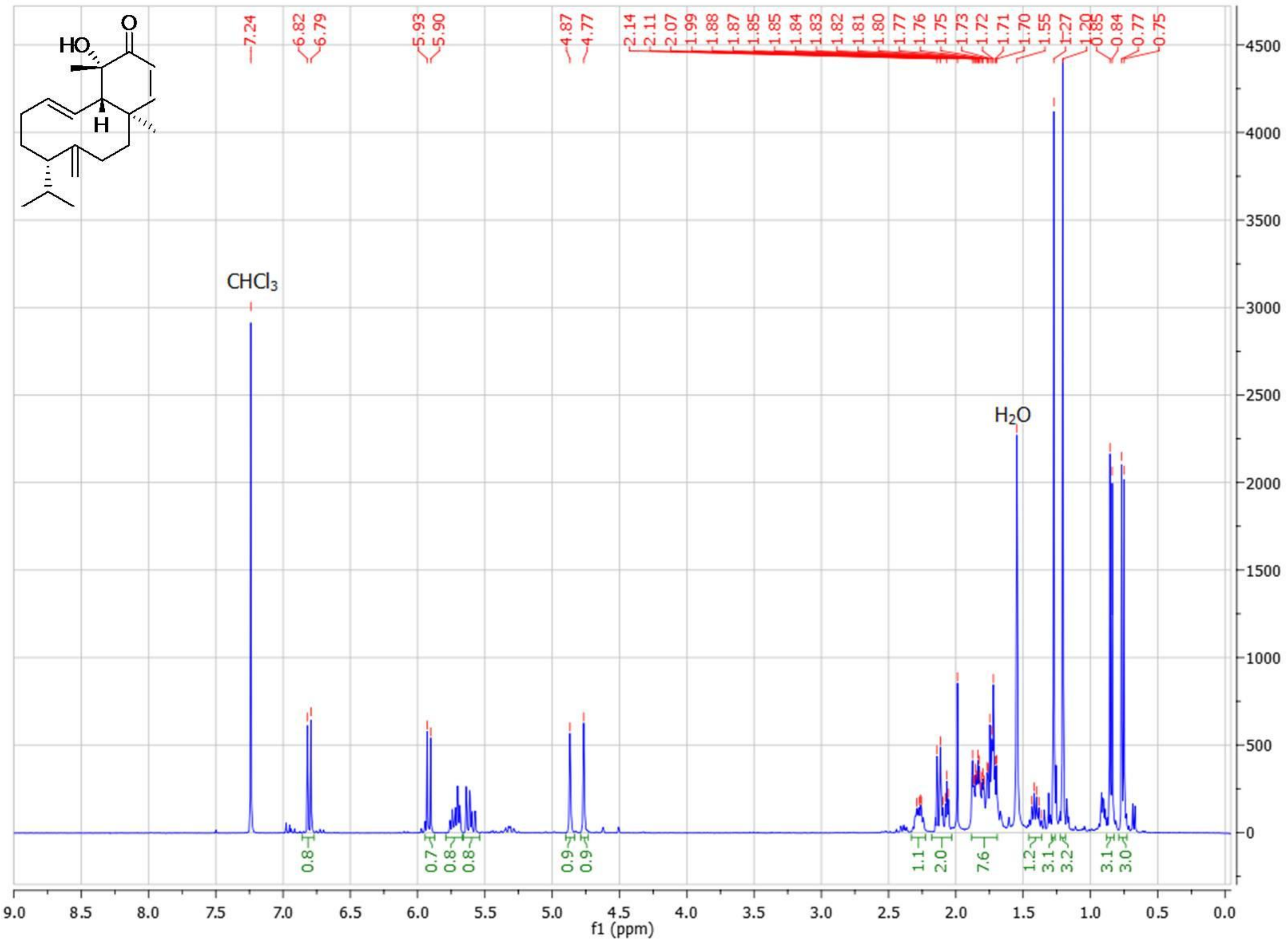

Figure S36. ^{13}C NMR spectrum (50 MHz, $CDCl_3$) of corotrienone (**5**).

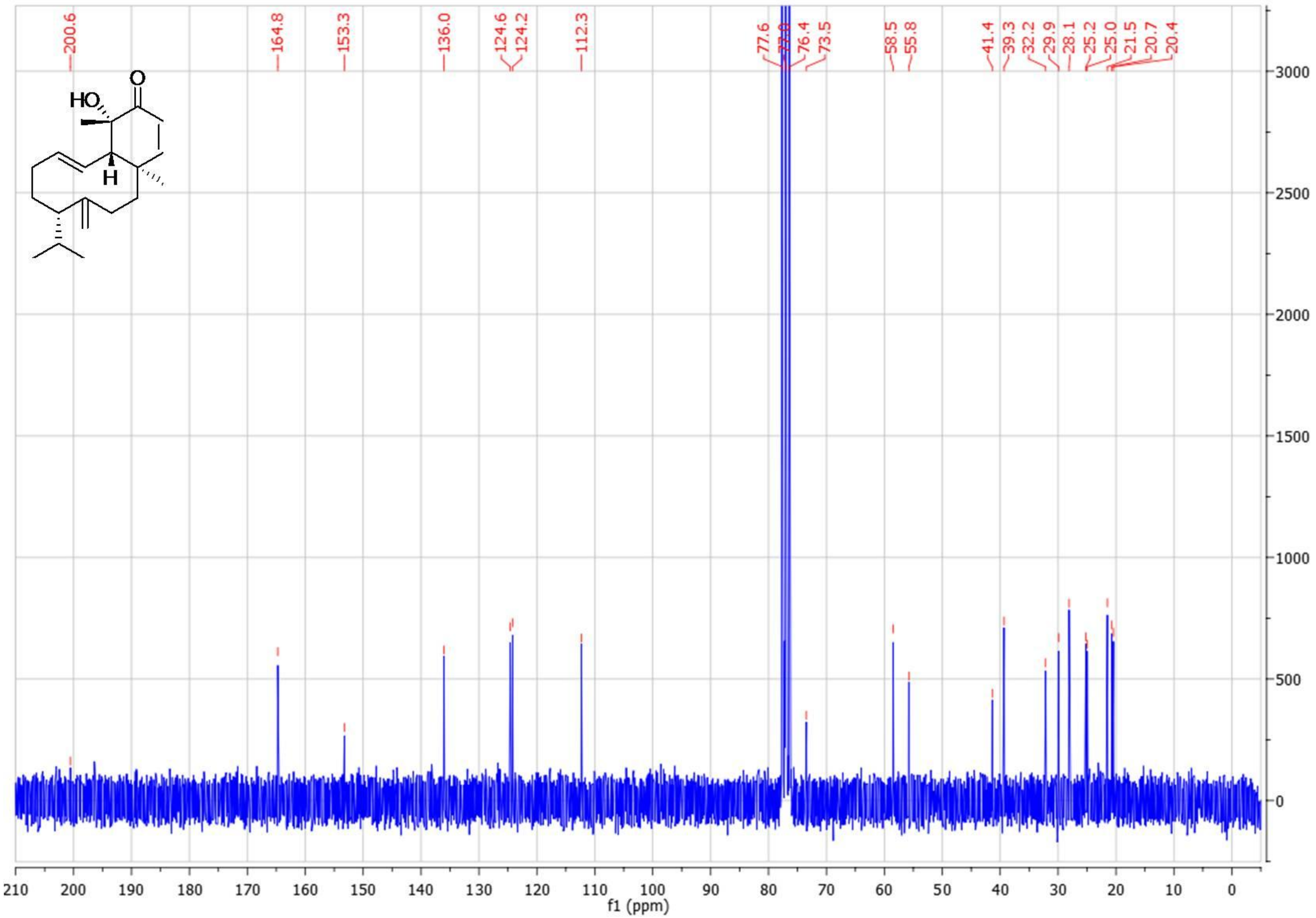

Figure S37. COSY spectrum (400 MHz, $CDCl_3$) of corotrienone (**5**).

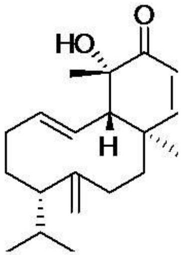

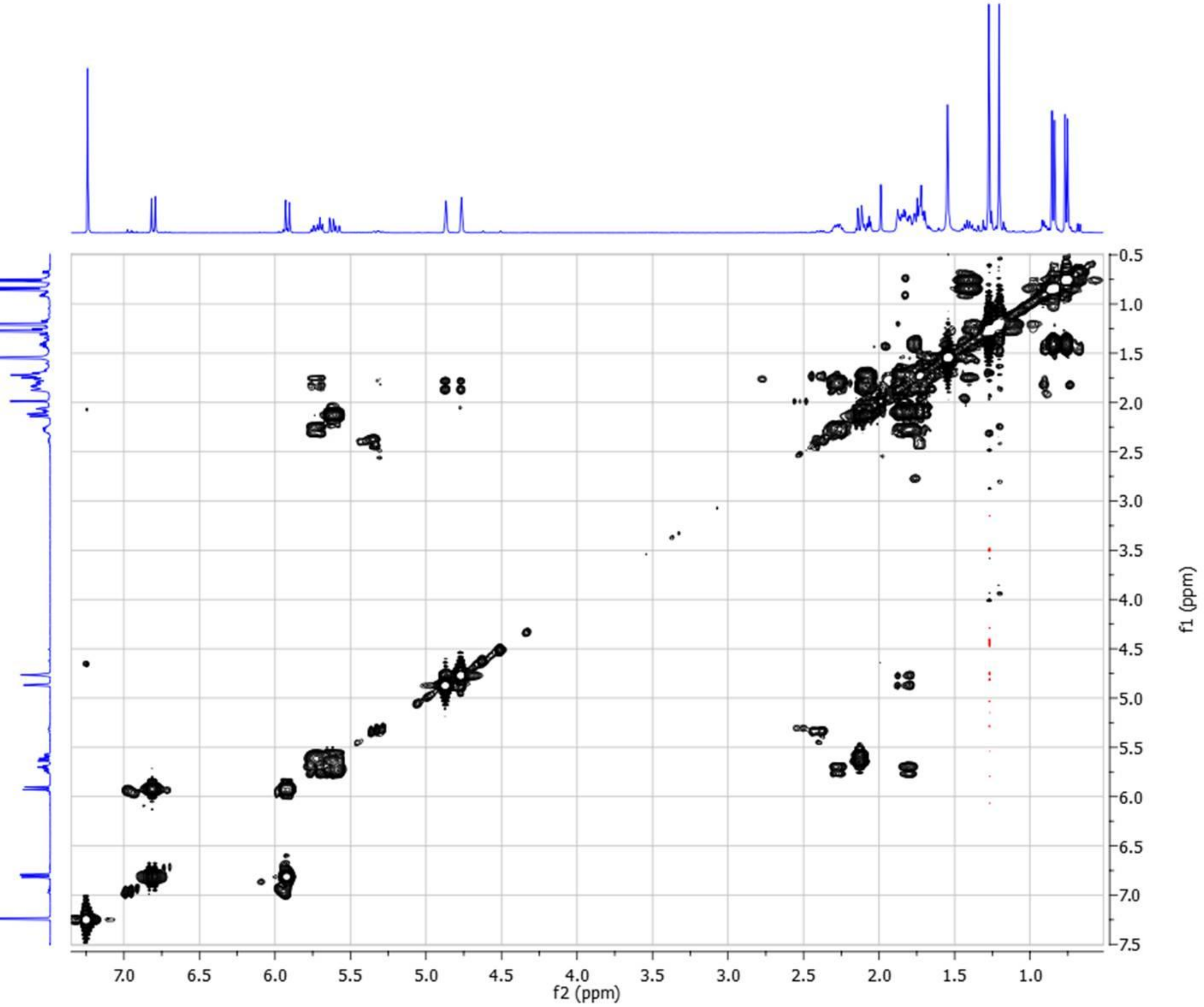

Figure S38. HSQC-DEPT spectrum (400 MHz, $CDCl_3$) of corotrienone (**5**).

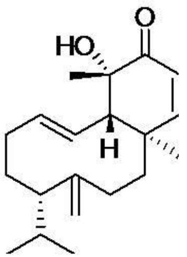

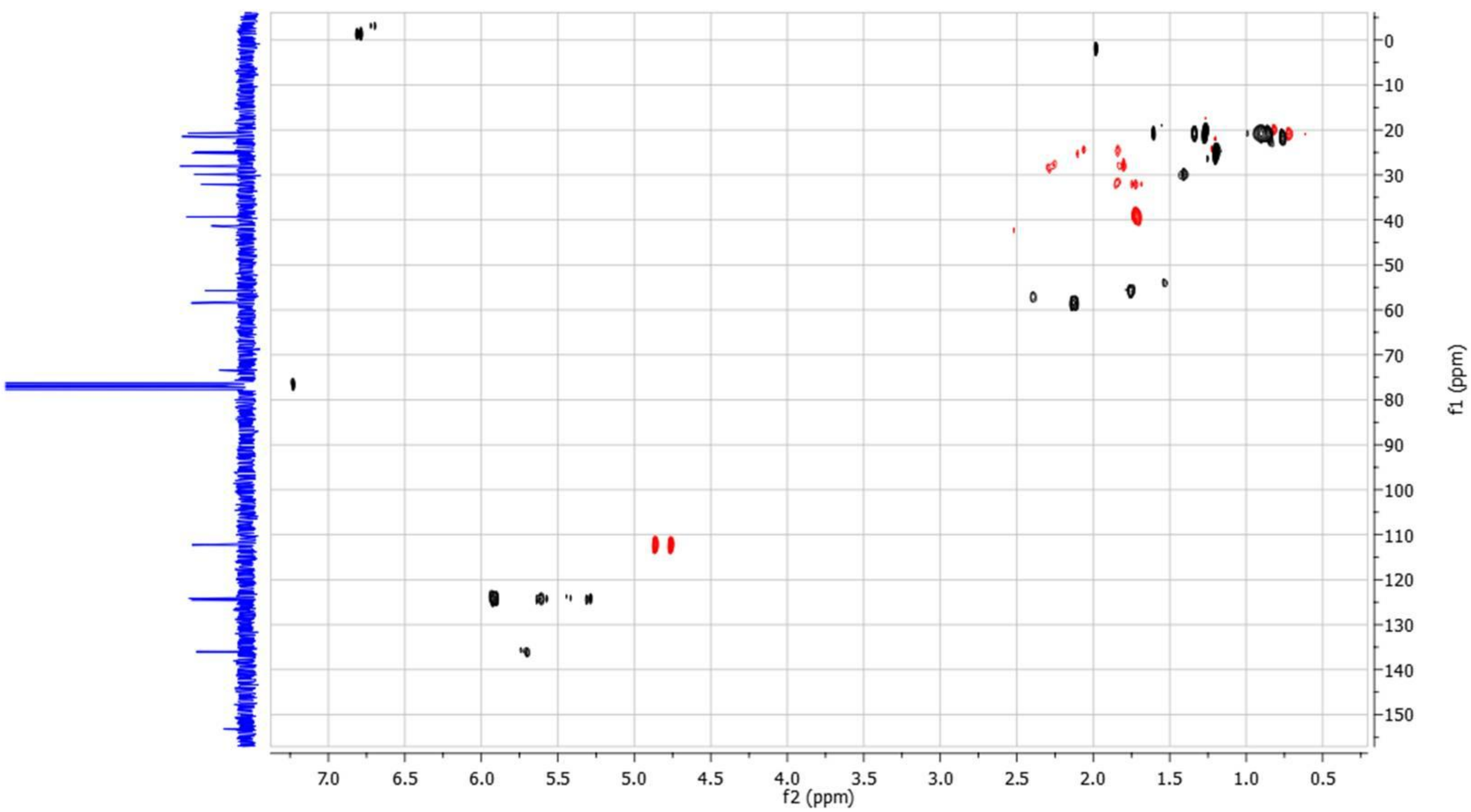

Figure S39. HMBC spectrum (400 MHz, $CDCl_3$) of corotrienone (**5**).

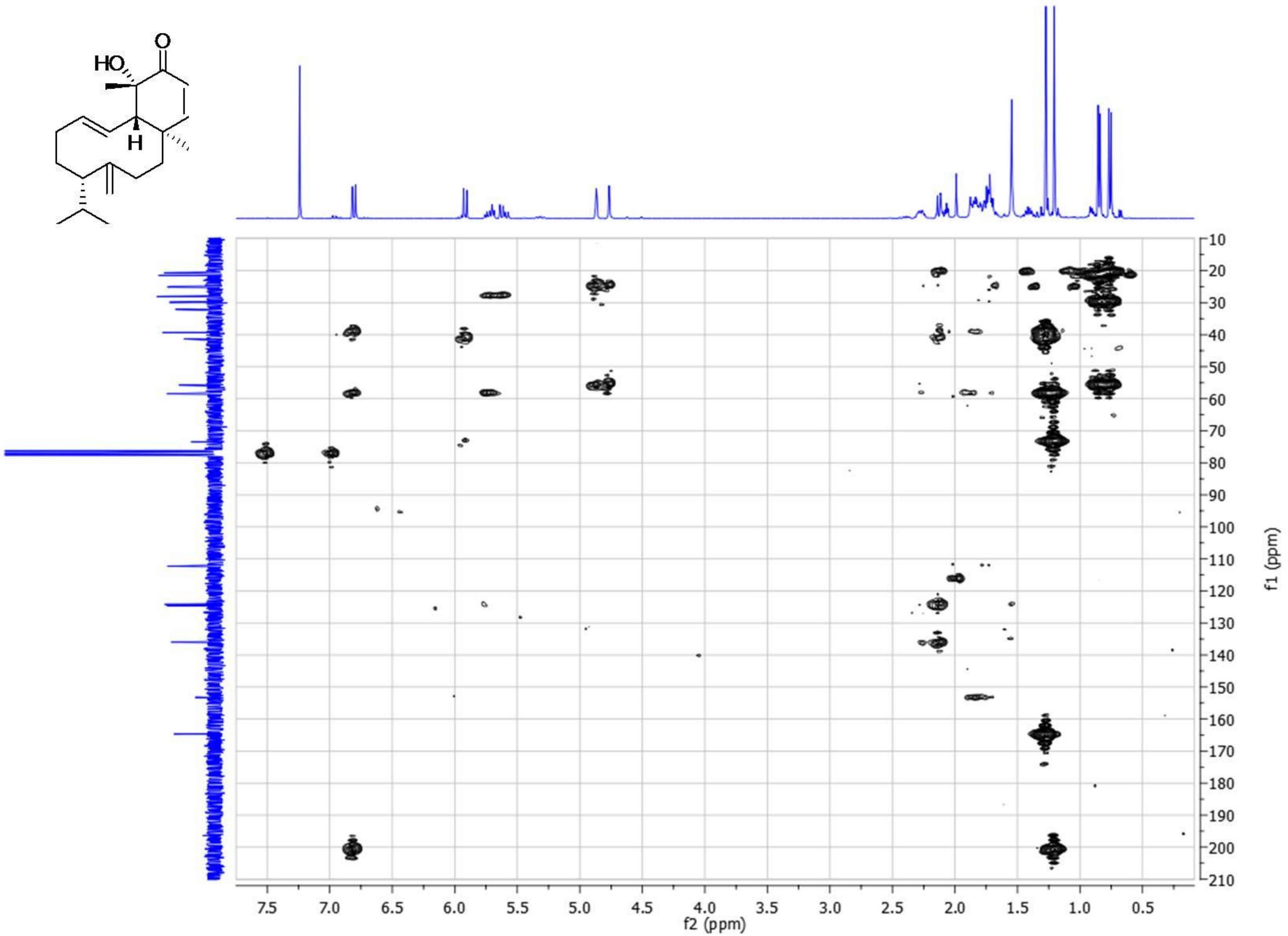

Figure S40. NOESY spectrum (400 MHz, $CDCl_3$) of corotrienone (**5**).

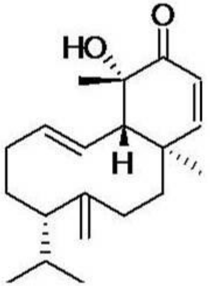

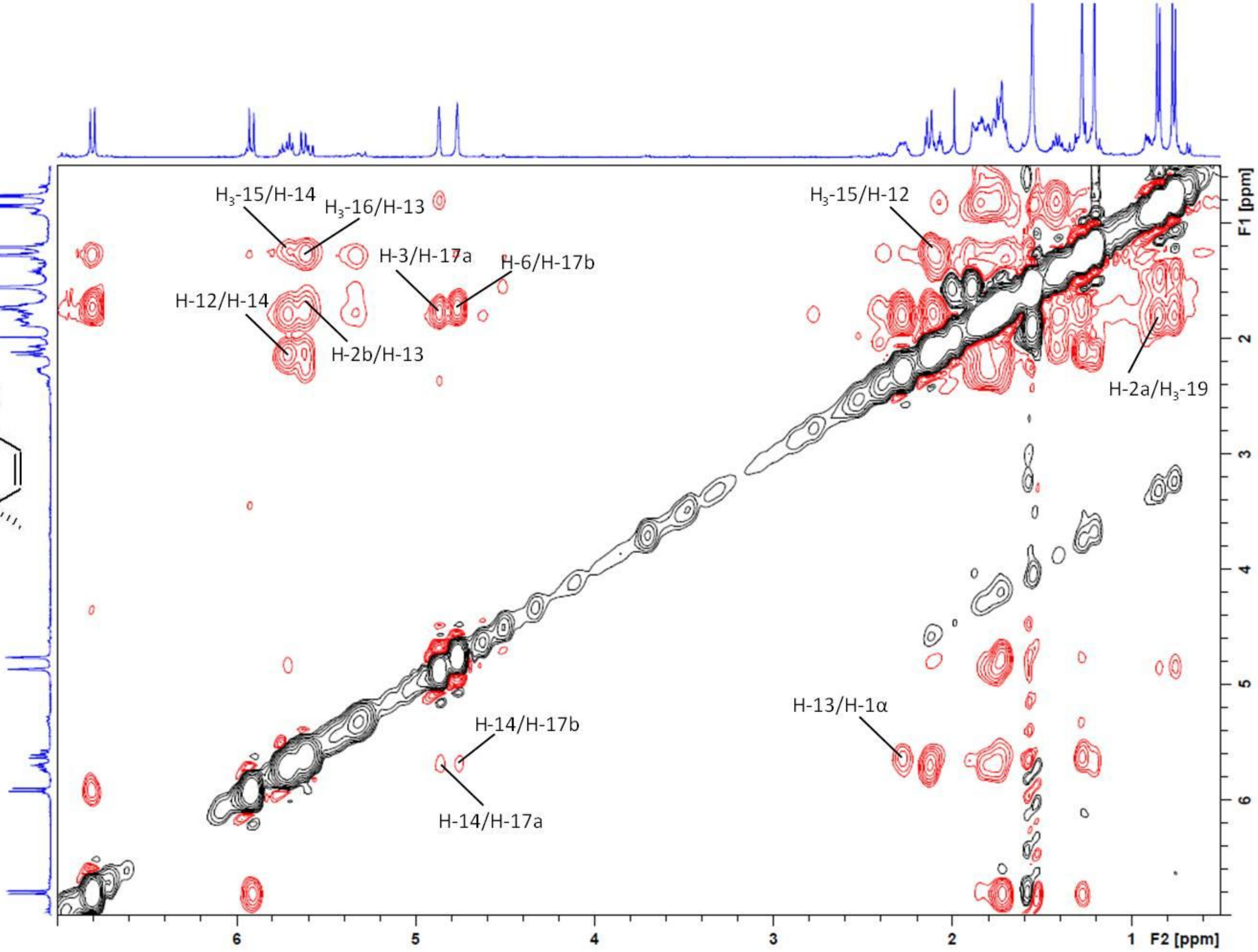

Figure S41. HRMS (ESI+) measurement of corotrienone (**5**).

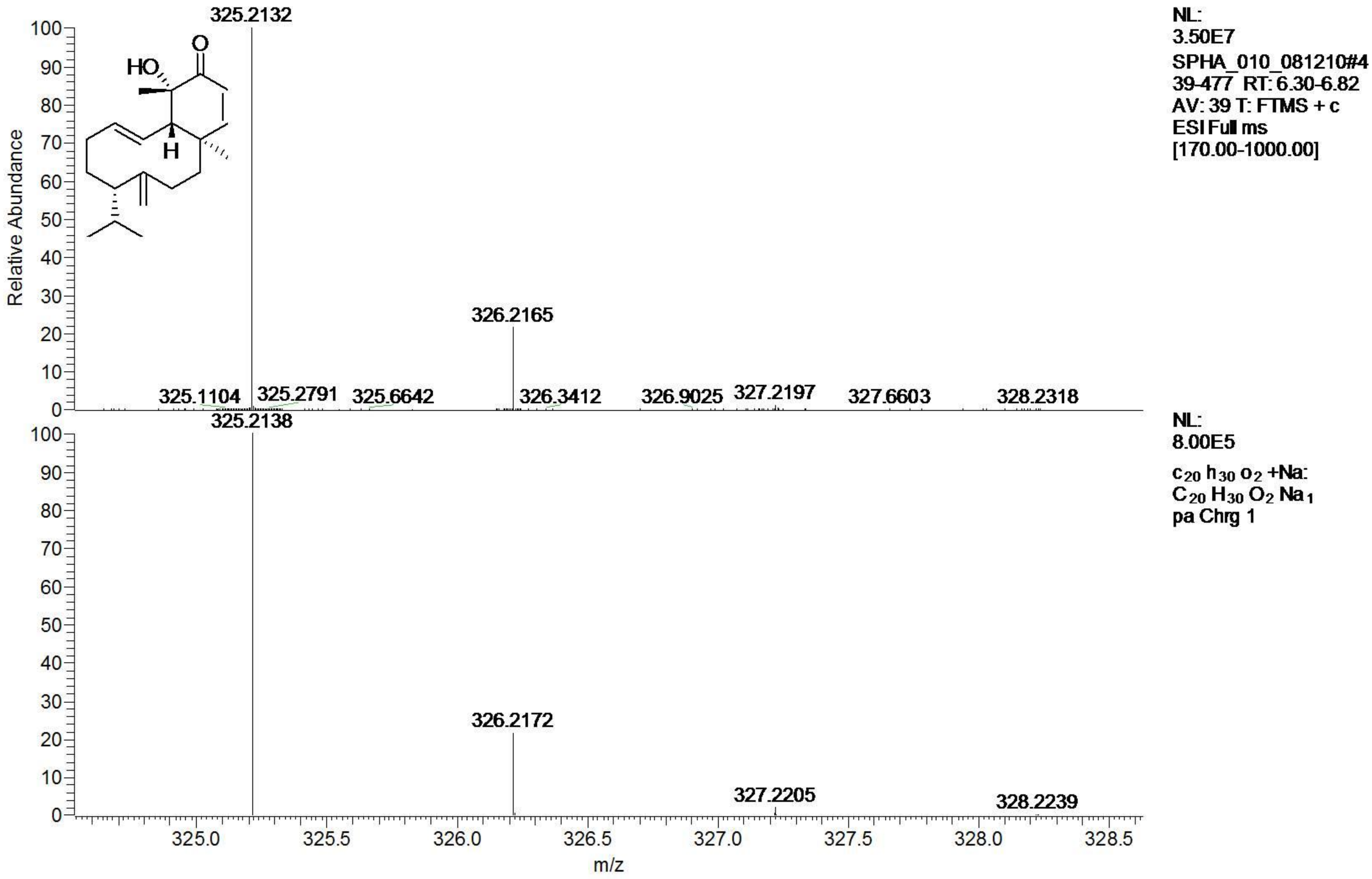

Figure S42. IR spectrum of corotrienone (**5**).

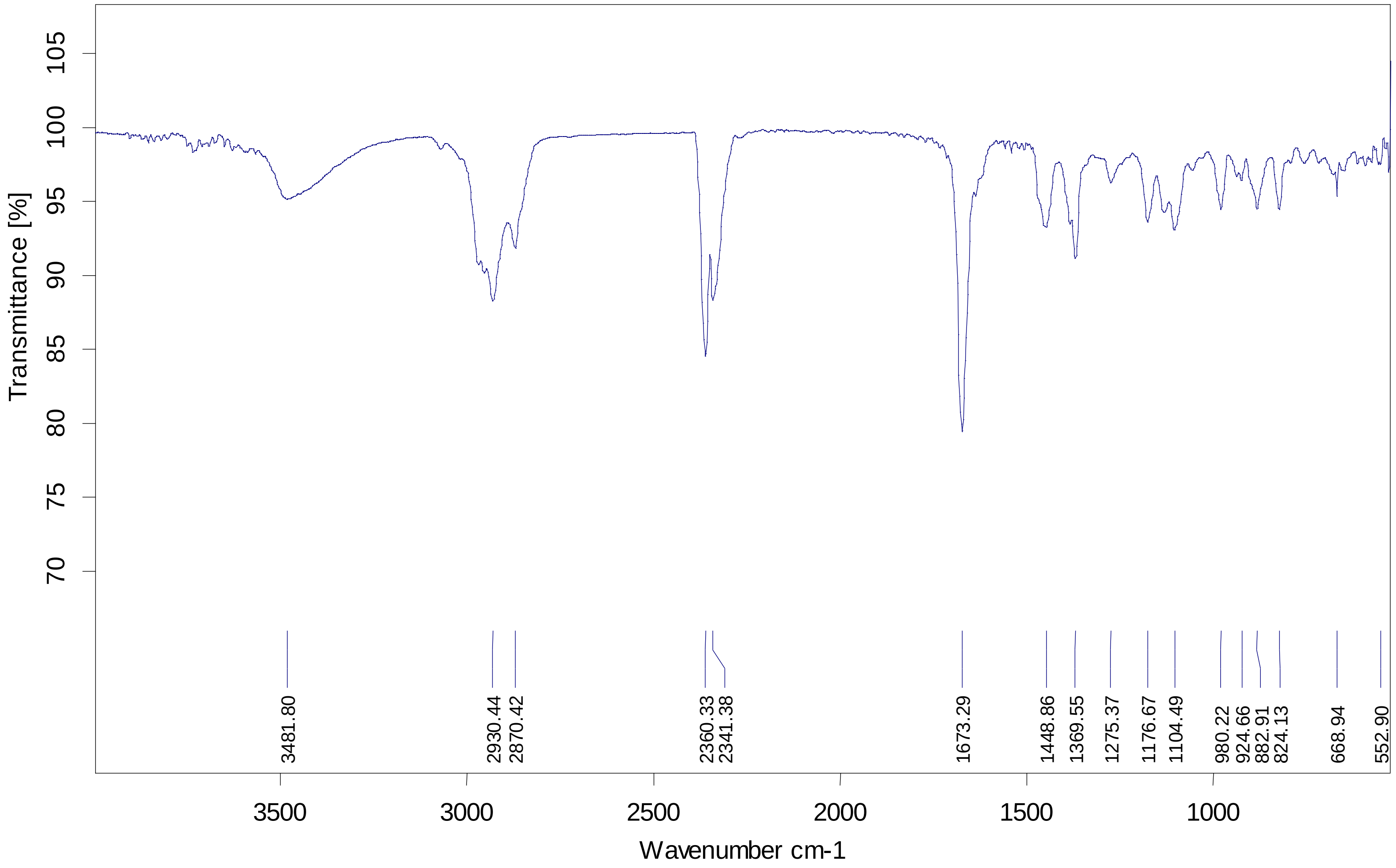

Figure S42. ^{1}H NMR spectrum (400 MHz, $CDCl_3$) of iso-bromocorodienol (**6**).

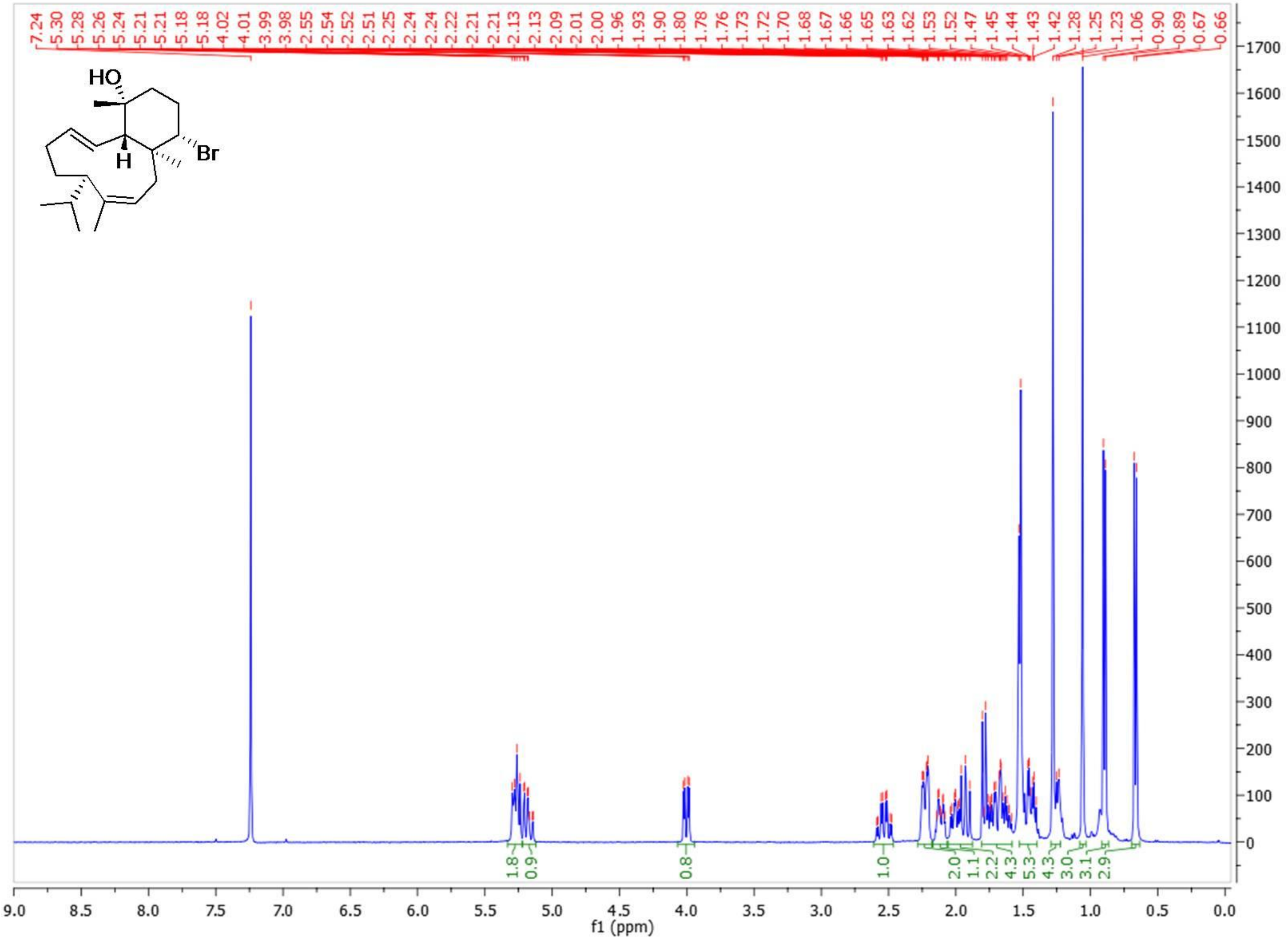

Figure S43. ^{13}C NMR spectrum (50 MHz, CDCl$_3$) of iso-bromocorodienol (**6**).

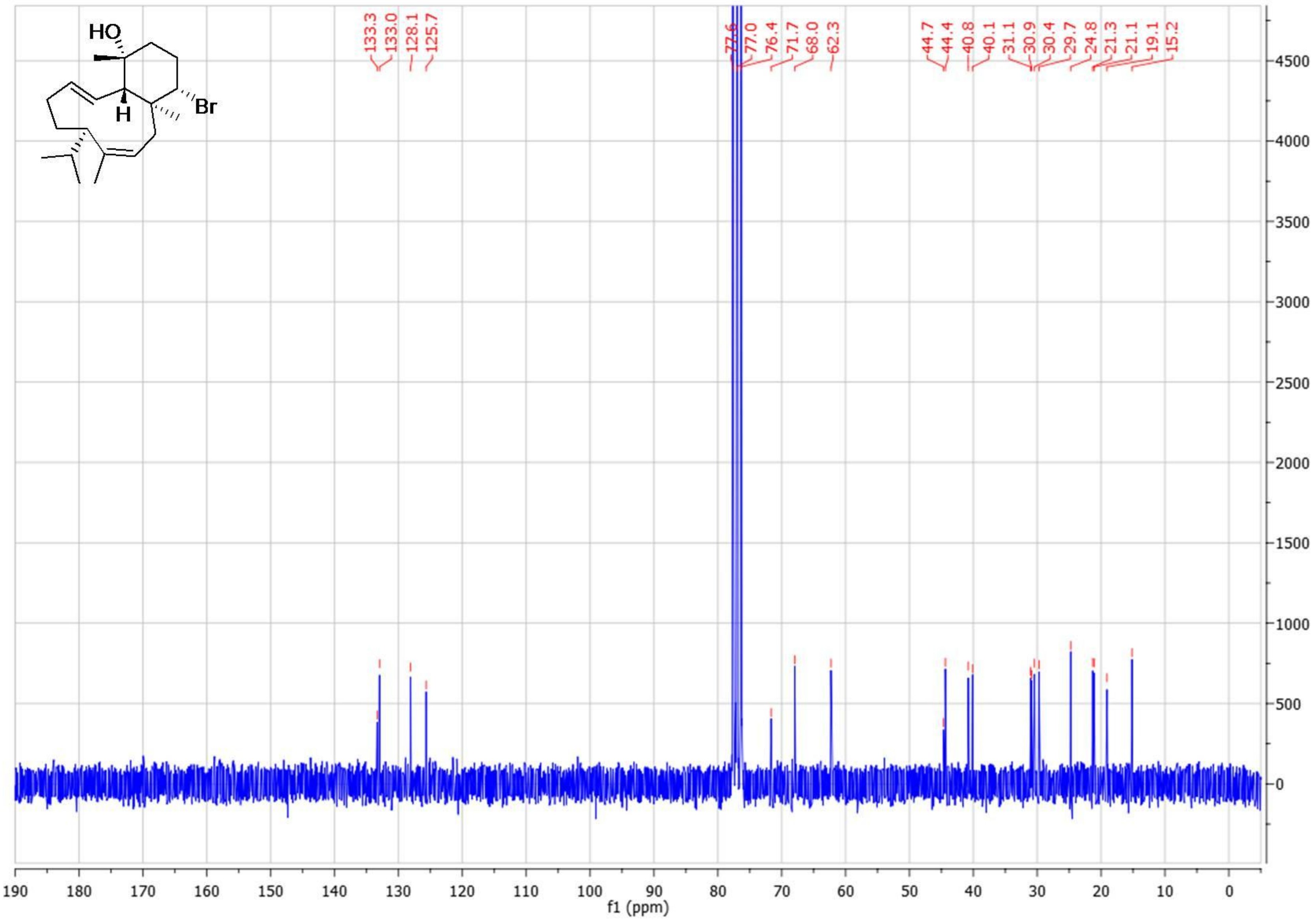

Figure S44. COSY spectrum (400 MHz, $CDCl_3$) of iso-bromocorodienol (**6**).

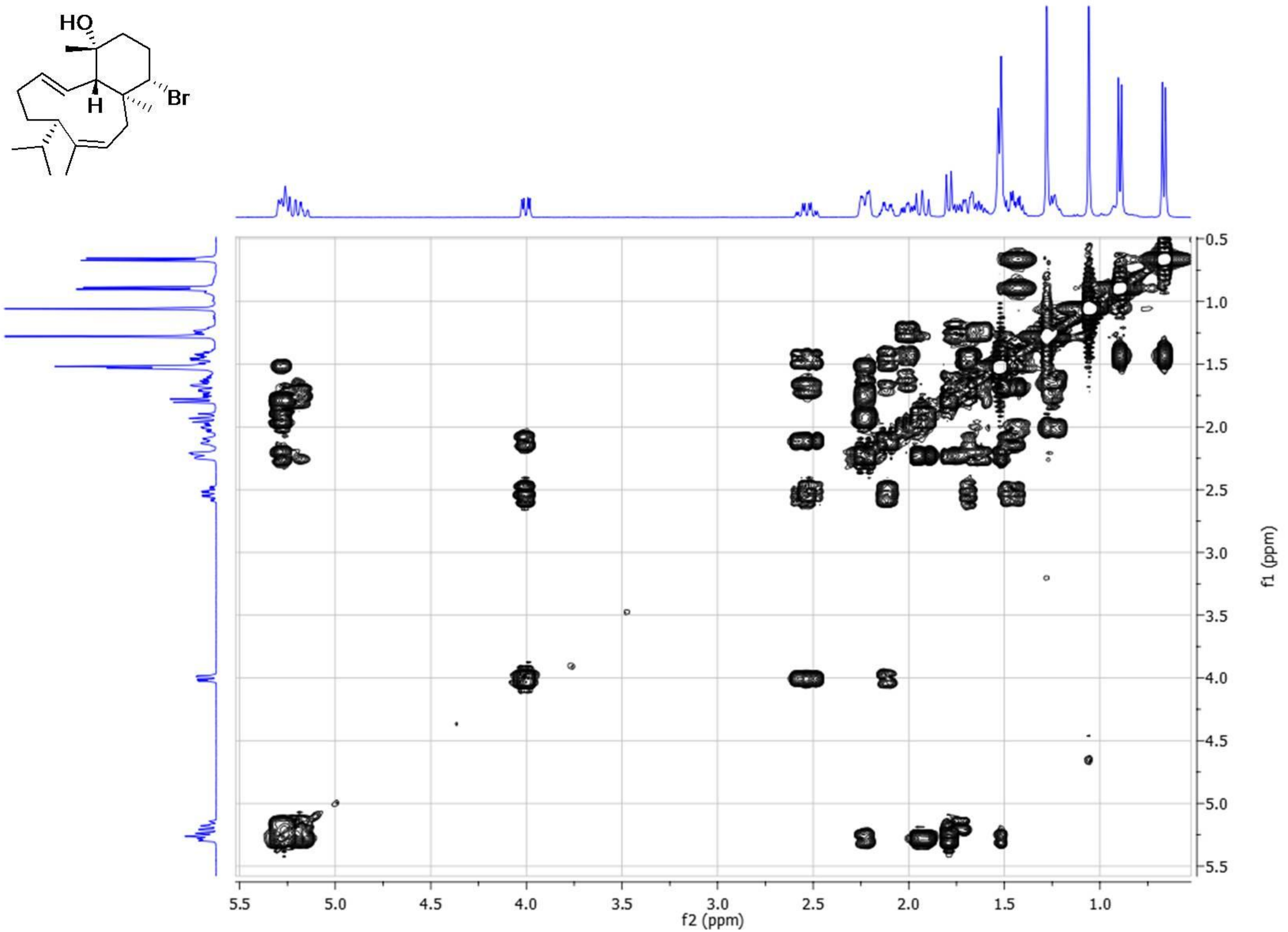

Figure S45. HSQC-DEPT spectrum (400 MHz, $CDCl_3$) of iso-bromocorodienol (**6**).

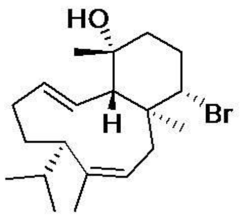

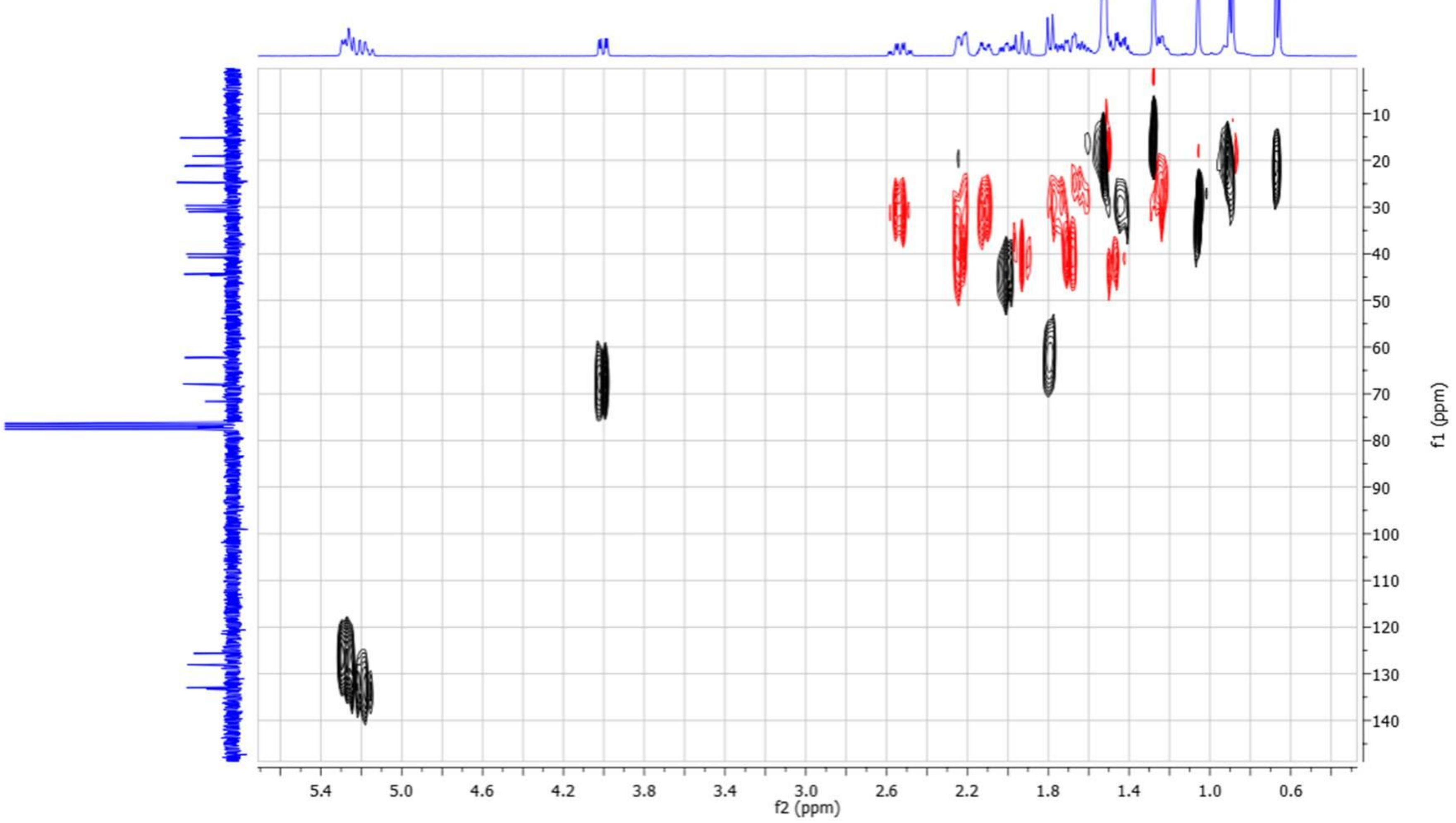

Figure S46. HMBC spectrum (400 MHz, $CDCl_3$) of iso-bromocorodienol (**6**).

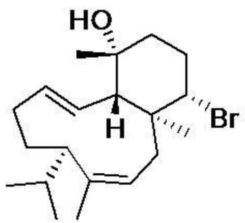

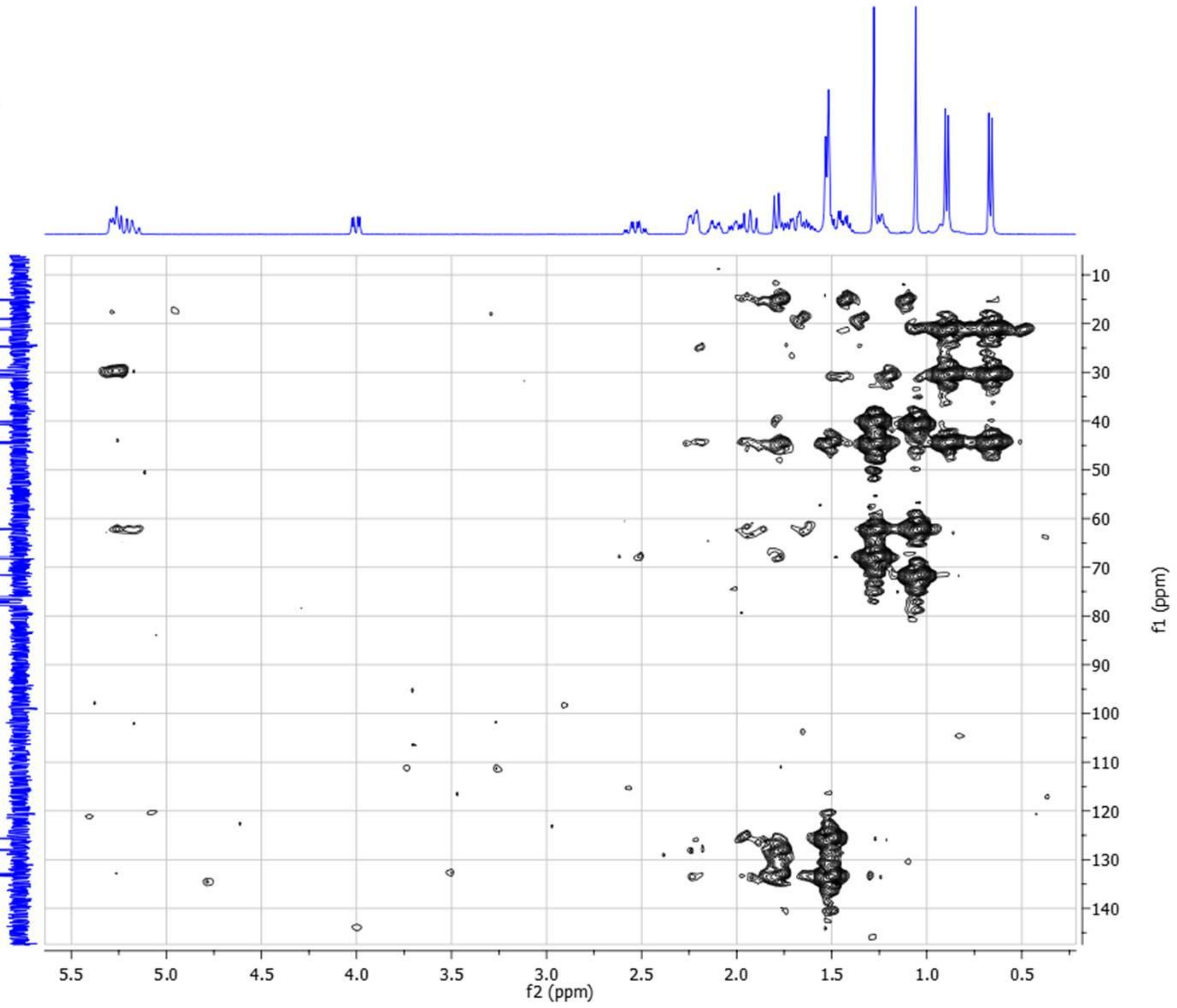

Figure S47. NOESY spectrum (400 MHz, $CDCl_3$) of iso-bromocorodienol (**6**).

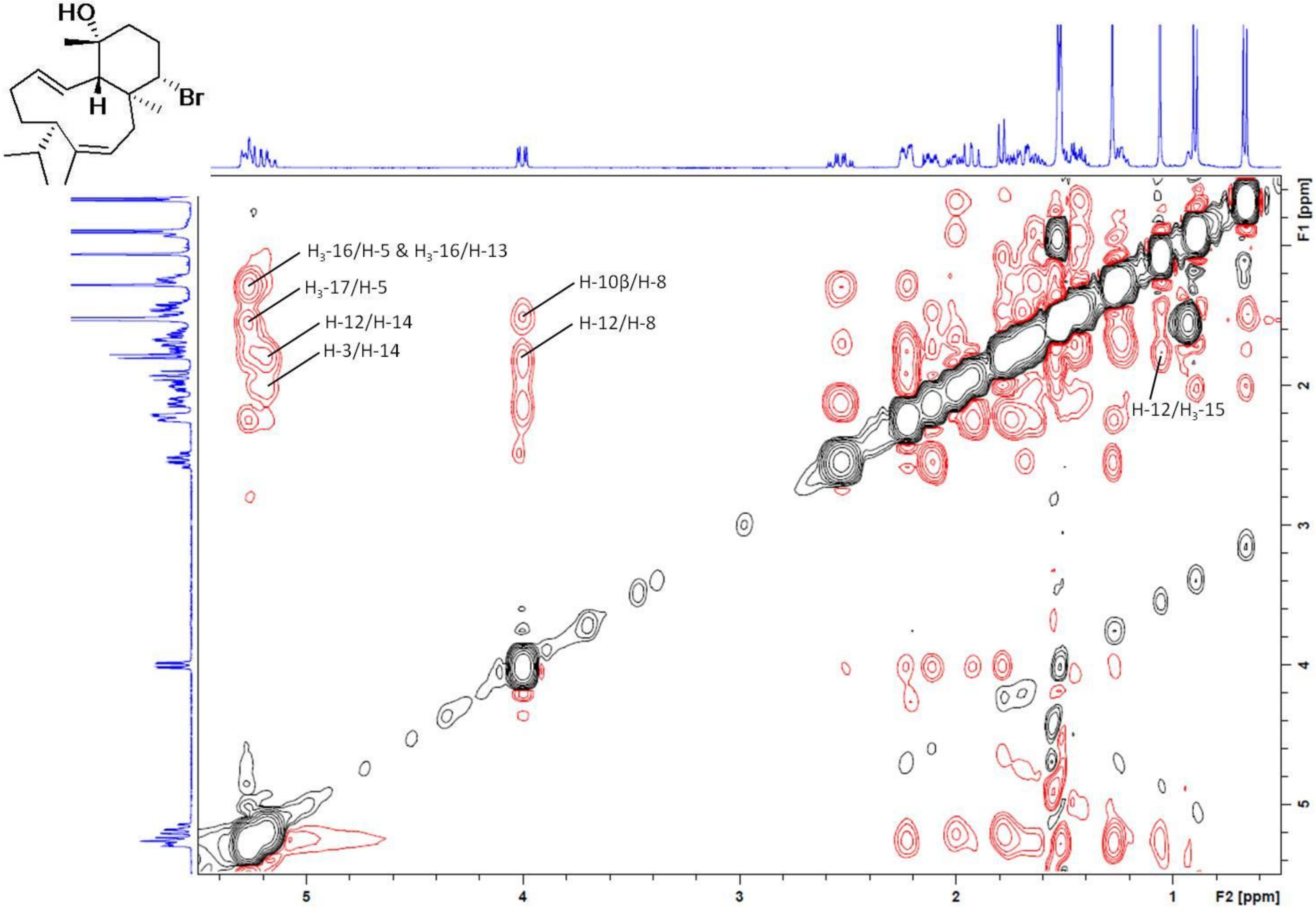

Figure S48. 1D NOE spectrum (400 MHz, $CDCl_3$), excitation of H-6β of iso-bromocorodienol (**6**).

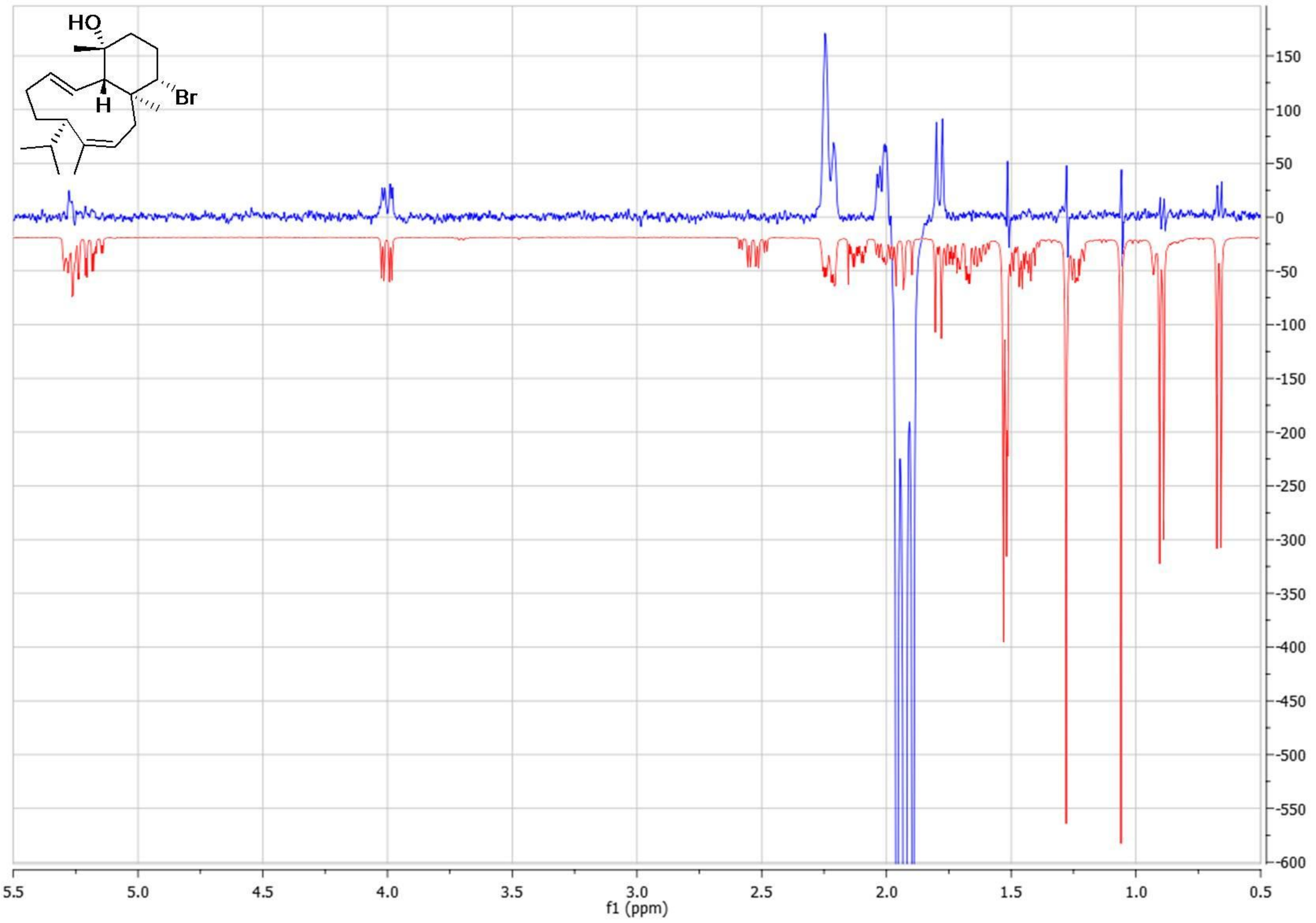

Figure S49. HRMS (ESI+) measurement of iso-bromocorodienol (**6**).

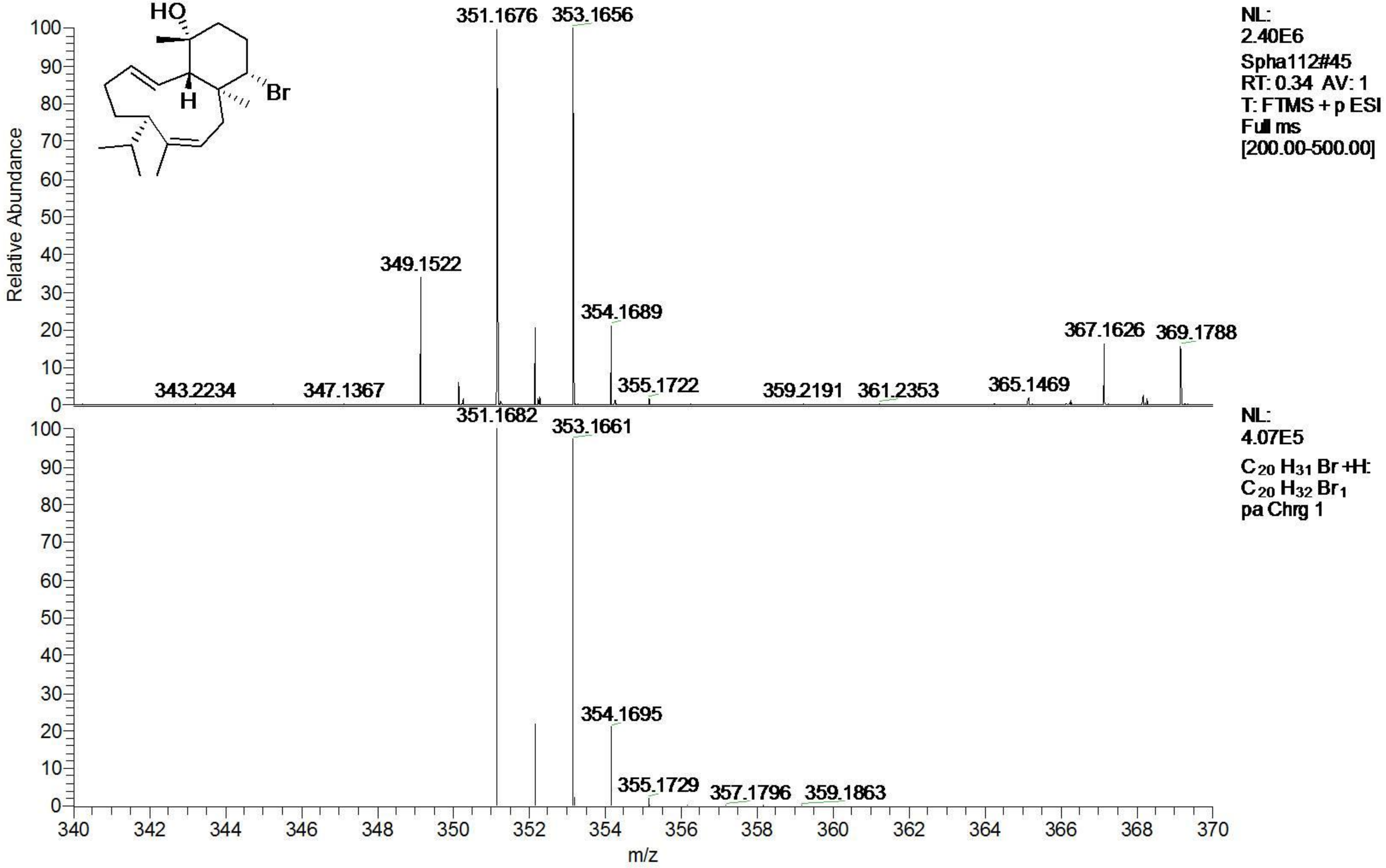

Figure S50. IR spectrum of iso-bromocorodienol (**6**).

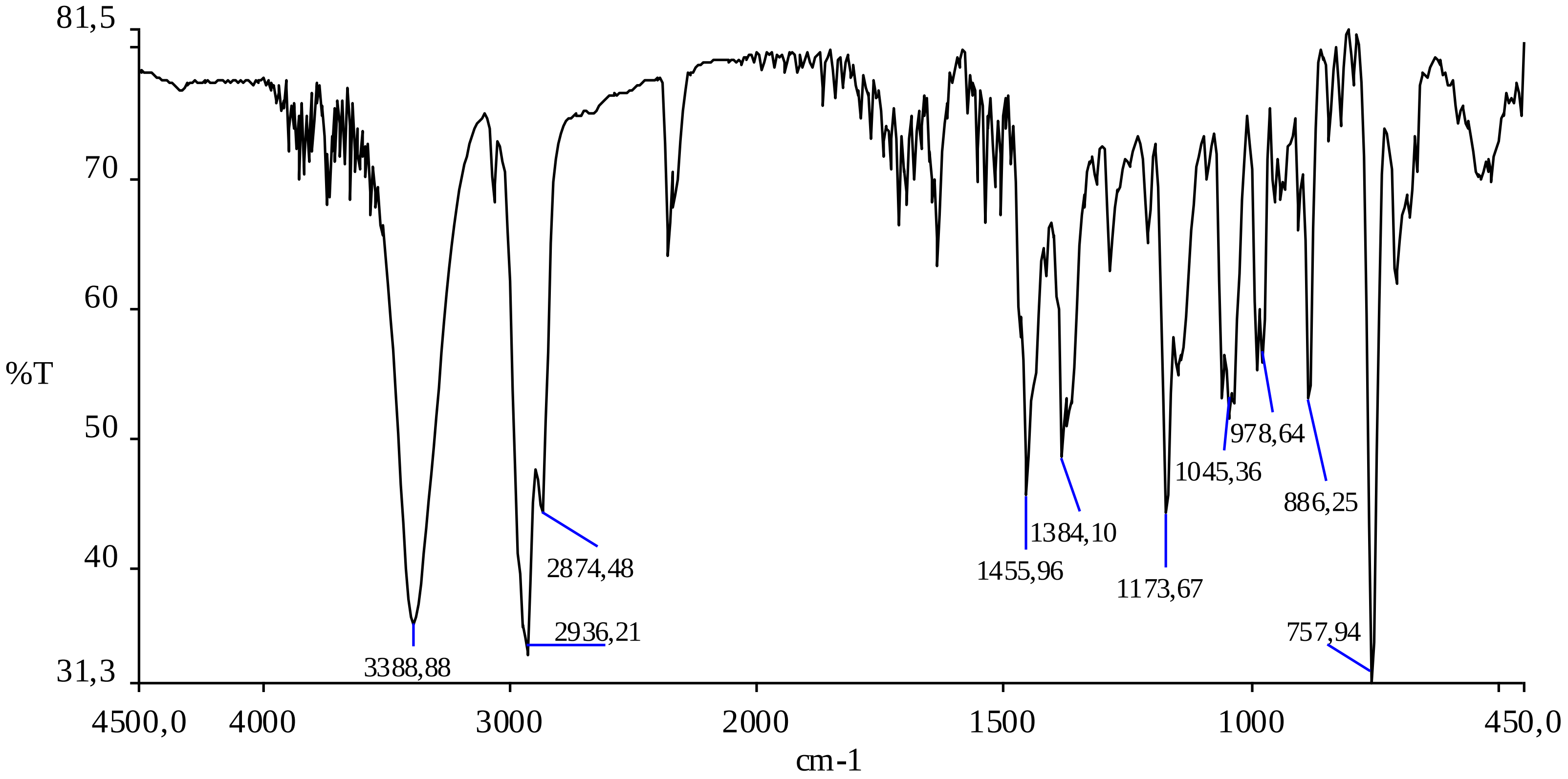

Figure S51. ^{1}H NMR spectrum (400 MHz, $CDCl_3$) of debromosphaerol (**7**).

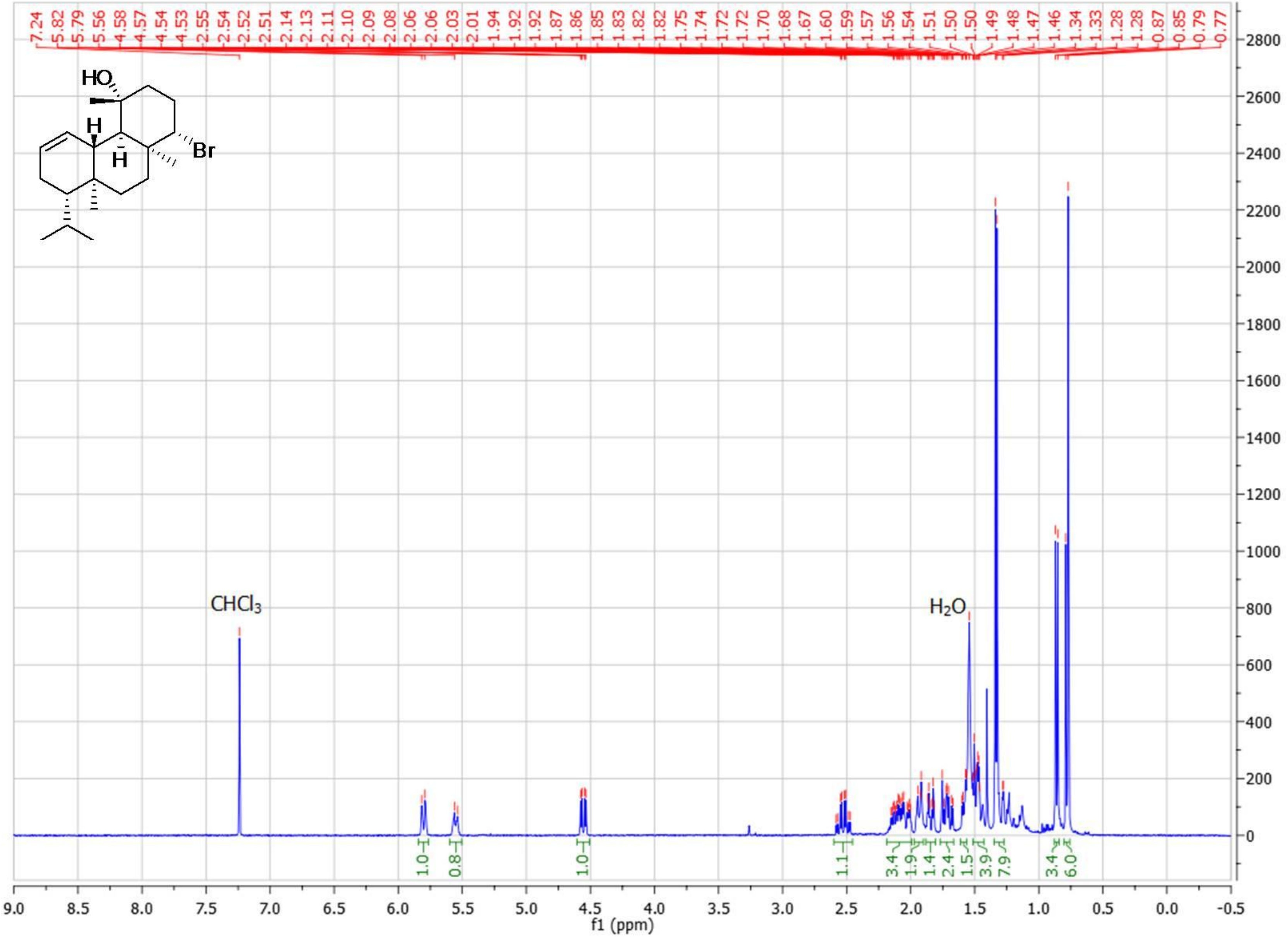

Figure S52. ^{13}C NMR spectrum (50 MHz, $CDCl_3$) of debromosphaerol (**7**).

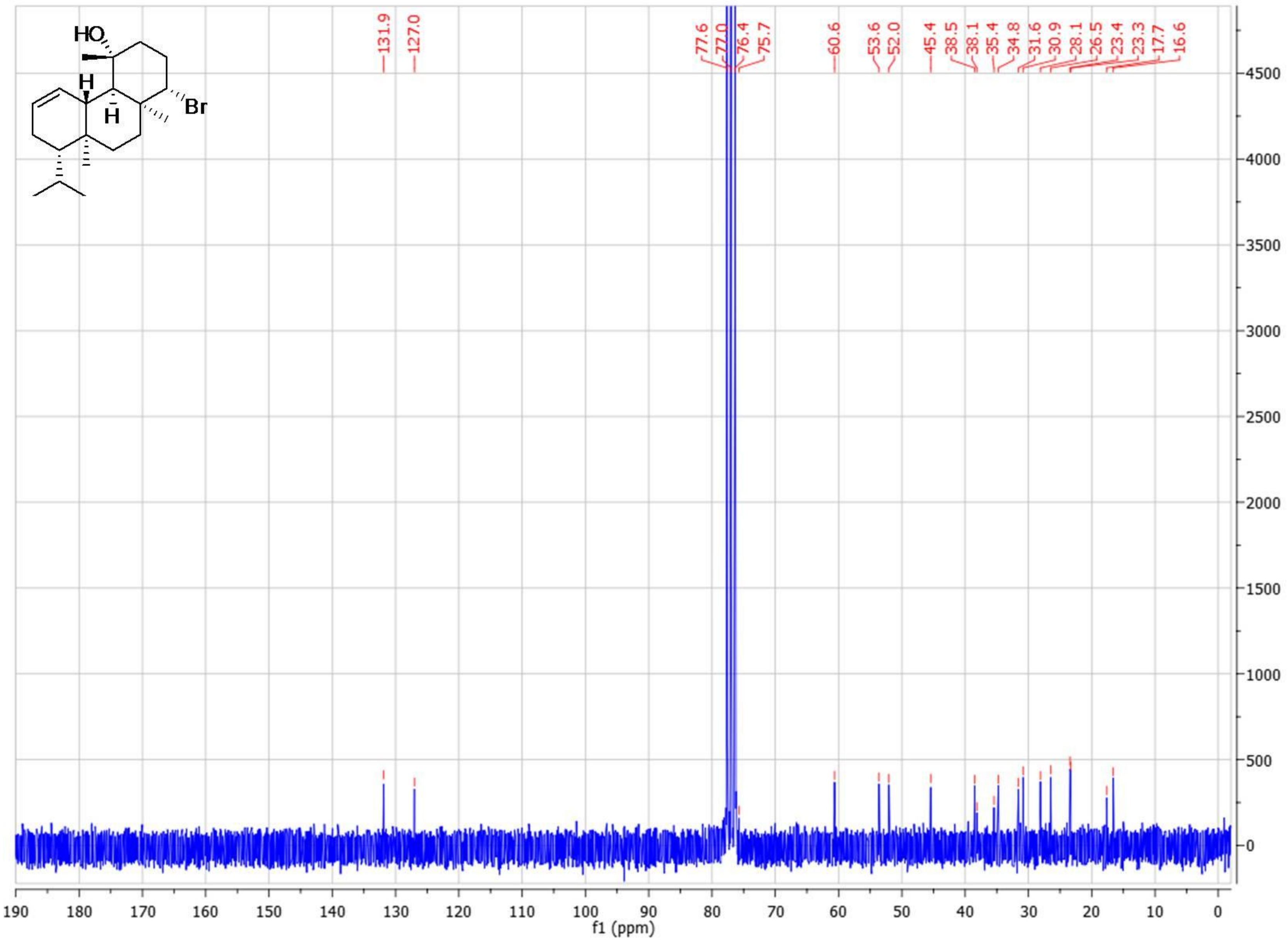

Figure S53. DEPT-135 spectrum (50 MHz, $CDCl_3$) of debromosphaerol (**7**).

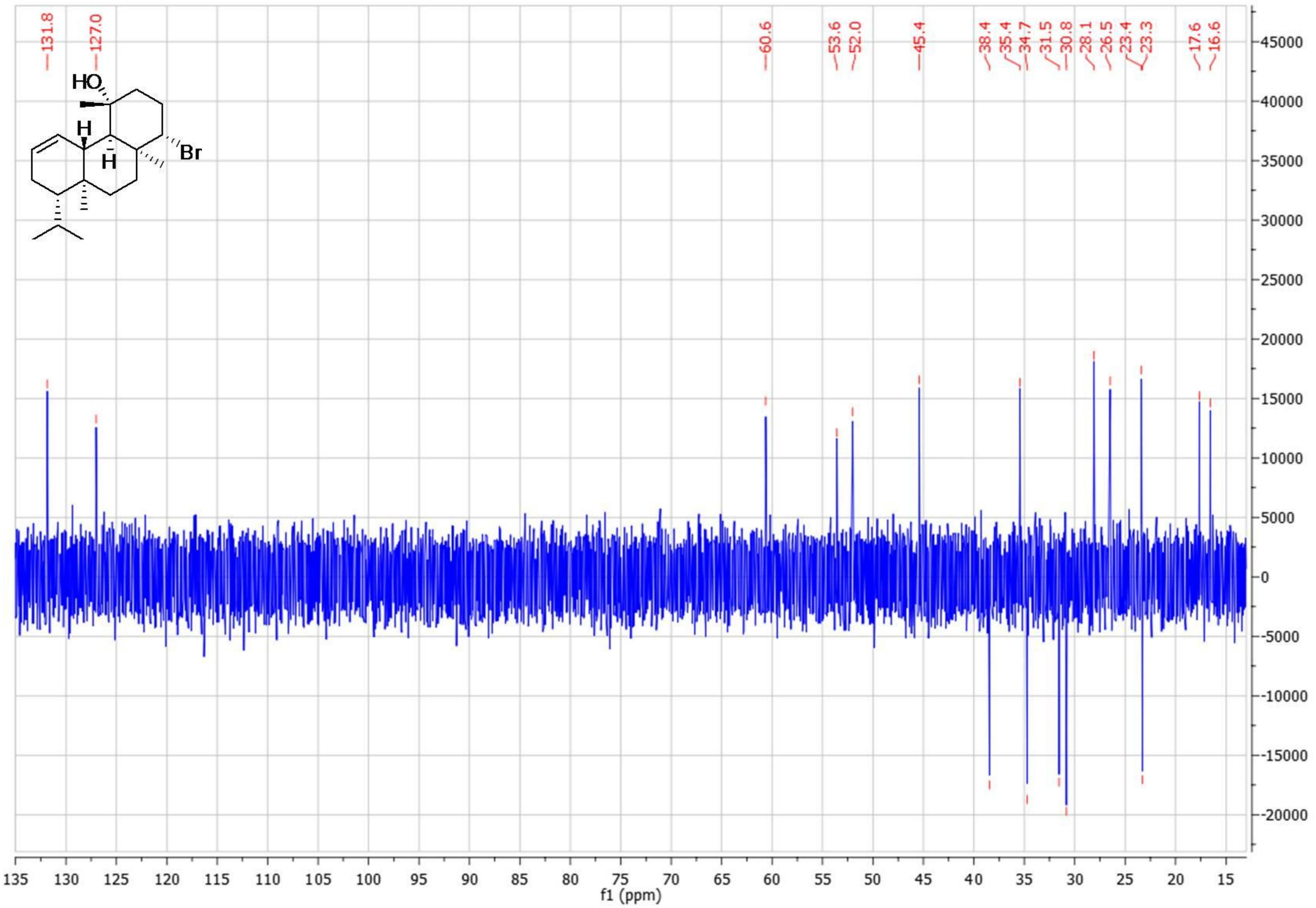

Figure S54. COSY spectrum (400 MHz, $CDCl_3$) of debromosphaerol (**7**).

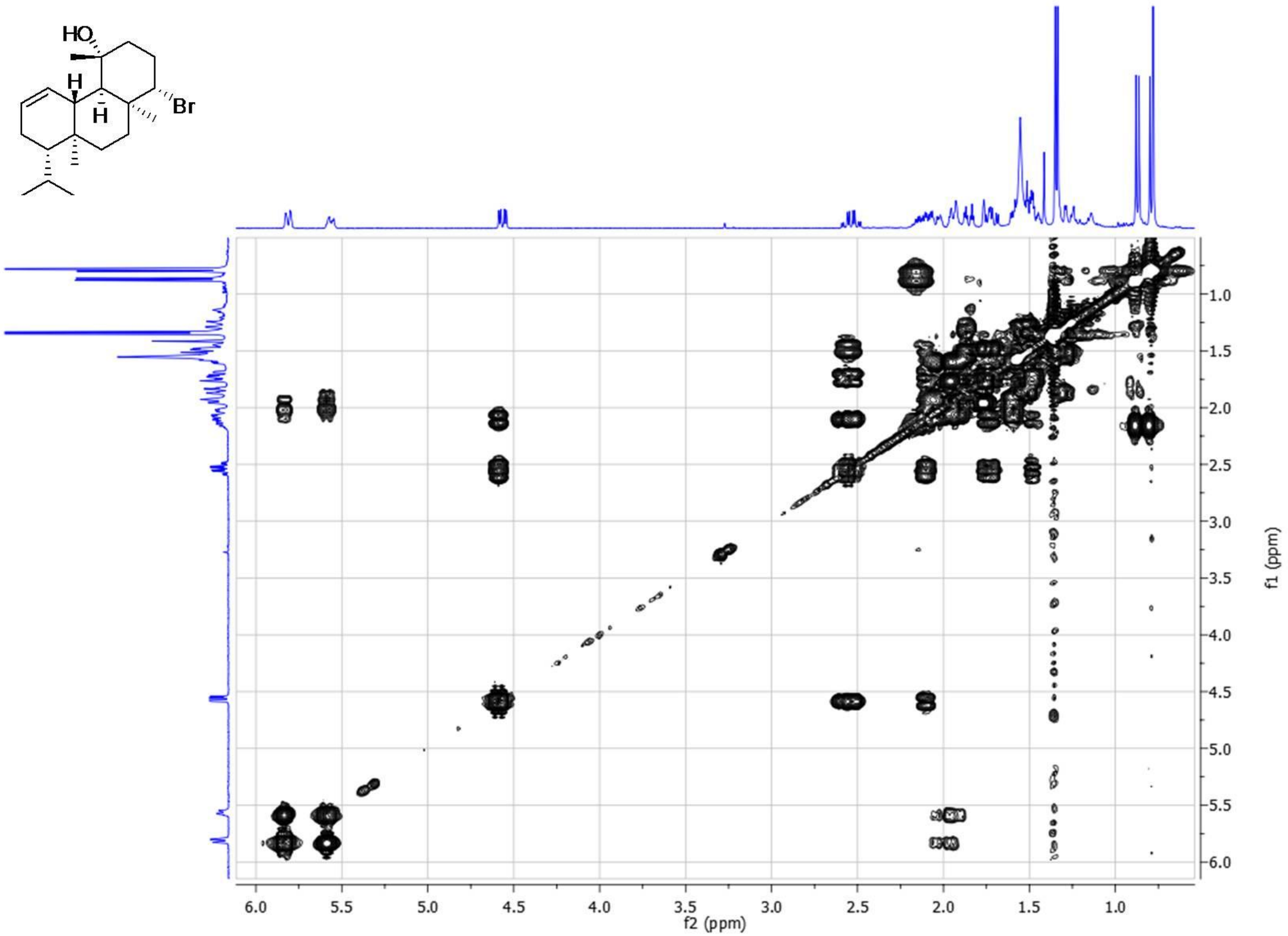

Figure S55. HSQC-DEPT spectrum (400 MHz, $CDCl_3$) of debromosphaerol (**7**).

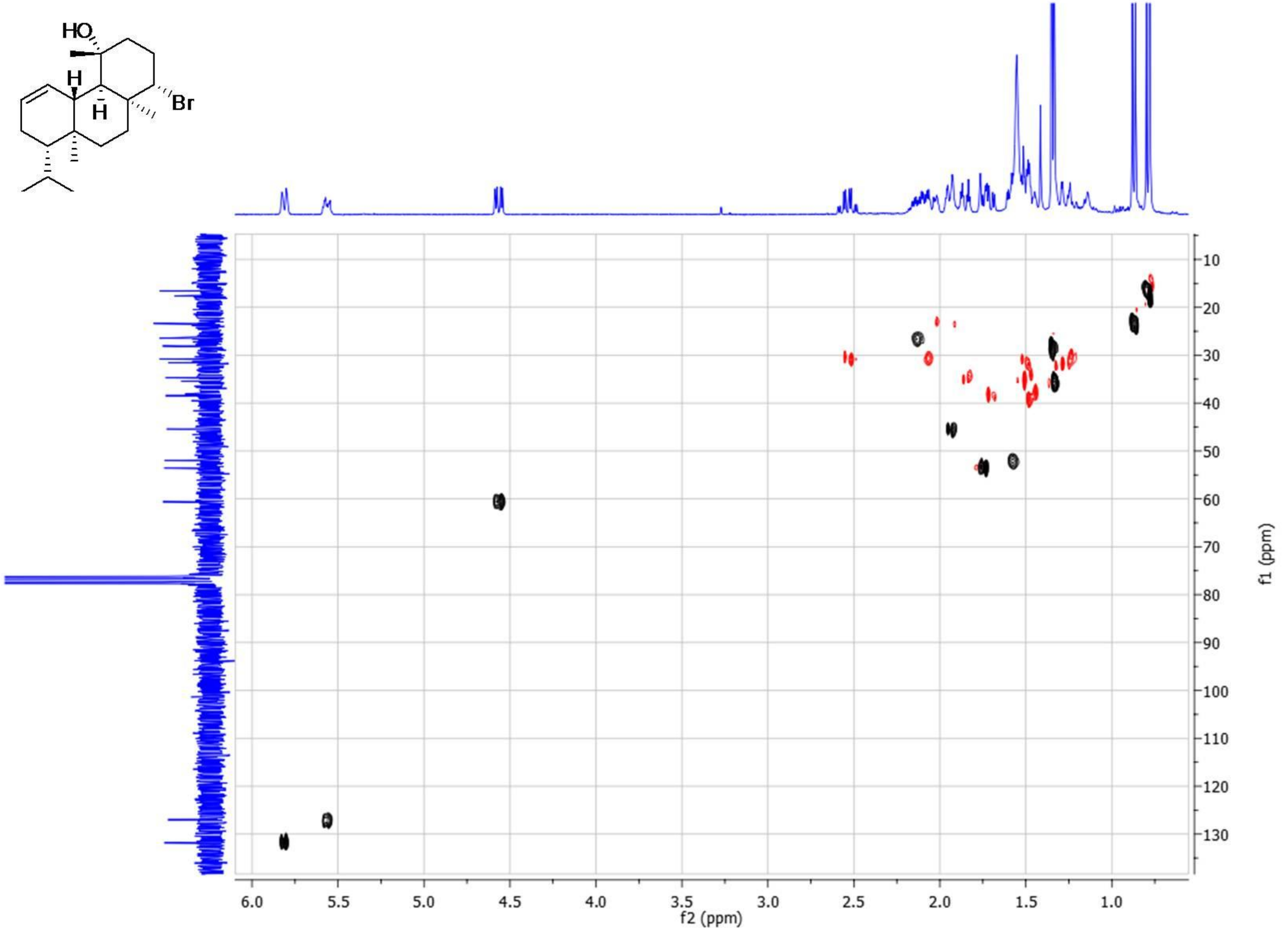

Figure S56. HMBC spectrum (400 MHz, $CDCl_3$) of debromosphaerol (**7**).

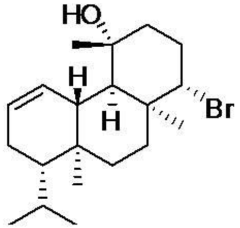

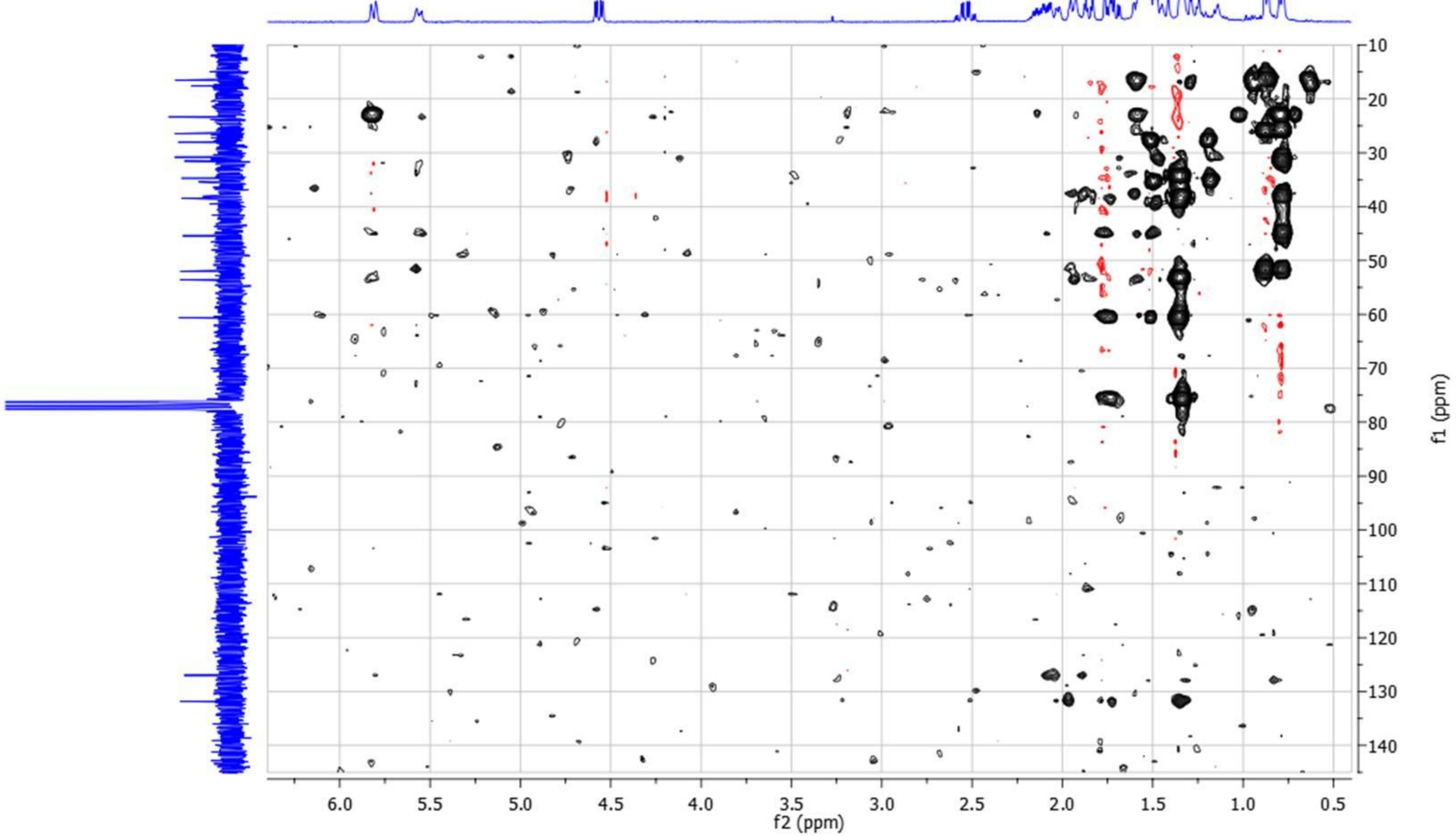

Figure S57. NOESY spectrum (400 MHz, $CDCl_3$) of debromosphaerol (**7**).

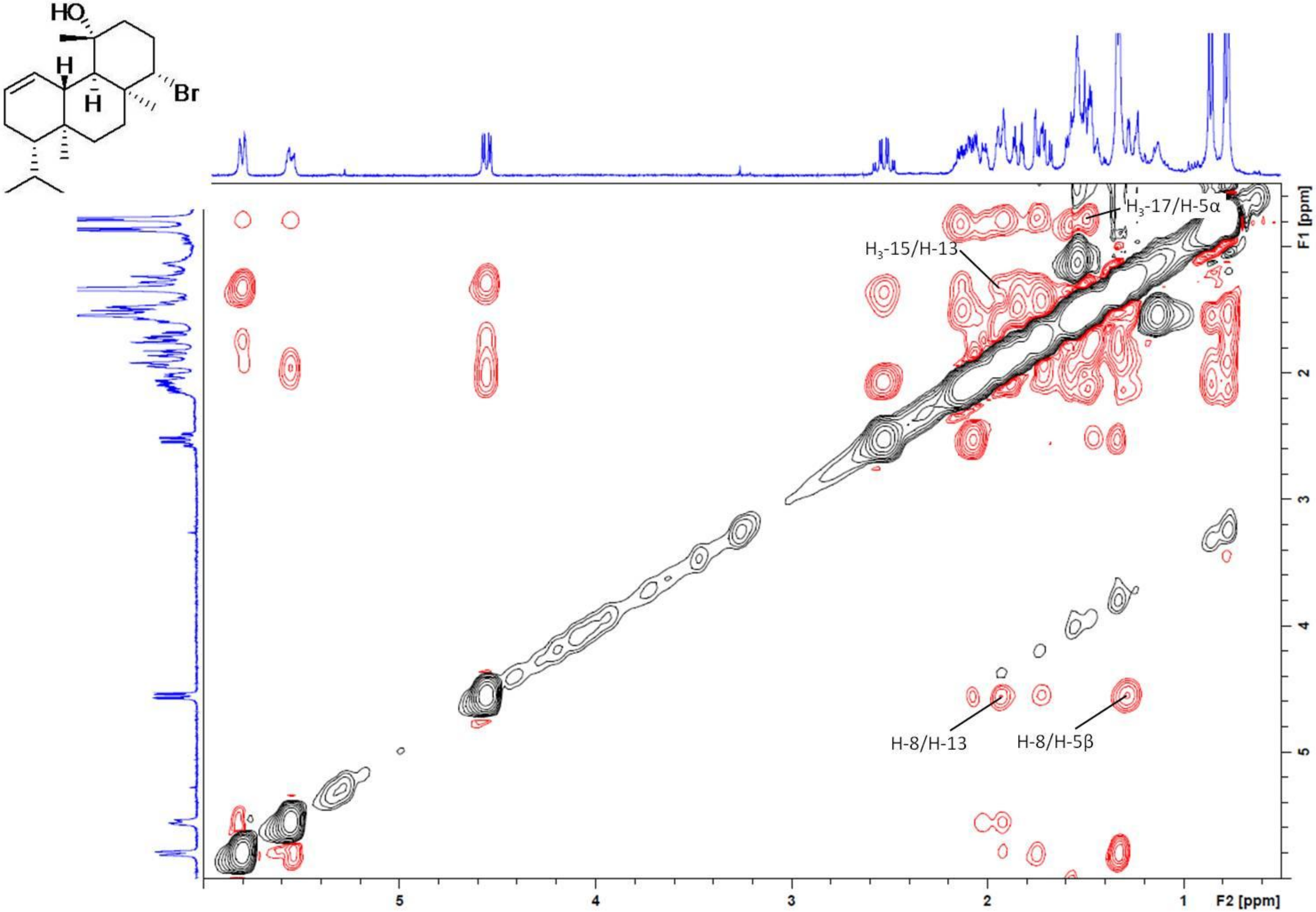

Figure S58. 1D NOE spectrum (400 MHz, $CDCl_3$), excitation of H-3 of debromosphaerol (**7**).

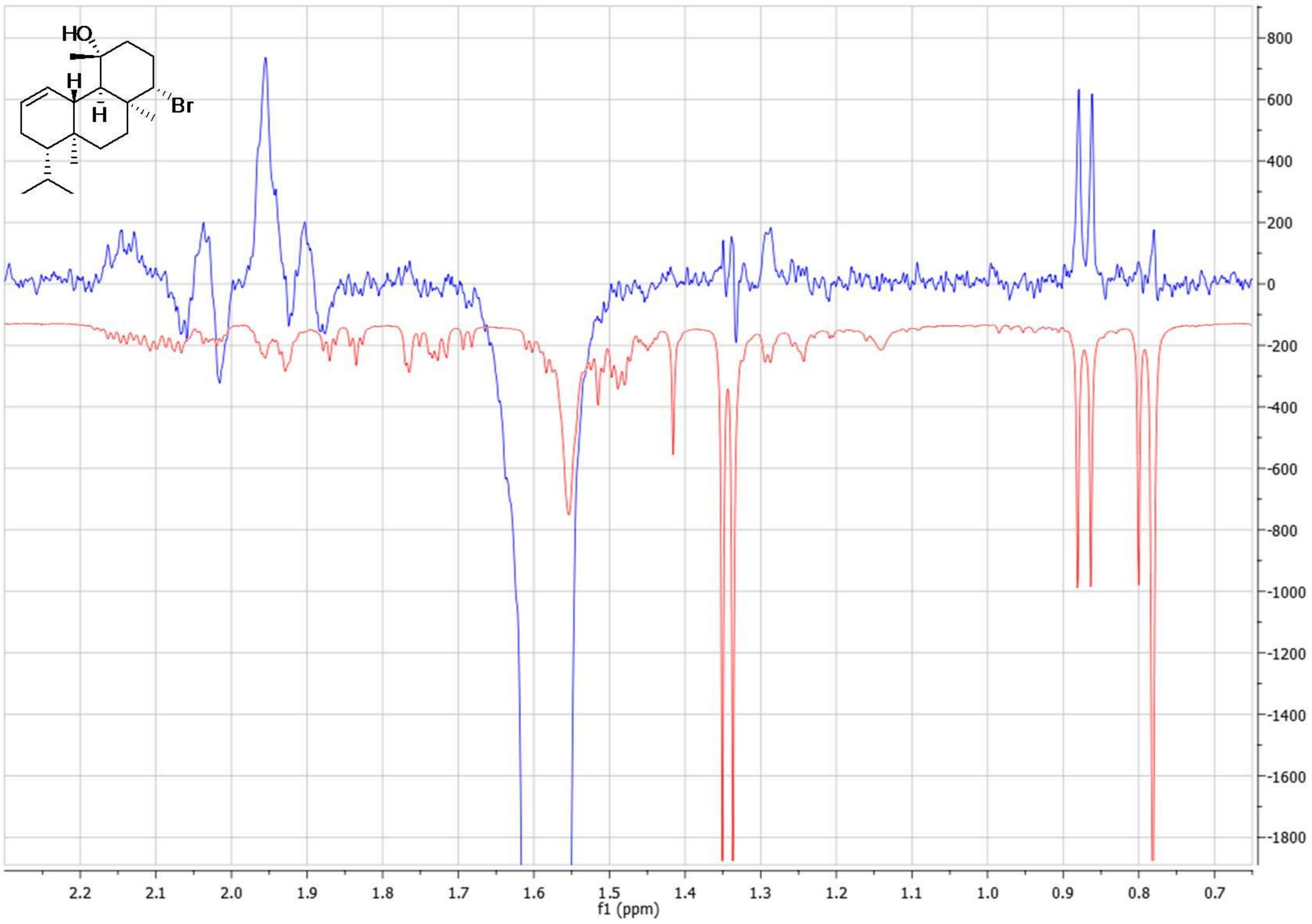

Figure S59. 1D NOE spectrum (400 MHz, $CDCl_3$), excitation of H-12 of debromosphaerol (**7**).

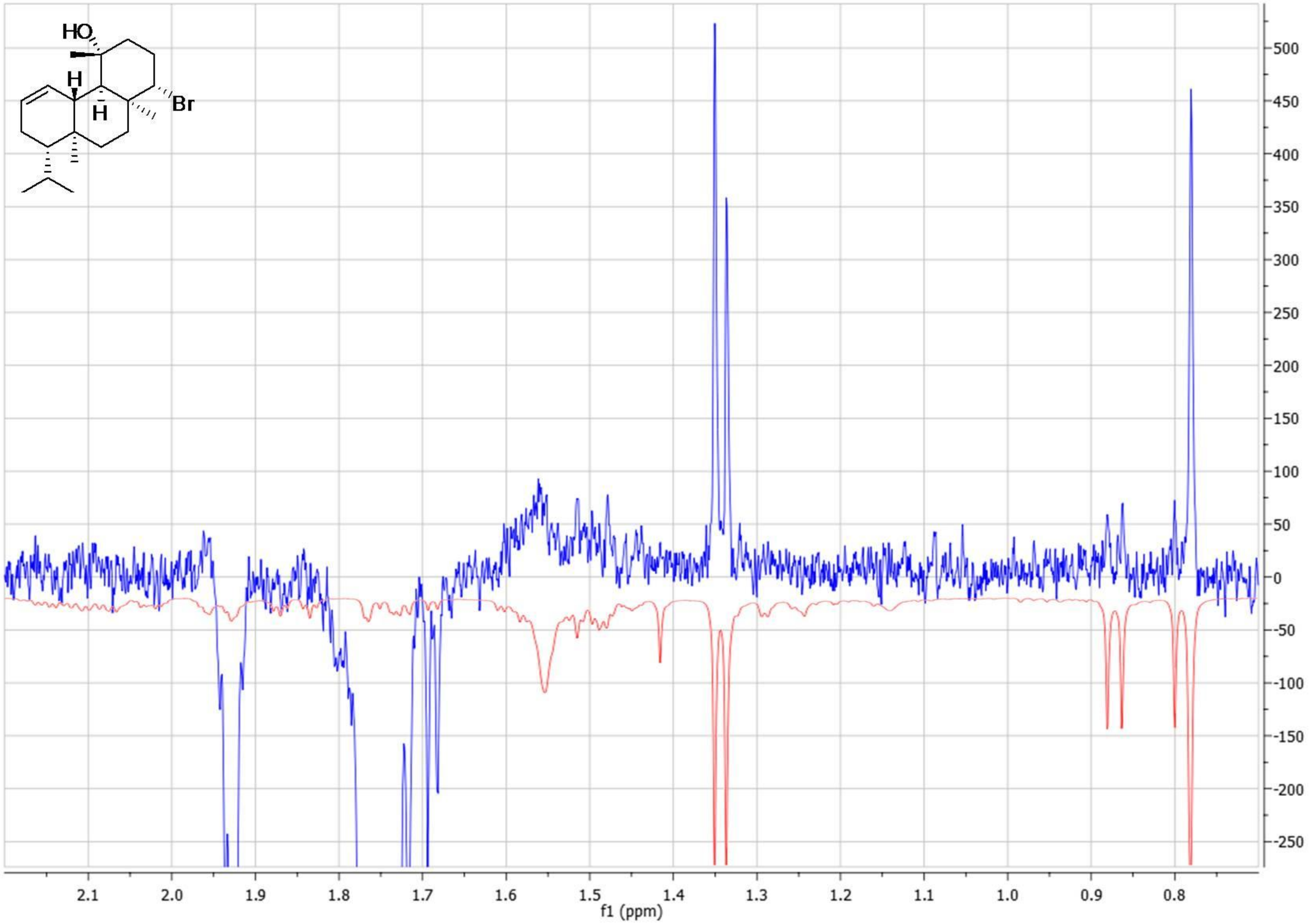

Figure S60. 1D NOE spectrum (400 MHz, $CDCl_3$), excitation of H-13 and H-2α of debromosphaerol (**7**).

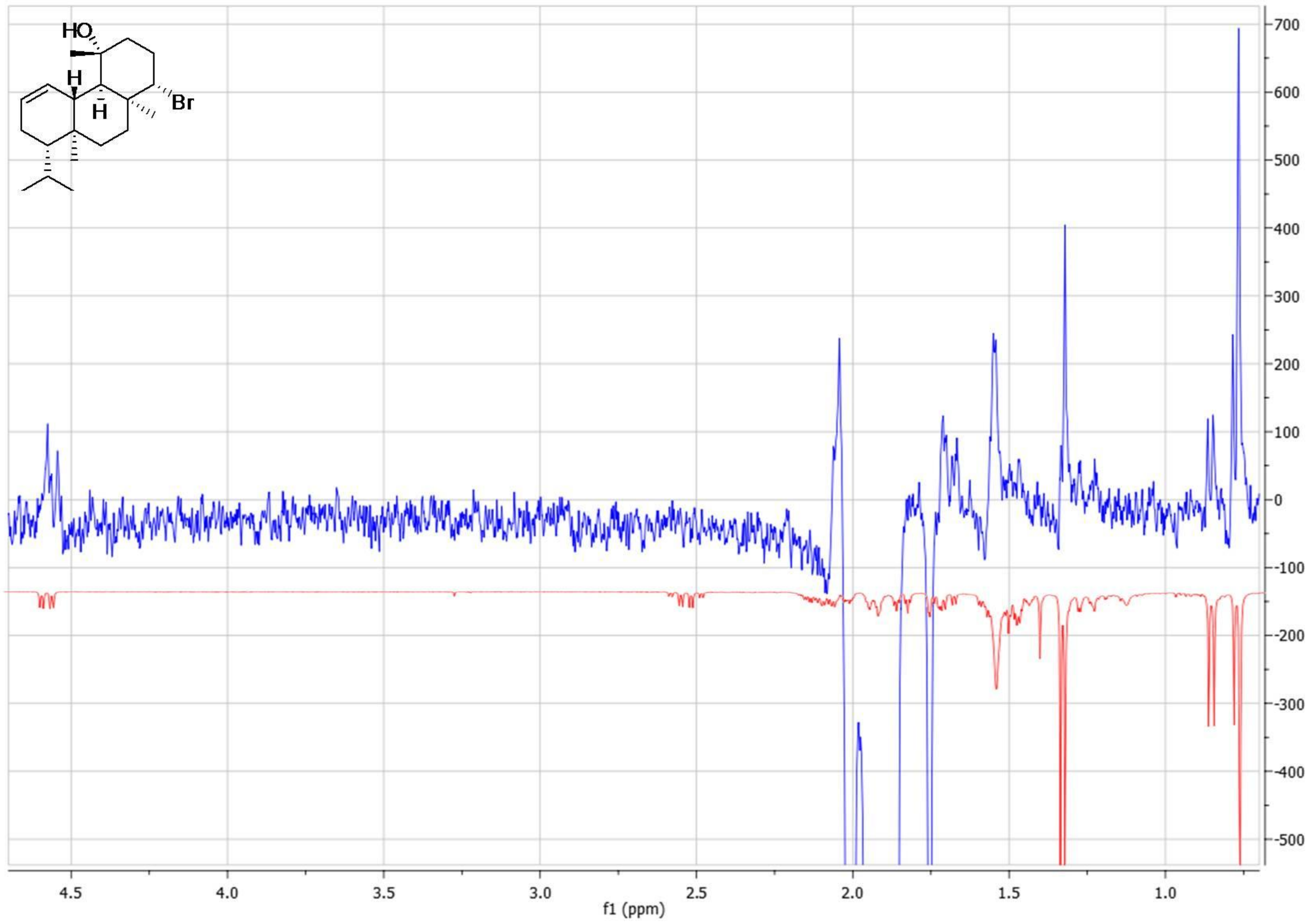

Figure S61. HRMS (ESI+) measurement of debromosphaerol (**7**).

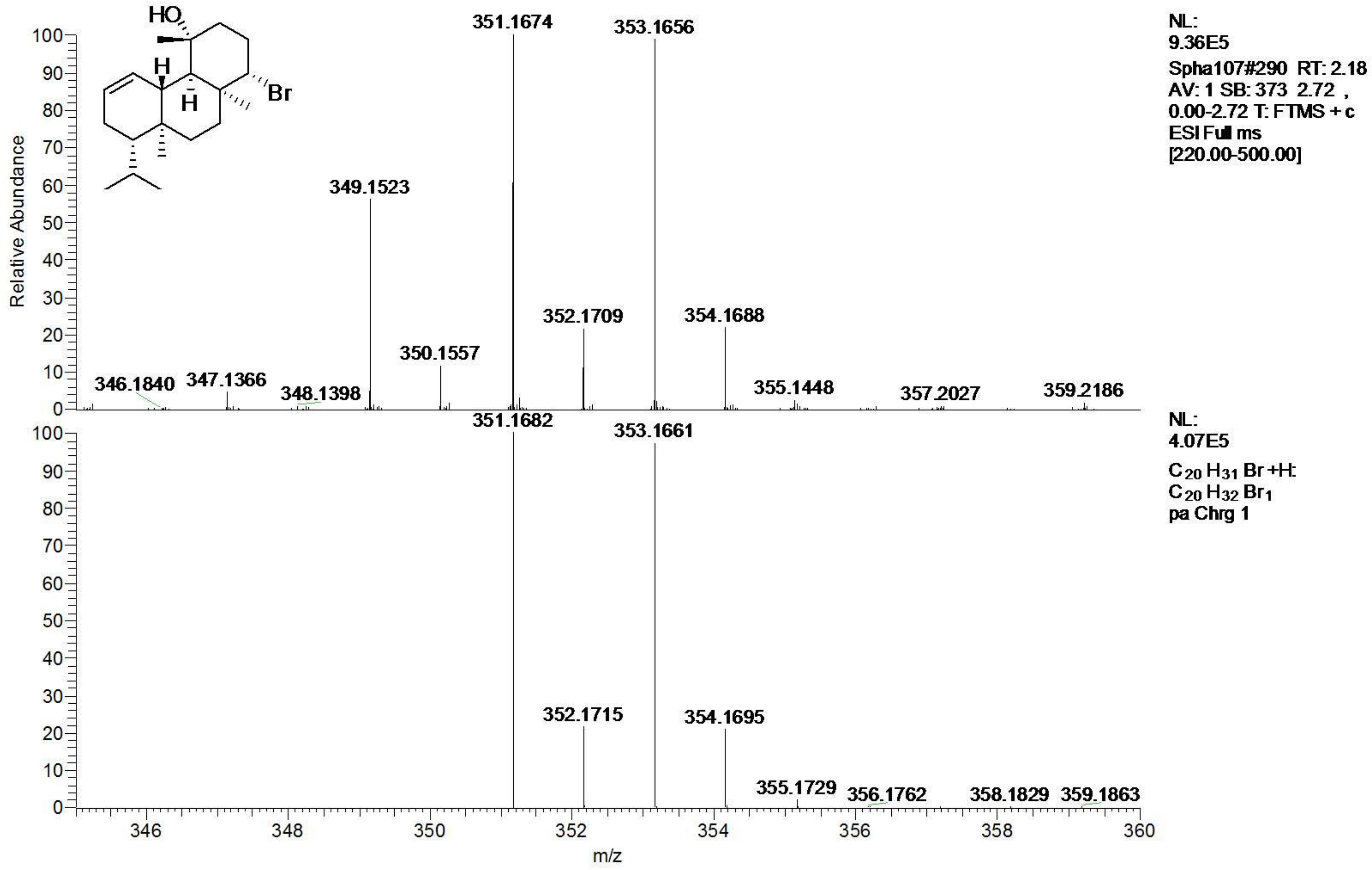

Figure S62. IR spectrum of debromosphaerol (**7**).

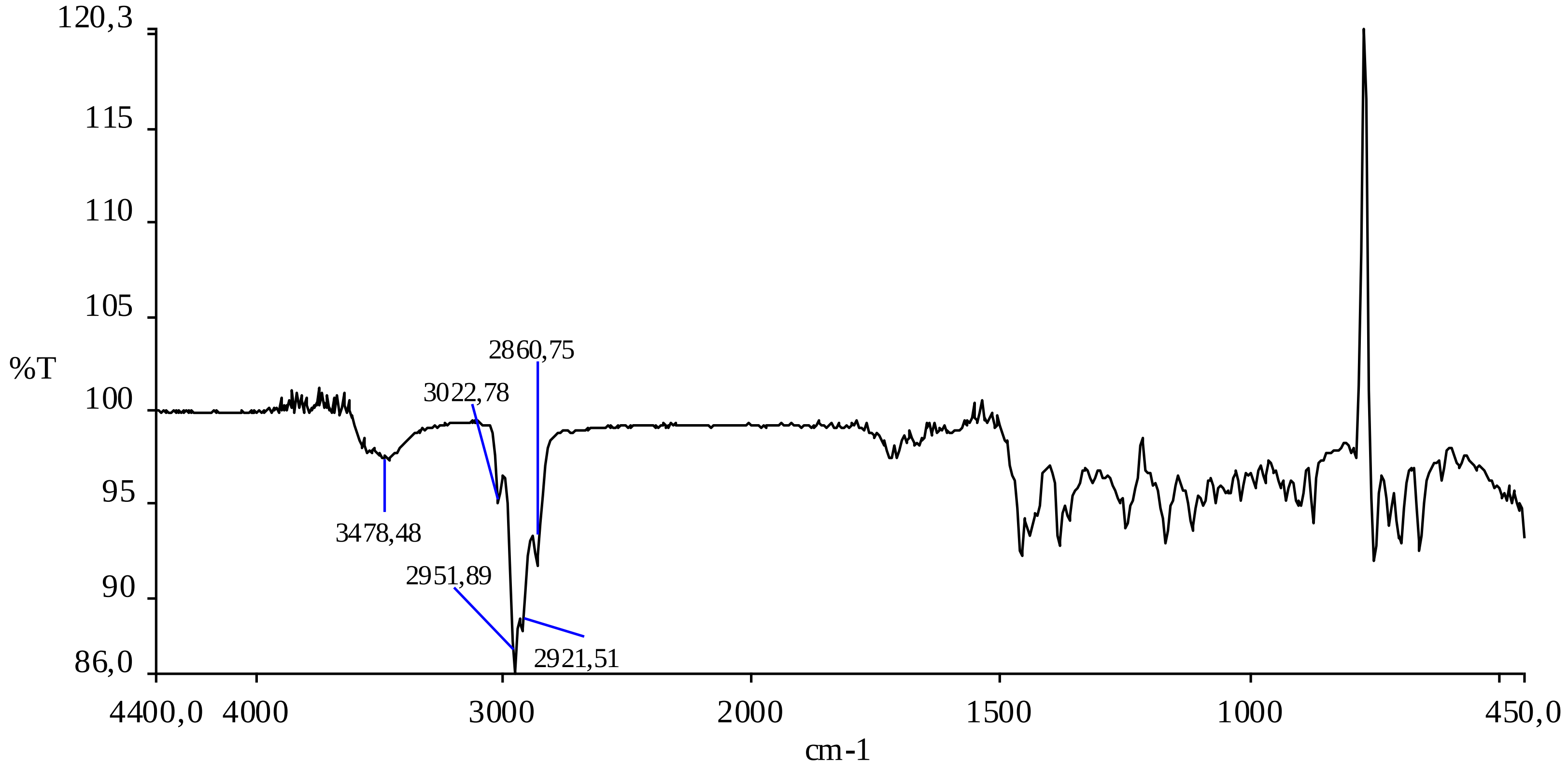

Figure S63. ^{1}H NMR spectrum (400 MHz, $CDCl_3$) of 8-methoxy-dihydro-sphaerococcenol (**8**).

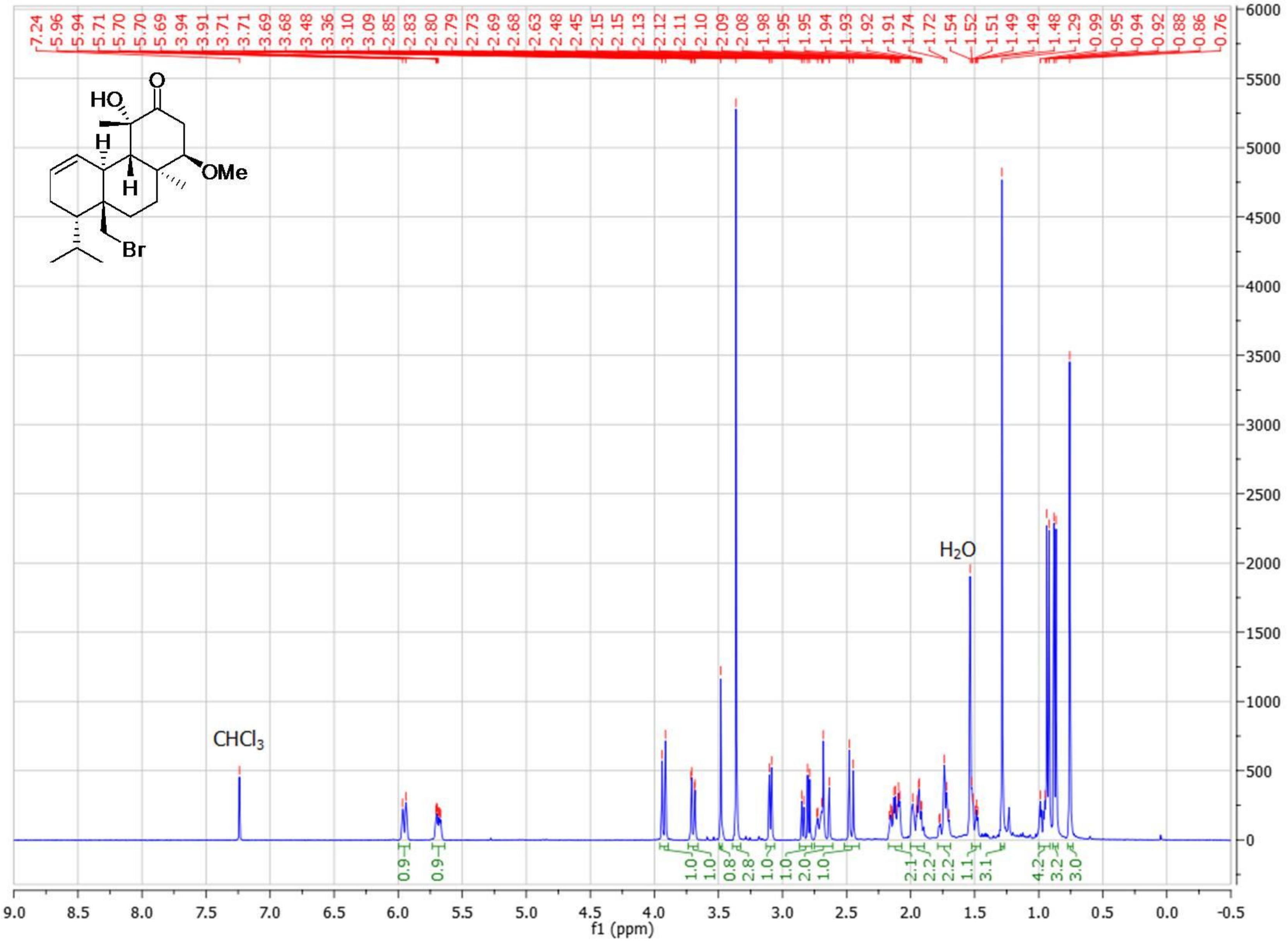

Figure S64. ^{13}C NMR spectrum (50 MHz, $CDCl_3$) of 8-methoxy-dihydro-sphaerococcenol (**8**).

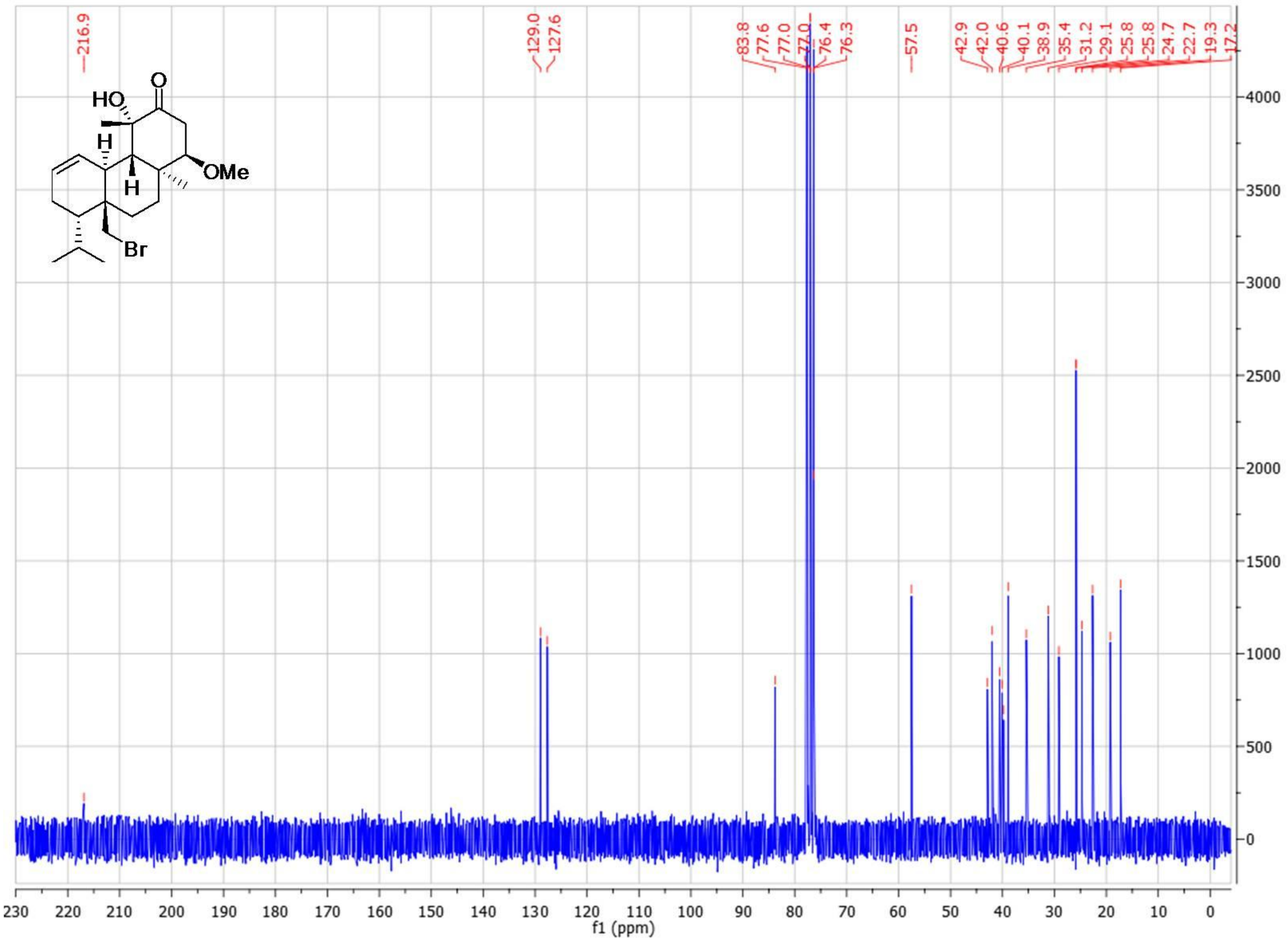

Figure S65. DEPT-135 spectrum (50 MHz, $CDCl_3$) of 8-methoxy-dihydro-sphaerococcenol (**8**).

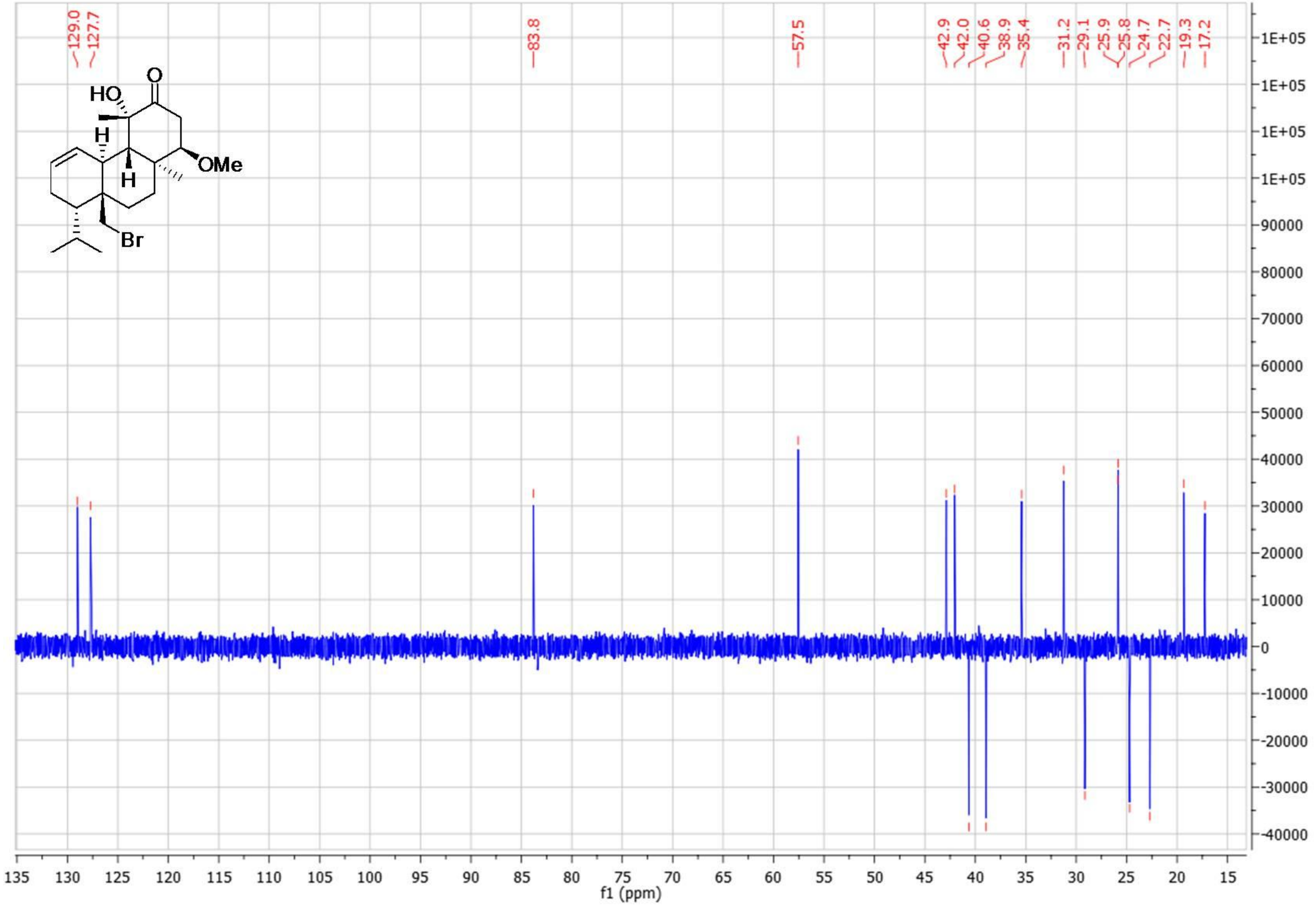

Figure S66. COSY spectrum (400 MHz, $CDCl_3$) of 8-methoxy-dihydro-sphaerococcenol (**8**).

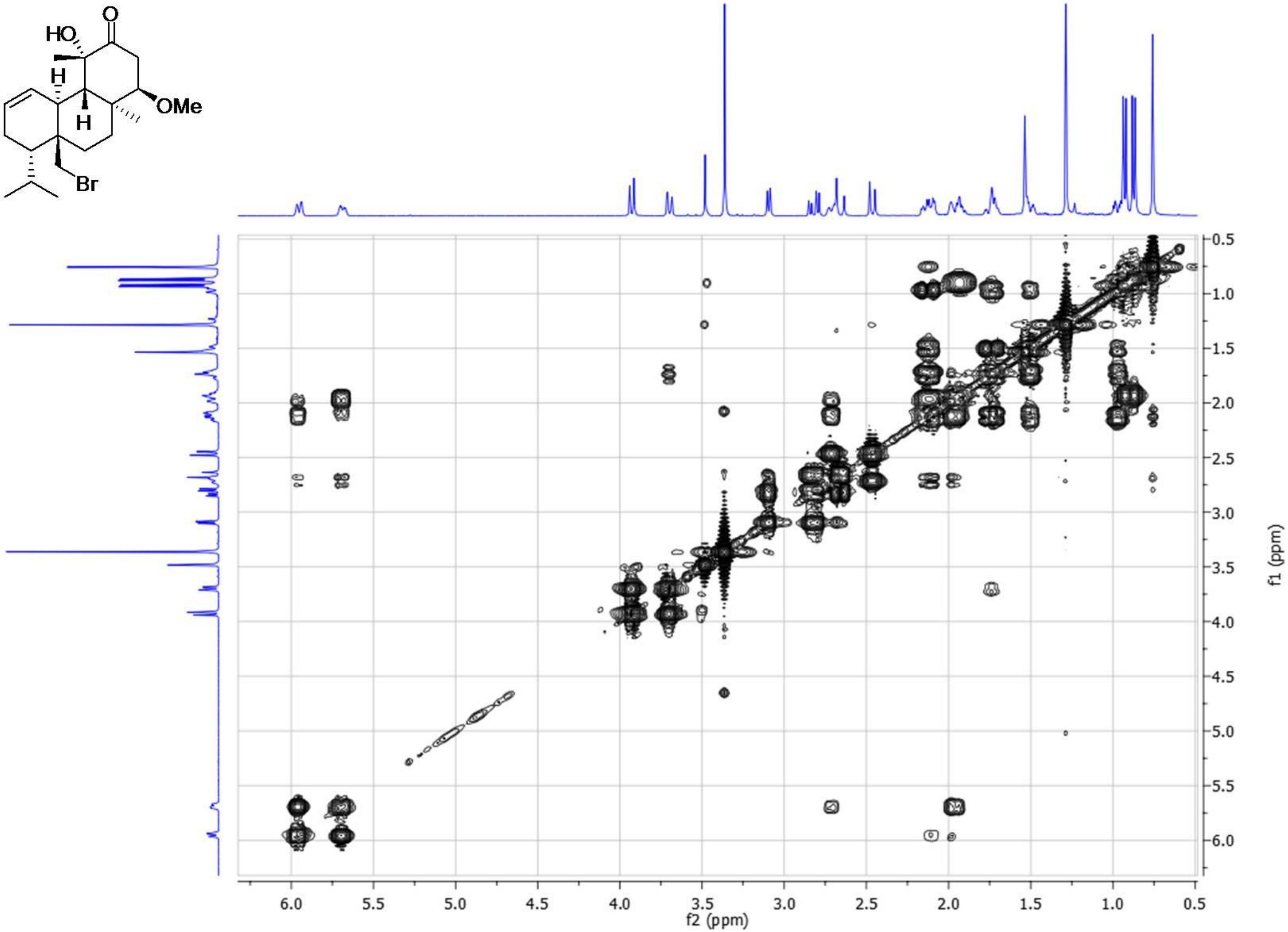

Figure S67. HSQC spectrum (400 MHz, $CDCl_3$) of 8-methoxy-dihydro-sphaerococcenol (**8**).

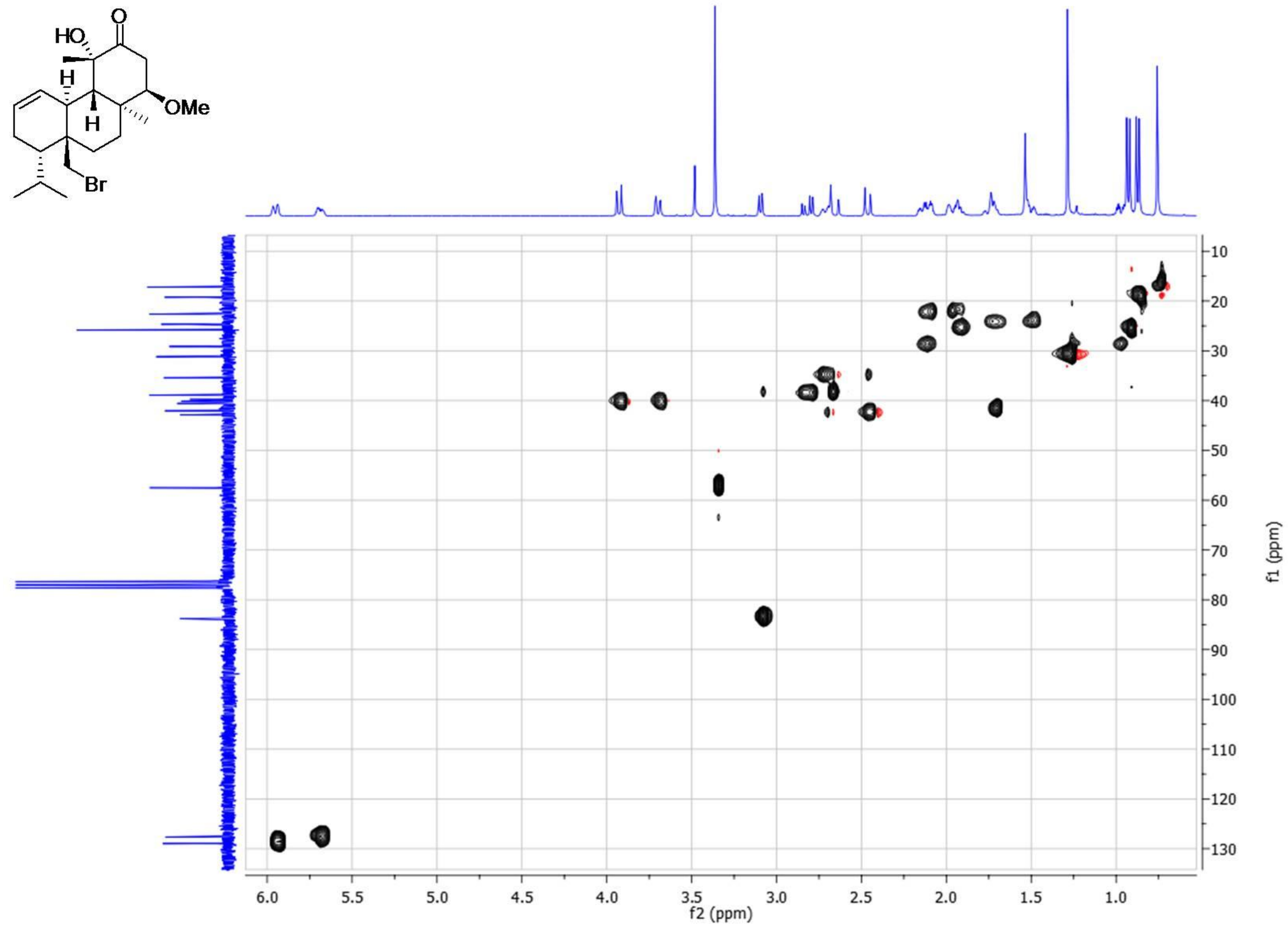

Figure S68. HMBC spectrum (400 MHz, $CDCl_3$) of 8-methoxy-dihydro-sphaerococcenol (**8**).

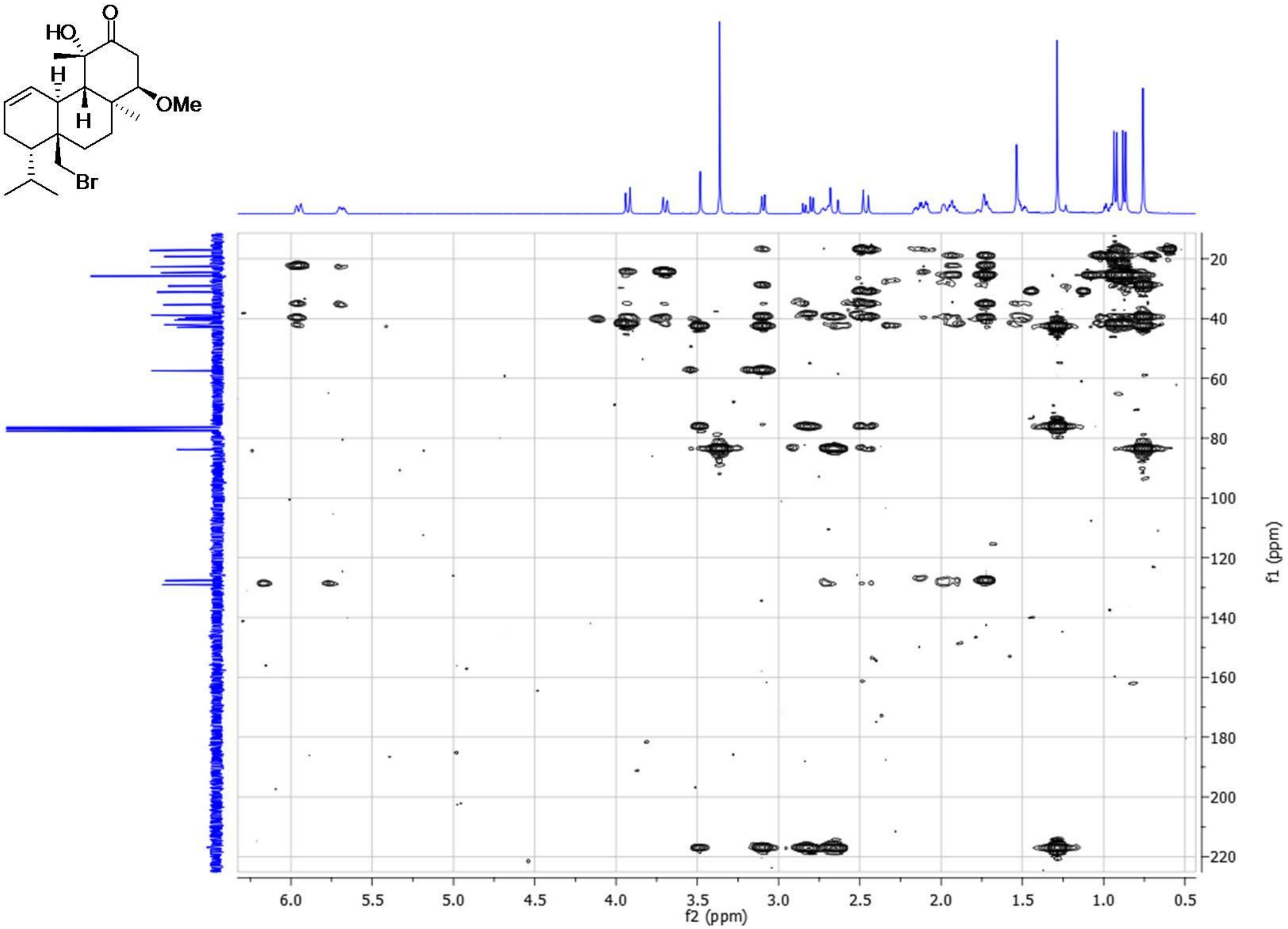

Figure S69. NOESY spectrum (400 MHz, $CDCl_3$) of 8-methoxy-dihydro-sphaerococcenol (**8**).

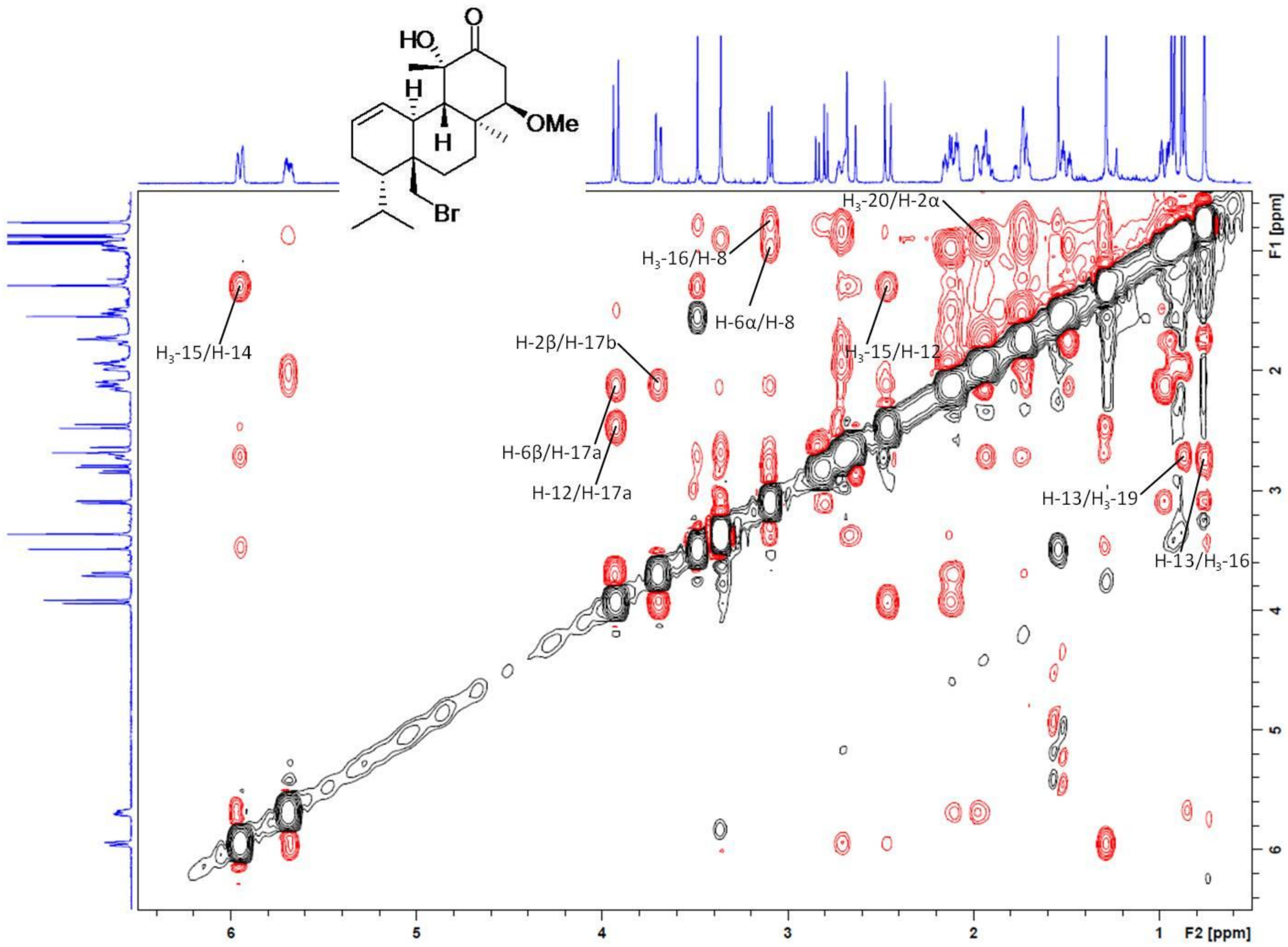

Figure S70. Chair conformation of 8-methoxy-dihydro-sphaerococcenol (**8**), energy: 59.57 Kcal/mole.

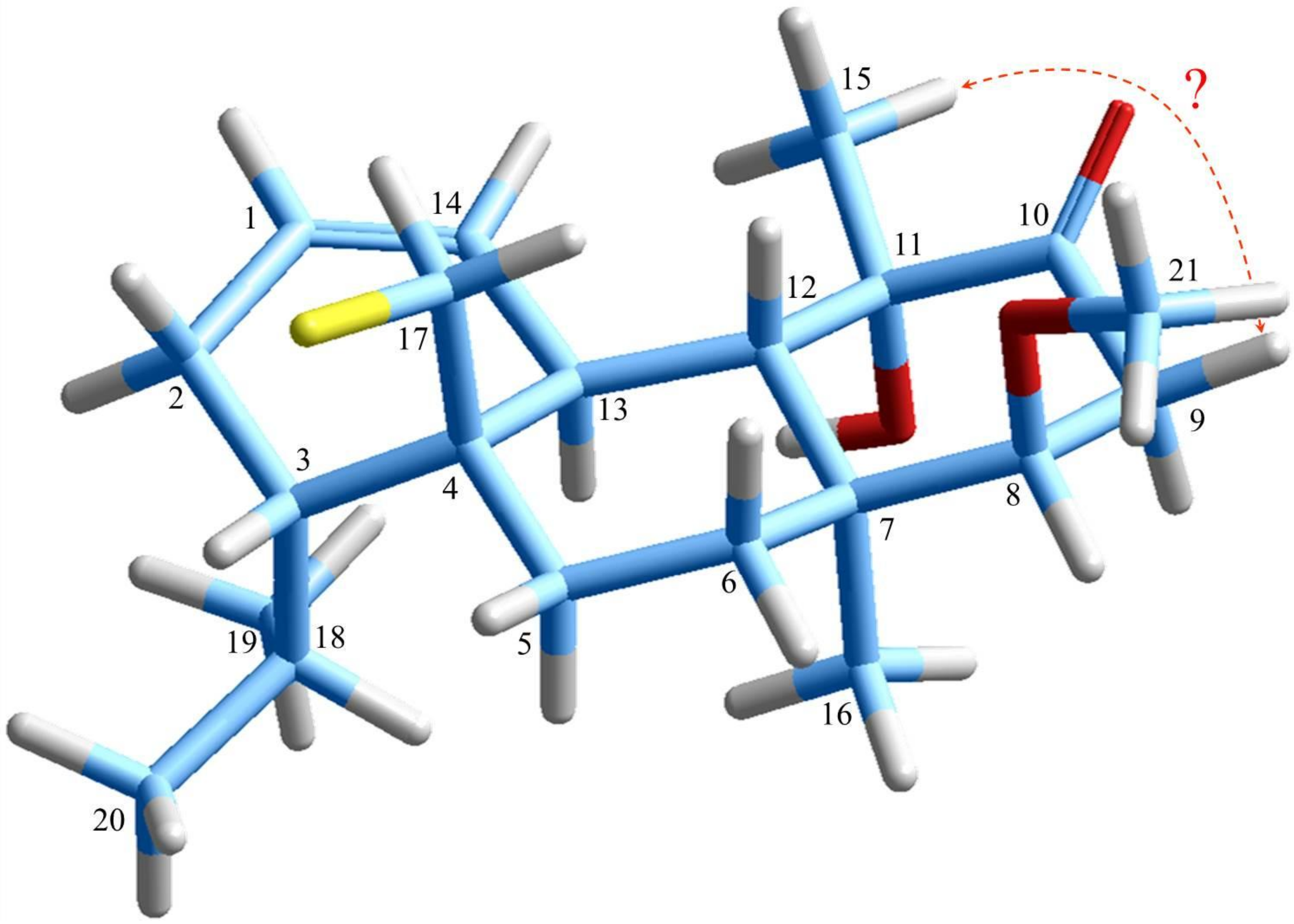

Figure S71. HRMS (ESI+) measurement of 8-methoxy-dihydro-sphaerococcenol (**8**).

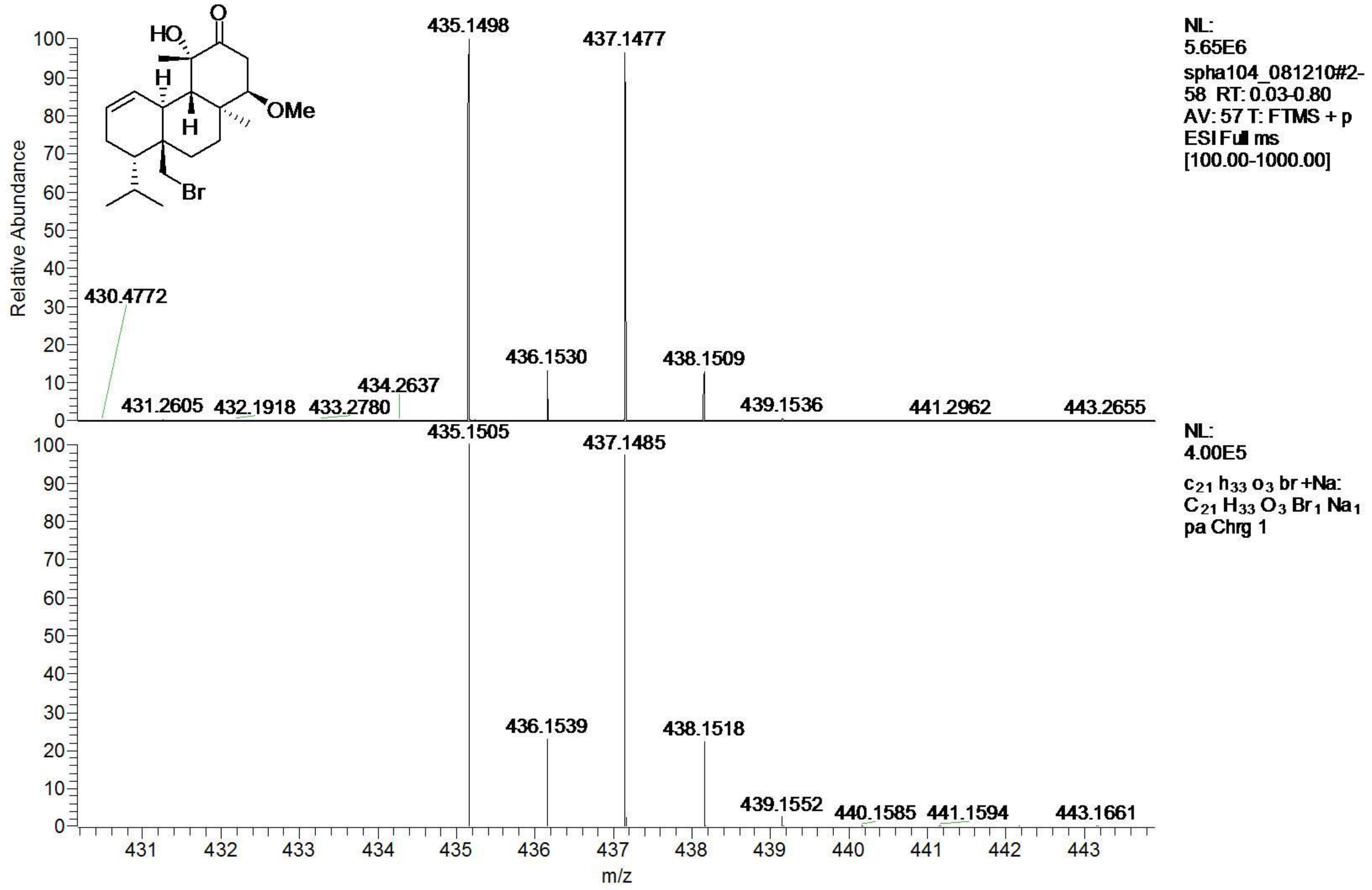

Figure S72. IR spectrum of 8-methoxy-dihydro-sphaerococcenol (**8**).

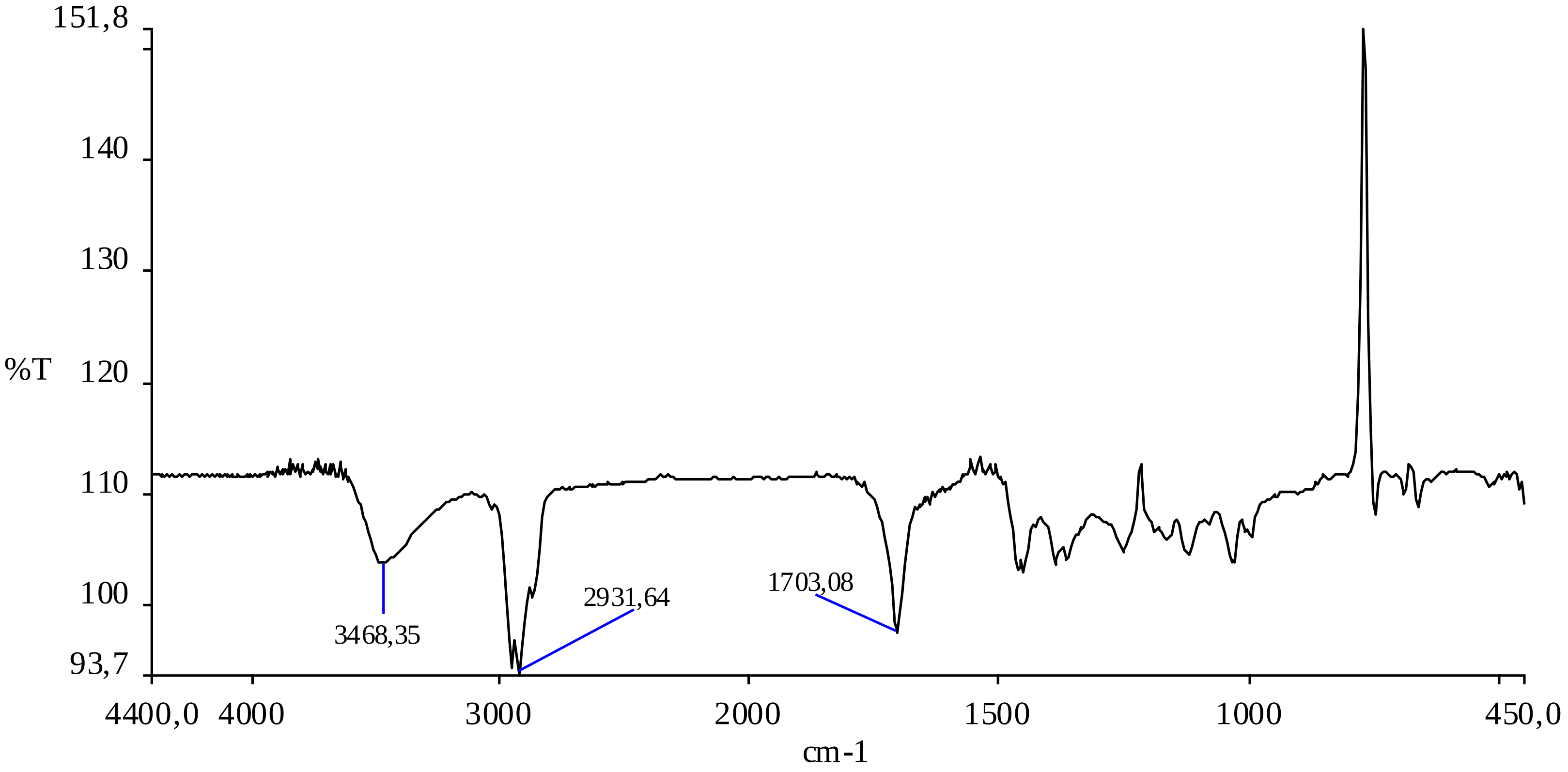

Supplement: Supplementary file 1 [file marinedrugs-18-00029-s001.pdf]
